# Association of latent class analysis-derived multimorbidity clusters with adverse health outcomes in patients with multiple long-term conditions: comparative results across three UK cohorts

**DOI:** 10.1016/j.eclinm.2024.102703

**Published:** 2024-06-28

**Authors:** Stefanie J. Krauth, Lewis Steell, Sayem Ahmed, Emma McIntosh, Grace O. Dibben, Peter Hanlon, Jim Lewsey, Barbara I. Nicholl, David A. McAllister, Susan M. Smith, Rachael Evans, Zahira Ahmed, Sarah Dean, Colin Greaves, Shaun Barber, Patrick Doherty, Nikki Gardiner, Tracy Ibbotson, Kate Jolly, Paula Ormandy, Sharon A. Simpson, Rod S. Taylor, Sally J. Singh, Frances S. Mair, Bhautesh Dinesh Jani, PERFORM research team

**Affiliations:** a General Practice and Primary Care, School of Health and Wellbeing, University of Glasgow, Glasgow, United Kingdom; b School of Allied and Public Health Professions, Canterbury Christ Church University, Canterbury, United Kingdom; c AGE Research Group, Translational and Clinical Research Institute, Faculty of Medical Sciences, Newcastle University, Newcastle upon Tyne, United Kingdom; d NIHR Newcastle Biomedical Research Centre, Newcastle upon Tyne NHS Foundation Trust, Cumbria, Northumberland, Tyne and Wear NHS Foundation Trust and Newcastle University, Newcastle upon Tyne, United Kingdom; e Health Economics and Health Technology Assessment, School of Health and Wellbeing, University of Glasgow, Glasgow, United Kingdom; f MRC/CSO Social & Public Health Sciences Unit, School of Health and Wellbeing, University of Glasgow, Glasgow, United Kingdom; g Discipline of Public Health and Primary Care, Trinity College Dublin, Dublin, Ireland; h Department of Respiratory Sciences, University of Leicester, Leicester, United Kingdom; i University of Exeter Medical School, Exeter, United Kingdom; j School of Sport, Exercise and Rehabilitation Sciences, University of Birmingham, Birmingham, United Kingdom; k Clinical Trials Unit, University of Leicester, Leicester, United Kingdom; l Department of Health Science, University of York, York, United Kingdom; m Department of Cardiopulmonary Rehabilitation, University Hospitals of Leicester NHS Trust, Leicester, United Kingdom; n Institute of Applied Health Research, University of Birmingham, Birmingham, United Kingdom; o School of Health and Society, University of Salford, Manchester, United Kingdom; p Robertson Centre for Biostatistics, School of Health and Wellbeing, University of Glasgow, Glasgow, United Kingdom

**Keywords:** Multimorbidity, Clustering, Hospitalisation, Mortality, Service use, Primary health care

## Abstract

**Background:**

It remains unclear how to meaningfully classify people living with multimorbidity (multiple long-term conditions (MLTCs)), beyond counting the number of conditions. This paper aims to identify clusters of MLTCs in different age groups and associated risks of adverse health outcomes and service use.

**Methods:**

Latent class analysis was used to identify MLTCs clusters in different age groups in three cohorts: Secure Anonymised Information Linkage Databank (SAIL) (n = 1,825,289), UK Biobank (n = 502,363), and the UK Household Longitudinal Study (UKHLS) (n = 49,186). Incidence rate ratios (IRR) for MLTC clusters were computed for: all-cause mortality, hospitalisations, and general practice (GP) use over 10 years, using <2 MLTCs as reference. Information on health outcomes and service use were extracted for a ten year follow up period (between 01^st^ Jan 2010 and 31st Dec 2019 for UK Biobank and UKHLS, and between 01^st^ Jan 2011 and 31st Dec 2020 for SAIL).

**Findings:**

Clustering MLTCs produced largely similar results across different age groups and cohorts. MLTC clusters had distinct associations with health outcomes and service use after accounting for LTC counts, in fully adjusted models. The largest associations with mortality, hospitalisations and GP use in SAIL were observed for the “*Pain+*” cluster in the age-group 18–36 years (mortality IRR = 4.47, hospitalisation IRR = 1.84; GP use IRR = 2.87) and the “*Hypertension, Diabetes & Heart disease*” cluster in the age-group 37–54 years (mortality IRR = 4.52, hospitalisation IRR = 1.53, GP use IRR = 2.36). In UK Biobank, the “*Cancer, Thyroid disease & Rheumatoid arthritis*” cluster in the age group 37–54 years had the largest association with mortality (IRR = 2.47). Cardiometabolic clusters across all age groups, pain/mental health clusters in younger groups, and cancer and pulmonary related clusters in older age groups had higher risk for all outcomes. In UKHLS, MLTC clusters were not significantly associated with higher risk of adverse outcomes, except for the hospitalisation in the age-group 18–36 years.

**Interpretation:**

Personalising care around MLTC clusters that have higher risk of adverse outcomes may have important implications for practice (in relation to secondary prevention), policy (with allocation of health care resources), and research (intervention development and targeting), for people living with MLTCs.

**Funding:**

This study was funded by the 10.13039/501100000272National Institute for Health and Care Research (NIHR; Personalised Exercise-Rehabilitation FOR people with Multiple long-term conditions (multimorbidity)—NIHR202020).

**Research in context**

**Evidence before this study**

On January 15, 2023 we searched PubMed for articles published for any date, using MeSH terms for “multimorbidity”, “cluster”, and “latent class analysis”. Our search returned 157 articles, after excluding duplicates. Screening for papers relating clusters to health outcomes and service use, we excluded 130 articles for lack of relevance and 18 articles with results specific either to an index condition or to a constrained population subgroup (not related to age). Eight articles conducted latent class analysis (LCA) on a general population sample to define MLTC clusters and related them to health and/or service use outcomes, three using data from the USA, and one each from Italy, Spain, Portugal, Taiwan, and the UK. Five of these were specifically focused only on older age groups. Out of the three articles that used a broader adult population i.e. >18 years, only one stratified MLTC clusters by age categories. All the included studies utilised only one data source with no external validation and none of the previous eight studies studied the association of MLTC clusters with risk of adverse health outcomes or service use, after adjusting for the effects of number of conditions.

**Added value of this study**

This study compares clusters of conditions in people with MLTCs across four different age groups. It is the first study to examine how MLTC clusters differ in their association with mortality, hospitalisations and general practice use across multiple datasets after adjusting for the number of LTCs. This enables us to elucidate the importance of MLTC clusters as a concept in addition to simple counts of LTCs, when considering risk of adverse health outcomes and levels of healthcare utilisation. Largely similar MLTC clusters were identified between databanks and age groups. Our analysis shows that MLTC clusters of LTCs have significant associations with adverse health outcomes and service use, even after adjusting for sociodemographic/lifestyle factors and LTC count.

**Implications of all the available evidence**

Clusters identified in the literature show similarities in which conditions are grouped together and clusters grouping cardiometabolic diseases, respiratory conditions, psychiatric/neurological conditions, and painful conditions are common. Certain MLTC clusters, such as cardiometabolic clusters, are consistently associated with adverse health outcomes and increased service use, although clustering in different age groups show differences between younger and older adults. Our findings illustrate that considering MLTC clusters and LTC counts in analyses provides a more comprehensive assessment of the true association of MLTC clusters with health outcomes, and could inform decision making around healthcare service design and resourcing and be important for clinical practice and multimorbidity intervention research in the future.

## Introduction

There is a growing number of people living with multimorbidity (>2 long-term conditions (LTCs), hereafter referred to as multiple long-term conditions (MLTCs).^1^ Global evidence suggests that more than one third of the world's general population lives with MLTCs (37.2%) and that this proportion is increasing.^2^ Recent UK evidence based on healthcare data estimates the prevalence of MLTCs in England at 29%.^3^ Importantly, data indicates that there is a relatively higher mortality risk in younger individuals, and increased prevalence in deprived areas.^4^^,^^5^ MLTCs are associated with a reduced quality of life (QoL), functional decline, increased mortality, and increased healthcare utilisation, including emergency admissions.^1^^,^^6^ People living with MLTCs experience increased symptom burden and disability and single disease-based care which is not person centred and exacerbates treatment burden (the workload of self-management).^7^^,^^8^ Due to this increasing burden, targeted interventions, which are effective and cost-effective, are needed for people living with MLTCs.

Despite this background, a systematic review of intervention trials targeting people with MLTCs has generally not demonstrated cost effectiveness and achieved little to no improvement in patient outcomes ranging from clinical outcomes (e.g. blood pressure, glycaemic control), to health related QoL, health service utilisation, or medication use for either co- or multimorbidity.^9^ A more recent systematic review, specifically looking at trials for MLTCs (as opposed to comorbidity), found little to no evidence for the effectiveness or cost-effectiveness of interventions on primary outcomes.^10^ The reviewers noted significant variations in study participants and trial design. There was some suggestion that interventions for people with MLTCs, focused upon physiotherapy and improving functional capacity to support physical activity and achievement of activities of daily living may be more effective.^9^^,^^11^ Despite this, there have, to date, been no published RCTs reporting exercise-based interventions for people living with MLTCs. The NIHR-funded research programme on personalised exercise-rehabilitation for people with MLTCs (PERFORM) aims to develop an integrated approach to rehabilitation, that will include a structured programme of supervised exercise training and self-management support.^12^

Evidence on the best possible way to classify people with MLTCs remains ambiguous.^4^^,^^13^, ^14^, ^15^ A systematic review of 51 studies that examined the prevalence of MLTC clusters found that latent class analysis (LCA) and cluster analysis were more suitable approaches when the diseases were treated as discrete entities.^13^ We propose to use LCA across different age groups, to identify clusters of LTCs that tend to frequently occur together and analyse these MLTC clusters to identify those groups of MLTCs with the highest risk of adverse health outcomes and health care service use. Stratifying by age ensures that we do not treat people of all ages as homogenous in terms of their LTC profiles.

The specific objectives of this study are:

1. To analyse the association between clusters of MLTCs (defined using LCA) in different age groups to health outcomes and service use including mortality, hospitalisations, and General Practitioner (GP) use, using three UK population datasets when compared to those with no MLTCs.
2. To investigate what additional information can be gained from using MLTC clusters in addition to counts of LTCs in understanding the risk of adverse health outcomes and service use in patients with MLTCs.
3. To understand which MLTC clusters have the highest overall associated absolute risk of mortality, hospitalisations, and GP use.

## Methods

### Study sample

The study was performed using data from three cohorts: UK Biobank, Secure Advanced Data Linkage (SAIL) Databank in Wales, and the UK longitudinal Health Survey (UKHLS). Multimorbidity (MLTCs) was defined as having two or more LTCs at baseline. A list of all LTCs included, as well as details on all variables extracted or constructed from each databank is given in Supplement 1, Table S1.1.

The UK Biobank cohort comprises more than 500,000 participants aged between 37 and 73 years recruited from England, Scotland, and Wales between 2006 and 2010. UK Biobank participants were registered with a GP and completed a touchscreen questionnaire and nurse-led interview as well as physical examinations at baseline. We extracted data on presence/absence of 43 self-reported LTCs at baseline.^16^ Additionally, we extracted data on sex, age, area deprivation (Townsend score^17^), smoking, alcohol consumption, ethnicity, frailty (please see Supplement 1 for definition), self-rated health, body mass index (BMI), and physical activity from the same baseline assessment. All demographic and lifestyle information were self-reported by participants. Participants’ data were linked to hospital episode statistics and death registries and, for a sub-set of participants (approximately 45%) to primary care data, to collect data on health outcomes.

The SAIL databank comprises anonymised routine and emergency health care data for Wales by linking primary care, hospital, mortality, and demographic data from general practices and hospitals in Wales which cover approximately 70% of the Welsh population.^18^ SAIL data is representative of the Welsh population.^19^ In SAIL we extracted LTCs for individuals aged 18+ based on Read codes and prescription data in individual's linked primary care data recorded by 01^st^ Jan 2011 as described in Hanlon et al., 2022.^20^ The baseline date was chosen based on the availability and completeness of electronic records and similarity to the UK Biobank baseline assessment. Read codes were mapped onto the list of 43 LTCs used in UK Biobank (Supplement 1, Table S1.1).^20^ Additionally, we extracted data on sex, age, Welsh index of multiple deprivation (WIMD), and smoking status at baseline from the SAIL databank.^20^

UKHLS is a longitudinal household panel survey of approximately 51,000 participants recruited from the general population in the UK.^21^ We extracted LTC presence/absence based on questionnaire responses about 17 pre-determined LTCs individuals had been asked about at baseline. To map this list to the LTCs coded for UK Biobank and SAIL, some conditions were combined into a single condition leading to a total of 13 LTCs which were used for this analysis (Supplement 1, Table S1.1). Data on hospitalisations and GP use were based on self-reported number of events during questionnaire administration. No mortality data was available in UKHLS. Additional data on sex, age, ethnicity, BMI was collected. Data for alcohol consumption, physical activity and smoking status were also extracted from wave 2 of UKHLS data collection as they were not available at baseline.^21^ The data collection for wave 1 was from Jan 2009 to March 2011 and wave 2 was from Jan 2010 to March 2012. Details on the coding and availability of variables in each databank can be seen in Supplement 1, Table S1.2.

### Outcome data

Information on health outcomes and service use were extracted for a ten year follow up period, which was between 01^st^ Jan 2010 and 31st Dec 2019 for UK Biobank and UKHLS, and between 01^st^ Jan 2011 and 31st Dec 2020 for SAIL. Outcome data extracted included: mortality (all-cause mortality based on death registry, hospitalisations (hospitalisation episodes, days spent in hospital), and GP use. Data availability was as follows: All-cause mortality: based on data from death registries for UK Biobank and SAIL (all participants). No mortality data from UKHLS. Hospitalisations: based on healthcare records for UK Biobank (all participants) and SAIL (all participants). Based on self-reported number of admissions from UKHLS (for a subset of participants who chose to answer related questions, and available for five out of the ten years follow-up). GP use: Approximated by the number of days where a Read-code was entered by a primary care provider for UK Biobank (approximately 230,000 (45%) of participants) and SAIL (all participants). Based on self-reported use of GP for UKHLS (for a subset of participants who chose to answer related questions, and available for five out of the ten years follow-up). Details on the coding and availability of variables in each databank can be seen in Supplement 1.

### Statistical analysis

The analysis was stratified into four different age-groups: 18–36 years (available only in SAIL & UKHLS), 37–54 years (available in all databanks), 55–73 years (all databanks) and 74+ years (SAIL, UKHLS). LCA was performed in R using the *poLCA* package^22^ in each age-group for people with MLTCs (≥2 LTCs). Using presence/absence of LTCs (N = 43 LTCs in UK Biobank and SAIL; N = 13 LTCs in UKHLS), a series of LCA models were explored containing one to ten latent classes for each age group/dataset combination (n = 10). Optimal LCA model selection was based primarily on model parsimony (using the Bayesian information criterion [BIC] and sample size-adjusted BIC [aBIC]), with consideration of model classification (entropy) and substantive clinical interpretation of the model solution. BIC was selected due to its improved reliability over the Akaike Information Criteria in large sample sizes. MLTC clusters were labelled according to within and between-cluster prevalence of single-condition LTCs. Considering the large number of 38 MLTC clusters discussed in this paper, we decided to use cluster names and have given a detailed narrative explanation of the rules that were applied for naming the MLTC clusters, and the reason for each individual name. An overview of the disease profiles for each MLTC cluster can be found in Supplement 2. MLTC clusters were named based on LTCs with the highest between-cluster and within-cluster prevalence in each age-group and Databank. Where the LTCs in a cluster affected a common system, that system was named in the cluster (e.g. pulmonary). The symbol + was added to a cluster name with one predominant LTC to indicate that other LTCs were also prevalent in this MLTC cluster. A detailed naming rationale for each MLTC cluster can be found in Supplement 2. For accuracy, we will refer to MLTC clusters by first indicating the databank (S for SAIL, B for UK Biobank, & U for UKHLS), followed by the lower age of the age range (18 for 18–36, 37 for 37–54 etc.) and then the cluster name. Individuals were assigned to one MLTC cluster based on their respective posterior probabilities and each individual could only be assigned to one unique MLTC cluster. Members of the same cluster differ in their exact LTC profile and numbers of LTCs based on prevalence of LTCs in the given cluster. A detailed description of the LCA methods and outcomes are available in Supplement 3.

To describe socio–demographic profiles of participants in each databank we present absolute numbers per group for sex, age-group, and deprivation score for each databank. Mean mortality rates, hospitalisations, and GP visits were computed for each group.

To understand the association of MLTC clusters and condition counts with the outcome, we computed a count-based variable based on presence/absence of LTCs into: 0–1 LTC (reference group), 2 LTCs, 3 LTCs and 4 LTCs and >4LTCs in SAIL and UK Biobank. In UKHLS LTC counts were grouped 0–1 LTC, 2 LTCs, 3 LTCs and 4+ LTCs based on a better model fit. Participants without MLTCs (0–1 LTCs) were used as a reference group for statistical analysis of the association of MLTC clusters with mortality, hospitalisations, and GP use.

All-cause mortality was used as a dichotomous variable at the end of the follow-up period and average mortality rates over the 10-year follow up were compared between groups. Hospitalisation events were defined as count of total unique hospital admissions during the 10-year follow-up and average hospitalisation rates were compared between groups. Hospital admissions were counted as separate episodes if they were at least 24 h apart. Length of hospitalisation was computed as total number of days spent in hospital during the 10-year follow up period. The proxy for GP use in SAIL and UK Biobank was defined as the count of total number of days during the 10-year follow-up where any Read codes were entered by a primary care provider and average rates of GP visits were compared between groups. In UKHLS, number of hospitalisations, days spent in hospital, and GP use were based on self-reported numbers during the follow-up period and average rates of GP visits were compared between groups.

Statistical analyses were performed in Stata 17.^23^ Overdispersion was assessed using alpha-statistics with alpha-levels close to zero indicating no excess overdispersion. Negative binomial regression was employed in case of higher alpha values to account for overdispersion. Deviance and Pearson goodness of fit were used to assess model fit in Poisson regression and log likelihood and likelihood ratio statistics for negative binomial regression. Negative binomial models were used to analyse the relationship of MLTC clusters and LTC counts with 1) number of hospitalisations and 2) GP use, and Poisson regression was used to analyse the relationship of latent class-membership and LTC counts with 3) mortality, adjusting for demographic and lifestyle factors as outlined below. Factors considered were: sex, age, deprivation (WIMD score), and smoking status in SAIL; sex, age, area deprivation (Townsend score), smoking, alcohol consumption, ethnicity, frailty, self-rated health, body mass index (BMI), and physical activity in UK Biobank, and sex, age, ethnicity, BMI, alcohol consumption, physical activity, and smoking in UKHLS. Non-linearity for quantitative variables was assessed by creating both, higher-order terms and cubic splines with 3, 4, and 5 knots distributed at equally spaced percentiles. The non-linear terms were tested by comparing their fit in separate univariate models using AIC and BIC, giving priority to BIC in case of disagreement. The most parsimonious non-linear terms were then included in the maximal model if the likelihood ratio test for their inclusion in the univariate model was significant at the 5% level. Maximal models were created with all variables, including identified non-linear terms, clusters, and MLTC counts and the inclusion of non-linear terms in the final model was decided using likelihood ratio testing at a significance level of 5%. All models were computed over a 10-year follow up period. An offset adjusting for difference in observation time based on death before the end of the follow-up period was used for the hospitalisation and GP-use regression models in SAIL and Biobank. A list of variables and their coding and availability in each database can be found in Supplement 1, Table S2. The associations between variables and outcomes were evaluated using incidence rate ratios (IRR) interpreted as difference in incidence rates compared to the non-MLTC group with 95% confidence intervals (95% CIs). In addition to the tables showing details for all three cohorts, predicted incidence rates (predicted marginal effect with all covariates set to the mean in the respective group) for MLTC clusters in different age groups are shown as graphs for the SAIL cohort, as this is the largest and most representative cohort, data used in our analysis.

To compare the association of MLTC clusters with health outcomes for SAIL and UK Biobank MLTC clusters from all age groups to each other and understand which MLTC clusters have the highest overall impact, we ranked all MLTC clusters by the predicted years of life lost. The predicted marginal affects for mortality in each were calculated with covariates set to their respective means. Ranking was done by calculating predicted years of life lost (YLL) for each MLTC cluster according to the World Health Organisation Global Health Observatory: **YLL(c,a) = N(c) x L(a)** where: **N(c)** = average number of deaths **N** in MLTC cluster **c** (based on predicted marginal effects during follow up), **L(a)** is a loss function specifying the years of life lost **L** for a death at a given age **a**, i.e. the average age of participants in this MLTC cluster, based on the UK national life expectancy in 2019 with a life expectancy at birth of 81.2 years.^24^ YLL were not stratified by sex. Then, MLTC clusters were ranked from largest (rank 1) to smallest (rank 38) based on this value. MLTC clusters were also ranked by number of days spent in hospital, and number of GP use (Supplement 4).

### Ethics and research governance

This study is part of an ongoing NIHR-funded Research project “Personalised exercise rehabilitation for people with multiple long-term conditions (PERFORM)”. The UK Biobank has full ethical approval from the NHS National Research Ethics Service (16/NW/0274). This study was conducted as part of UK Biobank Project 14,151. The use and analysis of SAIL data was approved by the SAIL information governance review panel (Project 0830). UKHLS data access and use was granted by the UK Data Service (Project ID: 221,571).

### Role of the funding source

The study was funded by the National Institute for Health and Care Research (NIHR; Personalised Exercise-Rehabilitation FOR people with Multiple long-term conditions (multimorbidity)—NIHR202020). The views expressed are those of the author(s) and not necessarily those of the NIHR or the Department of Health and Social Care.

## Results

### Study population

In UK Biobank, a total of 502,363 participants were included. At baseline, 165,157 (32.9%) were classed as having MLTCs, whereas 164,213 (32.7%) reported one LTC and 173,133 (34.4%) reported no LTCs. For all included participants, hospitalisation and mortality data were available, whereas data on GP use was available only for a linked subset of 226,754 participants. Female sex, older age and higher area deprivation were associated with higher odds for having MLTCs (Table 1).

**Table 1:** MLTCs and sociodemographic characteristics of UK Biobank, SAIL and UKHLS participants.

UK Biobank	Total	MLTCs (row %)	Median # of hospitalisations^d^	IQR (hosp.)	Median # GP visits^d^	IQR (GP visits)	Mortality rate in %	95% CI (mortality)
**Age**								
37–54 years	194,200	42,149 (21.7%)	2	1–4	37	21–63	2.08	2.02–2.15
55–73 years	308,303	123,008 (39.9%)	3	2–6	59	36–90	7.56	7.46–7.65
**Sex**								
Female	273,383	91,597 (33.5%)	3	1–5	52	31–82	4.06	3.99–4.13
Male	229,120	73,560 (32.1%)	3	1–6	48	25–79	7.09	6.99–7.20
**Townsend score**^a^								
1st quintile (least deprived)	100,658	29,938 (29.7%)	3	1–5	48	28–76	4.63	4.50–4.76
2nd quintile	100,097	31,093 (31.1%)	3	1–5	48	28–77	4.77	4.64–4.90
3rd quintile	100,383	31,950 (31.8%)	3	1–5	50	29–80	5.09	4.95–5.23
4th quintile	100,367	33,414 (33.3%)	3	1–6	50	28–81	5.43	5.29–5.57
5th quintile (most deprived)	100,998	38,762 (38.4%)	3	2–6	55	30–91	7.31	7.13–7.45

SAIL	Total	MLTCs (row %)	Median # of hospitalisations^d^	IQR (hosp.)	Median # GP visits^d^	IQR (GP visits)	Mortality rate in %	95% CI (mortality)
**Age**								
18–36 years	557,006	53,818 (9.7%)	2	1–4	59	23–123	0.67	0.65–0.69
37–54 years	579,940	139,943 (24.1%)	2	1–4	112	42–205	3.02	2.97–3.06
55–73 years	490,639	246,893 (50.3%)	3	2–6	213	125–298	14.87	14.77–14.97
74+ years	197,704	152,905 (32.5%)	4	2–7	226	132–326	59.32	59.10–59.54
**Sex**								
Female	920,865	339,419 (36.9%)	3	1–5	152	70–252	11.67	11.60–11.73
Male	904,381	254,128 (28.1%)	2	1–5	99	30–214	11.51	11.44–11.57
Undefined	43	12 (27.9%)	1	1–5	76	48–252	2.32	0.33–14.75
**WIMD score**^b^								
1st quintile (most deprived)	331,559	122,662 (37.0%)	3	1–6	143	55–249	12.96	12.84–13.07
2nd quintile	322,076	112,597 (35.0%)	3	1–5	137	51–244	12.74	12.62–12.85
3rd quintile	337,003	113,620 (33.7%)	3	1–5	135	51–241	12.44	12.33–12.55
4th quintile	299,113	95,158 (31.8%)	3	1–5	130	48–235	11.51	11.39–11.62
5th quintile (least deprived)	347,070	103,763 (29.9%)	3	1–5	123	46–225	10.65	10.55–10.75

UKHLS	Total	MLTCs (row %)	Median # of hospitalisation days^e^	IQR (hosp. days)	Median # GP visits^e^	IQR (GP visits)	
**Age**							
18–36 years	16,105	689 (04.1%)	3	1–6	7	3–13.5	NA
37–54 years	16,726	2184 (13.1%)	3	1–9	8	4–15	NA
55–73 years	12,308	4117 (33.5%)	5	2–14	11	6–19	NA
74+ years	4047	1886 (46.6%)	9	3–24	10	4.5–18.5	NA
**Sex**^c^							
Male	23,202	3588 (40.3%)	4	1–12	5.5	1.5–12.5	NA
Female	27,709	5308 (59.7%)	4	1–8	8.5	4–16.5	NA

IQR, Interquartile range; MLTC, Multiple long term conditions; CI, Confidence intervals.

a Lower quintiles indicate lower deprivation.

b Lower quintiles indicate higher deprivation.

c Two individuals were marked as “inconsistent sex data”.

d Number of days where any Read code was entered by a primary care provider over 10-year follow-up period.

e Self-reported GP use and hospitalisations over 5-years for which data was available during the 10-year follow-up period.

In SAIL, 1,825,289 individuals were included in the analysis, of which 593,559 (32.52%) were classed as having MLTCs, while 423,710 (23.2%) were affected by one LTC and 808,016 (44.3%) by no LTCs. For all included participants, linked GP use, hospitalisation, and mortality data were available. Higher age, male sex, and higher area deprivation were associated with higher odds for multimorbidity (Table 1).

In UKHLS, 49,186 adult individuals were included in the analysis, of which 8876 (18.1%) were classed as having MLTCs. Of those without MLTCs, 13,019 (32.3%) reported one LTC and 28,291 (67.7%) reported no LTCs. Self-reported information on number of hospitalisations and number of GP visits were recorded for 5 years during the 10-year follow-up period used in this manuscript. Hospital data were available for 13.682 (27.8%) individuals for number of admissions and from 5925 (12.0%) individuals for number of days admitted. Data on GP use was available from 23,374 (47.5%) individuals. No mortality data was available in UKHLS. Higher age, and female sex were associated with higher odds for presence of MLTCs (Table 1).

### MLTC clusters

Using LCA, we identified the following MLTC clusters for the different age groups and databases (class membership n, %)

**Table:** 

UK Biobank:		
Age 37–54 years:	Pulmonary	(n = 9683, 23%)
	Pain+	(n = 5075, 12%)
	Depression and Anxiety	(n = 5253, 13%)
	Hypertension and Cardiometabolic	(n = 16,131, 38%)
	Cancer, Thyroid disease & Rheumatoid arthritis	(n = 6007, 14%)
UK Biobank		
Age 55–73 years	Pulmonary	(n = 20,630, 17%)
	Mental health and Pain	(n = 33,168, 27%)
	Hypertension and Cardiometabolic	(n = 52,746, 43%)
	Cancer+	(n = 16,464, 13%)
SAIL:		
Age 18–36 years	Asthma+	(n = 8085, 15%)
	Pain+ (Incl. Migraine)	(n = 10,231, 19%)
	Depression+	(n = 17,698, 33%)
	Substance misuse and Mental health	(n = 11,935, 22%)
	Discordant MLTCs	(n = 5779, 11%)
SAIL:		
Age 37–54 years	Pulmonary	(n = 15,873, 11%)
	Pain+ (Incl. Migraine and Rheumatoid Arthritis)	(n = 36,929, 26%)
	Substance misuse and Mental health	(n = 18,740, 13%)
	Cardiometabolic	(n = 34,808, 25%)
	Discordant MLTCs	(n = 33,597, 24%)
SAIL:		
Age 55–73 years	Pulmonary	(n = 23,796, 10%)
	Pain+ (Incl. Migraines and Rheumatoid arthritis)	(n = 42,802, 17%)
	Mental health	(n = 40,803, 17%)
	Substance misuse and Discordant MLTCs	(n = 9001, 4%)
	Hypertension+	(n = 84,063, 34%)
	Cardiometabolic	(n = 4734, 10%)
	Cancer+	(n = 21,694, 9%)
SAIL:		
Age 74+ years	Pulmonary	(n = 15,432, 10%)
	Pain+	(n = 28,832, 19%)
	Mental health and Neurological disorders	(n = 16,136, 11%)
	Hypertension+	(n = 61,886, 41%)
	Cardiometabolic	(n = 13,221, 9%)
	Cancer+	(n = 17,399, 11%)
UKHLS:		
Age 18–36 years	Pulmonary	(n = 270, 39%)
	Depression and Asthma	(n = 251, 36%)
	Cardiometabolic	(n = 168, 24%)
UKHLS:		
Age 37–54 years	Pulmonary	(n = 805, 37%)
	Arthritis+	(n = 466, 21%)
	Diabetes and Hypertension	(n = 210, 10%)
	Cardiovascular	(n = 218, 10%)
	Cancer, Thyroid disease, and Depression	(n = 485, 22%)
UKHLS:		
Age 55–73	Pulmonary	(n = 730, 18%)
	Hypertension+	(n = 2162, 52%)
	Cardiovascular	(n = 584, 14%)
	Cancer, Thyroid disease, and Depression	(n = 641, 16%)
UKHLS:		
Age 74+	Pulmonary	(n = 380, 20%)
	Arthritis+	(n = 959, 51%)
	Cardiometabolic	(n = 547, 29%)

The derived MLTC clusters differ in the exact combination of LTCs and their respective prevalence, by following a set of naming rules (Supplement 2), similar MLTC cluster-names emerged showing similarities in between-cluster differences in each age-group and databank. MLTC clusters defined by affected systems, e.g. pulmonary, mental health, hypertension and cardiometabolic, cancer, or painful condition MLTC clusters appeared in almost all age groups across UK Biobank, SAIL and UKHLS. In SAIL, a substance misuse cluster appears across most age groups. 95% confidence intervals for all IRR in the results can be found in the respective tables and have been omitted from the main text for better readability.

### MLTC clusters and risk of adverse health outcomes and service use

**Fig. 1:**
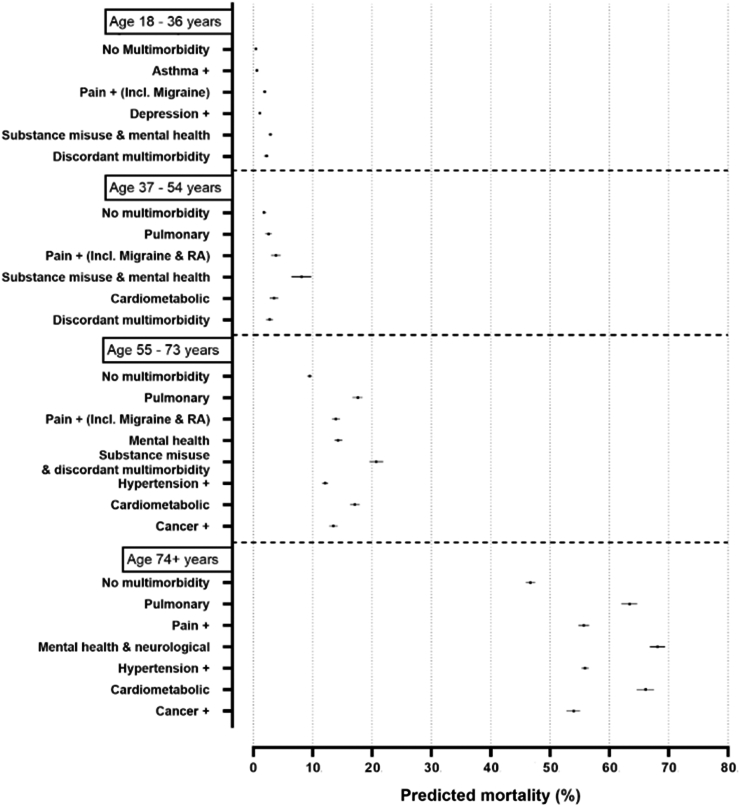
Predicted mortality rates by MLTC clusters over a 10-year follow up in SAIL. All other covariates in the model were set to their mean value in the respective age group. Error bars represent 95% confidence intervals.

**Fig. 2:**
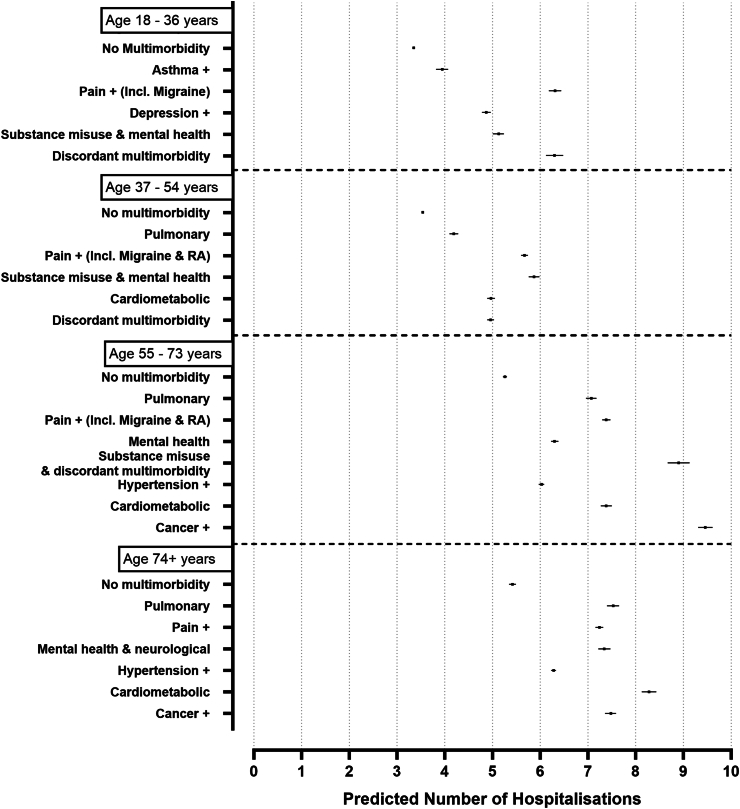
Predicted number of hospitalisations by MLTC clusters over a 10-year follow up in SAIL. All other covariates in the model were set to their mean value in the respective age group. Error bars represent 95% confidence intervals.

**Fig. 3:**
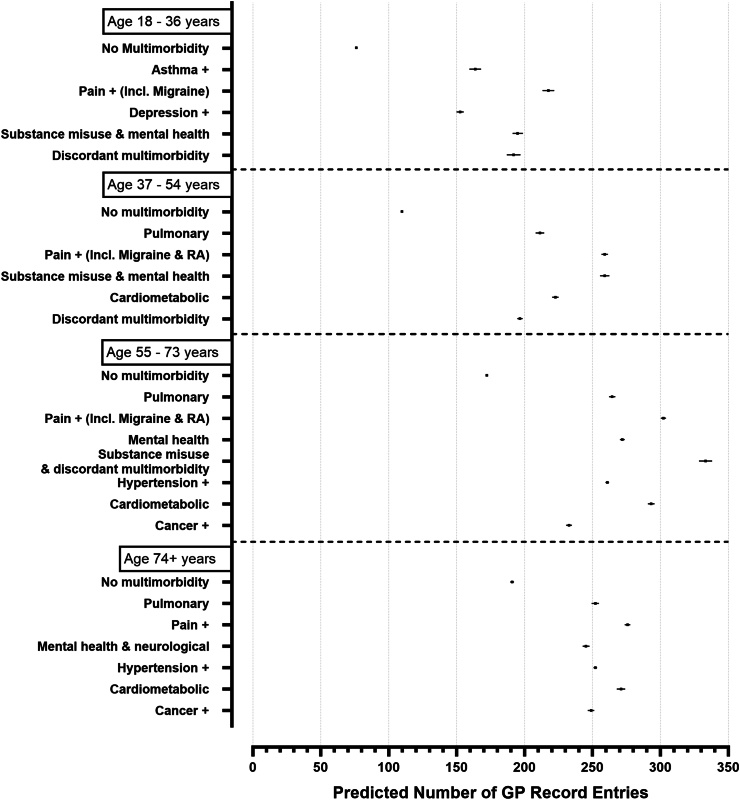
Predicted number of GP use by MLTC clusters over a 10-year follow up in SAIL. All other covariates in the model were set to their mean value in the respective age group. Error bars represent 95% confidence intervals.

The associations (IRRs with 95% confidence intervals) between MLTC clusters and risk of adverse health outcomes, adjusting for the number of LTCs, socio demographic factors, and lifestyle factors across all age groups for UK Biobank, SAIL and UKHLS are presented in Table 2 (all-cause mortality), Table 3 (hospitalisation risk), and Table 4 (GP use). Predicted number of deaths, hospitalisations, and GP use for each MLTC cluster, age-group, and database can be seen in Fig. 1, Fig. 2, Fig. 3, respectively.

**Table 2:** Regression results MLTC clusters and mortality in SAIL and UK Biobank.

SAIL				
18–36 years	IRR	p-value	95% confidence interval	
**MLTC Clusters**				
Asthma+	1.43	0.009	1.09	1.86
Pain+ (Incl. Migraine)	4.47	<0.001	3.86	5.19
Depression+	2.53	<0.001	2.15	2.98
Substance misuse & Mental Health	6.77	<0.001	6.06	7.57
Discordant MLTCs	5.17	<0.001	4.32	6.18
**LTC count**				
2 LTCs	1.46	<0.001	1.26	1.69
3 LTCs	2.86	<0.001	2.26	3.62
4 LTCs	3.90	<0.001	2.29	6.64
>4 LTCs	6.68	<0.001	4.32	6.18

SAIL					UK Biobank				
37–54 years	IRR	p-value	95% confidence interval		37–54 years	IRR	p-value	95% confidence interval	
**MLTC Clusters**					**MLTC Clusters**				
Pulmonary	1.43	<0.001	1.31	1.57	Pulmonary	1.45	0.318	0.70	2.99
Pain+ (incl. Migraine & RA)	2.11	<0.001	1.99	2.23	Pain+	1.36	0.410	0.65	2.84
Substance misuse & Mental health	4.52	<0.001	4.28	4.78	Depression & Anxiety	1.36	0.412	0.65	2.81
Cardiometabolic	1.94	<0.001	1.82	2.06	Hypertension & Cardiometabolic	1.88	0.086	0.91	3.85
Discordant MLTCs	1.52	<0.001	1.41	1.63	Cancer, Thyroid disease & RA	2.47	0.015	1.19	5.12
**LTC count**					**LTC count**				
2 LTCs	1.31	<0.001	1.24	1.38	2 LTCs	0.77	0.485	0.38	1.59
3 LTCs	1.94	<0.001	1.81	2.07	3 LTCs	0.96	0.907	0.47	1.97
4 LTCs	2.61	<0.001	2.35	2.89	4 LTCs	0.94	0.869	0.45	1.95
>4 LTCs	3.19	<0.001	2.70	3.77	>4 LTCs	1.03	0.931	0.52	2.07

SAIL					UK Biobank				
55–73 years	IRR	p-value	95% confidence interval		55–73 years	IRR	p-value	95% confidence interval	
**MLTC Clusters**					**MLTC Clusters**				
Pulmonary	1.85	<0.001	1.79	1.92	Pulmonary	1.32	0.139	0.92	1.90
Pain+ (incl. Migraine & RA)	1.47	<0.001	1.42	1.51	Mental Health & Pain	1.14	0.470	0.79	1.65
Mental health	1.51	<0.001	1.46	1.56	Hypertension & Cardiometabolic	1.48	0.034	1.03	2.13
Substance misuse & Discordant MLTCs	2.18	<0.001	2.09	2.28	Cancer+	2.03	<0.001	1.41	2.93
Hypertension+	1.27	<0.001	1.24	1.31					
Cardiometabolic	1.81	<0.001	1.75	1.87					
Cancer+	1.42	<0.001	1.37	1.48					
**LTC count**					**LTC count**				
2 LTCs	1.20	<0.001	1.17	1.22	2 LTCs	0.83	0.325	0.58	1.20
3 LTCs	1.45	<0.001	1.41	1.49	3 LTCs	0.91	0.621	0.63	1.31
4 LTCs	1.62	<0.001	1.56	1.68	4 LTCs	0.97	0.870	0.67	1.40
>4 LTCs	1.78	<0.001	1.70	1.87	>4 LTCs	1.11	0.580	0.77	1.58

SAIL				
74+ years	IRR	p-value	95% confidence interval	
**MLTC Clusters**				
Pulmonary	1.36	<0.001	1.32	1.40
Pain+	1.19	<0.001	1.17	1.22
Mental Health & Neurological disorders	1.46	<0.001	1.42	1.50
Hypertension+	1.20	<0.001	1.17	1.22
Cardiometabolic	1.41	<0.001	1.37	1.46
Cancer+	1.16	<0.001	1.12	1.19
**LTC count**				
2 LTCs	1.05	<0.001	1.04	1.07
3 LTCs	1.12	<0.001	1.10	1.14
4 LTCs	1.18	<0.001	1.15	1.21
>4 LTCs	1.23	<0.001	1.19	1.28

All models were tested with, and adjusted for, all of the following variables (incl. non-linear and interaction terms where appropriate).

Biobank: Sex, age, ethnicity, area deprivation (Townsend score), smoking status, alcohol consumption, frailty, self-rated health, body mass index (BMI), and physical activity.

SAIL: Sex, age, area deprivation (WIMD score), and smoking status.

UKHLS: Sex, age, ethnicity, BMI, alcohol consumption, physical activity, and smoking status.

**Table 3:** Regression results MLTC clusters and number of hospitalisations in SAIL, UK Biobank, and UKHLS.

SAIL				
18–36 years	IRR	p-value	95% confidence interval	
**MLTC Clusters**				
Asthma+	1.18	<0.001	1.14	1.21
Pain+ (Incl. Migraine)	1.88	<0.001	1.84	1.92
Depression+	1.45	<0.001	1.42	1.48
Substance misuse & Mental Health	1.53	<0.001	1.49	1.56
Discordant MLTCs	1.88	<0.001	1.82	1.93
**LTC count**				
2 LTCs	1.12	<0.001	1.09	1.14
3 LTCs	1.63	<0.001	1.55	1.70
4 LTCs	1.57	<0.001	1.38	1.77
>4 LTCs	2.55	<0.001	1.88	3.46

SAIL					UK Biobank				
37–54 years	IRR	p-value	95% confidence interval		37–54 years	IRR	p-value	95% confidence interval	
**MLTC Clusters**					**MLTC Clusters**				
Pulmonary	1.18	<0.001	1.15	1.21	Pulmonary	1.03	0.64	0.90	1.16
Pain+ (incl. Migraine & RA)	1.59	<0.001	1.57	1.62	Pain+	0.91	0.16	0.80	1.03
Substance misuse & Mental health	1.65	<0.001	1.62	1.68	Depression & Anxiety	1.08	0.20	0.95	1.23
Cardiometabolic	1.40	<0.001	1.37	1.42	Hypertension & Cardiometabolic	1.22	0.002	1.07	1.38
Discordant MLTCs	1.40	<0.001	1.37	1.42	Cancer, Thyroid disease & RA	1.22	0.002	1.07	1.39
**LTC count**					**LTC count**				
2 LTCs	1.14	<0.001	1.12	1.16	2 LTCs	0.98	0.87	0.87	1.12
3 LTCs	1.47	<0.001	1.43	1.50	3 LTCs	1.10	0.11	0.97	1.25
4 LTCs	1.74	<0.001	1.68	1.82	4 LTCs	1.03	0.59	0.90	1.18
>4 LTCs	2.16	<0.001	2.01	2.33	>4 LTCs	1.23	<0.001	1.10	1.39

SAIL					UK Biobank				
55–73 years	IRR	p-value	95% confidence interval		55–73 years	IRR	p-value	95% confidence interval	
**MLTC Clusters**					**MLTC Clusters**				
Pulmonary	1.34	<0.001	1.31	1.36	Pulmonary	0.97	0.65	0.86	1.09
Pain+ (incl. Migraine & RA)	1.40	<0.001	1.38	1.42	Mental Health & Pain	1.00	0.95	0.89	1.13
Mental Health	1.19	<0.001	1.18	1.21	Hypertension & Cardiometabolic	1.13	0.03	1.01	1.27
Substance misuse & Discordant MLTCs	1.69	<0.001	1.64	1.73	Cancer+	1.14	0.02	1.01	1.29
Hypertension+	1.14	<0.001	1.13	1.15					
Cardiometabolic	1.40	<0.001	1.37	1.42					
Cancer+	1.79	<0.001	1.76	1.83					
**LTC count**					**LTC count**				
2 LTCs	1.09	<0.001	1.08	1.11	2 LTCs	1.01	0.79	0.90	1.14
3 LTCs	1.30	<0.001	1.29	1.32	3 LTCs	1.08	0.18	0.96	1.22
4 LTCs	1.49	<0.001	1.46	1.52	4 LTCs	1.13	0.03	1.01	1.28
>4 LTCs	1.68	<0.001	1.63	1.74	>4 LTCs	1.21	0.001	1.07	1.35

SAIL				
74+ years	IRR	p-value	95% confidence interval	
**MLTC Clusters**				
Pulmonary	1.38	<0.001	1.35	1.41
Pain+	1.33	<0.001	1.31	1.35
Mental Health & Neurological disorders	1.35	<0.001	1.32	1.38
Hypertension+	1.15	<0.001	1.14	1.17
Cardiometabolic	1.52	<0.001	1.49	1.56
Cancer+	1.37	<0.001	1.35	1.40
**LTC count**				
2 LTCs	1.09	<0.001	1.07	1.10
3 LTCs	1.26	<0.001	1.24	1.28
4 LTCs	1.38	<0.001	1.35	1.40
>4 LTCs	1.54	<0.001	1.50	1.58

UKHLS	IRR	p-value	95% confidence interval	
18–36 years				
**MLTC Clusters**				
Pulmonary	1.21	0.609	0.30	2.02
Depression & Asthma	0.78	0.623	0.56	2.64
Cardiometabolic	2.20	0.037	1.05	4.64
LTC count (continuous due to collinearity)	1.18	0.135	0.95	1.48
37–54 years				
**MLTC Clusters (do not improve model fit, reported for convenience)**				
Pulmonary	0.76	0.231	0.48	1.20
Arthritis+	1.27	0.307	0.80	2.00
Diabetes & Hypertension	1.16	0.680	0.58	2.31
Cardiovascular	0.68	0.290	0.34	1.38
Cancer, Thyroid disease & Depression	0.81	0.397	0.49	1.33
**LTC count (values reported for best-fit model without clusters)**				
2 LTCs	1.49	0.003	1.15	1.93
3 LTCs	1.58	0.020	1.07	2.32
4+ LTCs	2.40	<0.001	1.48	3.88
55–73 years				
**MLTC Clusters (do not improve model fit, reported for convenience)**				
Pulmonary	0.97	0.854	0.69	1.36
Hypertension+	0.91	0.511	0.70	1.19
Cardiovascular	0.75	0.199	0.48	1.16
Cancer, Thyroid disease & Depression)	0.72	0.068	0.50	1.02
**LTC count (values reported for best-fit model without clusters)**				
2 LTCs	1.08	0.381	0.91	1.29
3 LTCs	1.29	0.042	1.01	1.64
4+ LTCs	1.58	0.004	1.15	2.16
74+ years				
**MLTC Clusters (do not improve model fit, reported for convenience)**				
Pulmonary	0.98	0.960	0.38	2.50
Arthritis+	1.03	0.920	0.55	1.94
Cardiometabolic	1.29	0.453	0.67	2.49
**LTC count (values reported for best-fit without clusters)**				
2 LTCs	1.52	0.042	1.02	2.27
3 LTCs	1.28	0.396	0.72	2.27
4+ LTCs	2.42	0.017	1.17	5.00

All models were tested with, and adjusted for, all the following variables (incl. non-linear and interaction terms where appropriate).

Biobank: Sex, age, ethnicity, area deprivation (Townsend score), smoking status, alcohol consumption, frailty, self-rated health, body mass index (BMI), and physical activity.

SAIL: Sex, age, area deprivation (WIMD score), and smoking status.

UKHLS: Sex, age, ethnicity, BMI, alcohol consumption, physical activity, and smoking status.

**Table 4:** Regression results MLTC clusters and number of GP use in SAIL, UK Biobank, and UKHLS.

SAIL				
18–36 years	IRR	p-value	95% confidence interval	
**MLTC Clusters**				
Asthma+	2.14	<0.001	2.08	2.19
Pain+ (Incl. Migraine)	2.91	<0.001	2.86	2.97
Depression+	2.01	<0.001	1.97	2.04
Substance misuse & Mental Health	2.67	<0.001	2.61	2.72
Discordant MLTCs	2.58	<0.001	2.52	2.65
**LTC count**				
2 LTCs	1.16	<0.001	1.14	1.19
3 LTCs	1.51	<0.001	1.44	1.58
4 LTCs	1.75	<0.001	1.55	1.97
>4 LTCs	1.75	<0.001	1.30	2.35

SAIL					UK Biobank				
37–54 years	IRR	p-value	95% confidence interval		37–54 years	IRR	p-value	95% confidence interval	
**MLTC Clusters**					**MLTC Clusters**				
Pulmonary	1.91	<0.001	1.88	1.94	Pulmonary	1.70	<0.001	1.51	1.92
Pain+ (incl. Migraine & RA)	2.39	<0.001	2.36	2.41	Pain+	1.58	<0.001	1.40	1.79
Substance misuse & Mental health	2.49	<0.001	2.46	2.52	Depression & Anxiety	1.62	<0.001	1.43	1.83
Cardiometabolic	2.04	<0.001	2.01	2.06	Hypertension & Cardiometabolic	1.81	<0.001	1.60	2.04
Discordant MLTCs	1.79	<0.001	1.77	1.81	Cancer, Thyroid disease & RA	1.73	<0.001	1.53	1.96
**LTC count**					**LTC count**				
2 LTCs	1.12	<0.001	1.11	1.13	2 LTCs	0.80	<0.001	0.60	0.90
3 LTCs	1.35	<0.001	1.33	1.37	3 LTCs	0.92	0.200	0.82	1.04
4 LTCs	1.56	<0.001	1.51	1.61	4 LTCs	0.99	0.827	0.87	1.12
>4 LTCs	1.79	<0.001	1.69	1.89	>4 LTCs	1.08	0.140	0.97	1.21

SAIL					UK Biobank				
55–73 years	IRR	p-value	95% confidence interval		55–73 years	IRR	p-value	95% confidence interval	
**MLTC Clusters**					**MLTC Clusters**				
Pulmonary	1.62	<0.001	1.61	1.64	Pulmonary	1.53	<0.001	1.38	1.69
Pain+ (incl. Migraine & RA)	1.78	<0.001	1.77	1.79	Mental Health & Pain	1.45	<0.001	1.31	1.60
Mental Health	1.60	<0.001	1.59	1.61	Hypertension & Cardiometabolic	1.57	<0.001	1.42	1.73
Substance misuse & Discordant MLTCs	2.12	<0.001	2.09	2.15	Cancer+	1.45	<0.001	1.31	1.61
Hypertension+	1.51	<0.001	1.50	1.52					
Cardiometabolic	1.78	<0.001	1.77	1.80					
Cancer+	1.40	<0.001	1.39	1.42					
**LTC count**					**LTC count**				
2 LTCs	1.10	<0.001	1.09	1.11	2 LTCs	0.83	<0.001	0.75	0.91
3 LTCs	1.26	<0.001	1.25	1.27	3 LTCs	0.92	0.085	0.83	1.01
4 LTCs	1.37	<0.001	1.36	1.39	4 LTCs	0.99	0.778	0.89	1.09
>4 LTCs	1.46	<0.001	1.44	1.49	>4 LTCs	1.06	0.218	0.97	1.17

SAIL				
74+ years	IRR	p-value	95% confidence interval	
**MLTC Clusters**				
Pulmonary	1.49	<0.001	1.47	1.50
Pain+	1.50	<0.001	1.49	1.51
Mental Health & Neurological disorders	1.57	<0.001	1.55	1.59
Hypertension+	1.36	<0.001	1.35	1.37
Cardiometabolic	1.70	<0.001	1.68	1.72
Cancer+	1.36	<0.001	1.35	1.38
**LTC count**				
2 LTCs	1.08	<0.001	1.07	1.09
3 LTCs	1.19	<0.001	1.18	1.20
4 LTCs	1.29	<0.001	1.28	1.31
>4 LTCs	1.40	<0.001	1.38	1.42

UKHLS	IRR	p-value	95% confidence interval	
18–36 years				
**MLTC Clusters (do not improve model fit, reported for convenience)**				
Pulmonary	1.60	0.040	1.02	2.51
Depression & Asthma	1.31	0.264	0.82	2.10
Cardiometabolic	1.78	0.015	1.11	2.85
**LTC count (values reported for best-fit model without clusters)**				
2 LTCs	1.48	<0.001	1.24	1.77
3 LTCs	1.29	0.165	0.90	1.85
4+ LTCs	1.49	0.378	0.61	3.63
37–54 years				
**MLTC Clusters (do not improve model fit, reported for convenience)**				
Pulmonary	1.62	<0.001	1.30	2.03
Arthritis+	1.78	<0.001	1.37	2.32
Diabetes & hypertension	1.50	0.029	1.04	2.15
Cardiovascular	1.51	0.001	1.18	1.93
Cancer, Thyroid disease & Depression	1.53	0.001	1.19	1.98
**LTC count (values reported for best-fit model without clusters)**				
2 LTCs	1.35	<0.001	1.22	1.01
3 LTCs	1.45	<0.001	1.25	1.68
4+ LTCs	1.58	<0.001	1.29	1.93
55–73 years				
**MLTC Clusters (do not improve model fit, reported for convenience)**				
Pulmonary	1.23	0.006	1.06	1.43
Hypertension+	1.17	0.008	1.04	1.31
Cardiovascular	1.13	0.156	0.95	1.35
Cancer, Thyroid disease & Depression)	1.19	0.012	1.04	1.37
**LTC count (values reported for best-fit model without clusters)**				
2 LTCs	1.21	<0.001	1.13	1.30
3 LTCs	1.23	<0.001	1.12	1.36
4+ LTCs	1.28	<0.001	1.12	1.45
74+ years				
**MLTC Clusters (do not improve model fit, reported for convenience)**				
Pulmonary	0.81	0.219	0.58	1.13
Arthritis+	1.11	0.418	0.85	1.46
Cardiometabolic	1.08	0.571	0.83	1.39
**LTC count (values reported for best-fit model without clusters)**				
2 LTCs	1.15	0.089	0.98	1.35
3 LTCs	1.14	0.209	0.93	1.4
4+ LTCs	1.33	0.029	1.03	1.71

All models were tested with, and adjusted for, any or all of the following variables (incl. non-linear and interaction terms where appropriate).

Biobank: Sex, age, ethnicity, area deprivation (Townsend score), smoking status, alcohol consumption, frailty, self-rated health, body mass index (BMI), and physical activity.

SAIL: Sex, age, area deprivation (WIMD score), and smoking status.

UKHLS: Sex, age, ethnicity, BMI, alcohol consumption, physical activity, and smoking status.

#### Age group 18–36 years (SAIL and UKHLS only)

Data for this age group were available from SAIL and UKHLS, but not UK Biobank. No data on mortality was available from UKHLS. Both MLTC clusters and LTC counts were associated with higher incidence for almost all health outcomes in this age group, excluding between MLTC clusters and number of GP use in UKHLS. Three MLTC clusters were found to have the largest adjusted strengths of associations for all the outcomes studied, when compared to the reference group without MLTCs: “*S18_Pain+ (incl. Migraine)*” (IRR mortality: 4.47; IRR hospitalisations: 1.88; IRR GP use: 2.91), “*S18_Substance misuse & Mental health*” (IRR mortality: 6.77, ; IRR hospitalisations: 1.53; IRR GP use: 2.67), and “*S18_Discordant MLTCs* (IRR mortality: 5.17; IRR hospitalisations: 1.88; IRR GP use: 2.58). Overall, these MLTC clusters had higher or comparable IRRs to that of having 4, or >4 LTCs, when compared to those without MLTCs (see Table 2, Table 3, Table 4). In UKHLS, only the cluster “U18_cardiometabolic” was associated with the number of hospitalisations (IRR: 2.20) (Table 3). MLTC clusters were not associated with higher number of GP use in UKHLS. In SAIL, the predicted probability of mortality and GP use was highest for “*S18_Pain+ (incl. Migraine)*” (Fig. 1, Fig. 3) and for “S18_Discordant MLTCs for hospitalisation events (see Fig. 2).

#### Age group 37–54 years

The highest strength of association for mortality were observed for the “*S37_Substance misuse and Mental health*” (IRR: 4.52) and the “*B37_Cancer, Thyroid disease, and Rheumatoid arthritis*” (IRR: 2.47) clusters, with the “*B37_Hypertension & Cardiometabolic*” (IRR: 1.88) cluster in UK Biobank having second-highest strength of association. The substance abuse cluster in SAIL (IRR 4.52) and the cardiometabolic cluster “*B37_Cardiometabolic*” in Biobank (IRR: 1.88) had a bigger association with mortality than having >4 LTCs in both cohorts, SAIL (IRR: 3.19) and UK Biobank (IRR: 1.03), respectively. The pain-related clusters (“*S37_Pain+ (incl. Migraine & RA)*” IRR: 2.11 & “*B37_Pain+*” IRR: 1.36), and pulmonary clusters (“*S37_Pulmonary*” IRR: 1.43 & “*B37_Pulmonary*” IRR: 1.45) likewise had higher mortality in both databanks.

The MLTC clusters with the highest IRRs for hospitalisations in this age-group in SAIL and UK Biobank were: “*S37_Subtstance misuse and Mental health*” (IRR: 1.65)), followed by the pain-related clusters (“*B37_Pain+*” (IRR: 1.59) & “*S37_Pain+ (incl. Migraine & RA)*” (IRR: 1.59)) and the cardiometabolic clusters “B37_Hypertension & Cardiometabolic” (IRR: 1.22), and “*S37_Cardiometabolic*” (IRR 1.40) as well as the pulmonary cluster: “*S37_Pulmonary*” (IRR: 1.18)), and (Table 3). The strengths of associations of these were largely comparable to the associations of having 4 or >4 LTCs, when compared to the reference group without MLTCs. Although the inclusion of MLTC clusters in the UKHLS model did not improve the model fit, the two comparable MLTC clusters *“U37_Arthritis+”* and *“U37_Diabetes & Hypertension”* in UKHLS (but not the “*U37_Asthma+*” cluster) likewise showed the highest (albeit non-significant) association with hospitalisation events, while the “*U37_pulmonary*” cluster here had the lowest IRR (Table 3).

Similar to hospitalisation event, the same MLTC clusters had the strongest association with number of GP use: “S37_Substance misuse and Mental health” (IRR:2.49) “*B37_Cancer, T. disease, and R. arthritis*” (IRR 1.73), the hypertension/cardiometabolic-related clusters “*B37_Hypertension & Cardiometabolic*” (IRR: 1.81) & “*S37_Cardiometabolic*” (IRR: 2.04) and the pulmonary-related clusters “*B37_Pulmonary*” (IRR: 1.70) & “*S37_Pulmonary*” (IRR: 1.91). The UKHLS MLTC clusters were not statistically significantly associated with GP use but show the same trend as UK Biobank and SAIL in which MLTC clusters have the biggest association when they were included in the model (see Table 4).

The absolute mortality and hospitalisation risks were highest for substance abuse and mental health MLTC clusters (see Fig. 1, Fig. 2 respectively), and the absolute risk of GP use was highest for both substance abuse and mental health and pain + MLTC clusters (see Fig. 3).

#### Age group 55–73

The MLTC clusters with the largest strengths of association on the incidence of adverse health outcomes were different across databanks in this age group.

The cluster “*S55_ Substance misuse, Mental health & Complex multimorbidity*” had the highest risk of all adverse outcomes in SAIL (mortality IRR: 2.18; hospitalisation IRR: 1.69; GP use IRR: 2.12), when compared to people without MLTCs, whereas no comparable cluster existed in the other databanks. The cancer related MLTC clusters had bigger comparative incidence rates for hospitalisations (“*S55_Cancer+*” IRR: 1.79; “*B55_Cancer+*” IRR: 14) (Table 3) than for GP use (“*S55_Cancer+*” IRR: 1.40; “*B55_Cancer+*” IRR: 1.45) (Table 4) but differed in their association with mortality (Table 2).

There was a higher association of the “*S55_Pulmonary*” (IRR 1.85) and the “*S55_cardiometabolic*” (IRR 1.81) MLTC cluster with mortality risk in SAIL, whereas for hospitalisations and GP use important clusters were “*S55_Pain (incl. Migraine & RA)”* (hospitalisation IRR: 1.40; GP use IRR: 1.78) and “*S55_Cardiometabolic*” (hospitalisation IRR: 1.40; GP use IRR: 1.78), indicating differences between drivers for mortality and disability in this age group. UKHLS MLTC clusters remained not associated with risk of hospitalisation or GP use. In SAIL, the absolute mortality risk and GP use risk was highest for substance misuse/mental health (please see Fig. 1, Fig. 3 respectively) and the absolute risk of hospitalisation was highest for the cancer MLTC cluster (see Fig. 2).

#### Age group 74+ (SAIL and UKHLS only)

Association between MLTC clusters and risk of different adverse health outcomes were found to have modest associations for the oldest age group. Higher mortality risk in this age group was mostly associated with the clusters “*S74_Mental health and Neurological disorders*” (IRR: 1.46) and “*S74_Cardiometabolic*” (IRR: 1.41), followed by “*S74_Pulmonary*” (IRR: 1.36) (see Table 2). The MLTC cluster most strongly associated with hospitalisation events was “*S74_Cardiometabolic*” (IRR: 1.52) (Table 3). UKHLS MLTC clusters were not associated with higher hospitalisation risk when LTC counts were accounted for. MLTC clusters with the highest IRR for GP use were similar to that of hospitalisations, with the most-strongly associated MLTC cluster being “*S74_Cardiometabolic*” (IRR 1.70) followed by “S74_Mental health and Neurological disorders” (IRR: 1.57) (Table 4).

### Comparing SAIL and UK Biobank MLTC clusters by predicted years of life lost

**Fig. 4:**
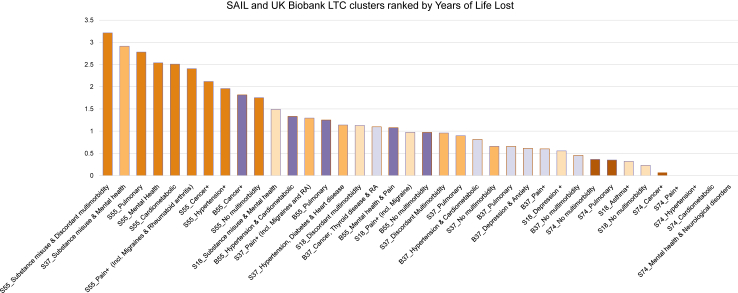
SAIL and UK Biobank LTC clusters ranked by Years of Life Lost.

The MLTC clusters with the greatest number of predicted years of life lost (YLL) were the substance abuse clusters “*S55_Substance misuse and Discordant MLTCs*”, “*S37_Substance misuse and Mental health*”, ranking 1st (i.e. greatest number of predicted life years lost), and 2nd respectively. The substance abuse cluster in the youngest age-group “*S18_Substance misuse and Mental health*” was the highest-ranking MLTC cluster in its age group, and ranked 11th overall. The MLTC clusters ranking 3rd, 4th, and 5th were “*S66_Pulmonary*”, “S55_Mental health”, and “*S55_Cardiometabolic*”, respectively (please see Fig. 4 and Supplement 4, Tables S4.1–4.3).

On average, MLTC clusters from SAIL rank higher than MLTC clusters from UK Biobank and, MLTC clusters from age groups 55–73 and 37–54 rank higher than MLTC clusters from the oldest age groups, whereas MLTC clusters from the age group 18–36 were dispersed throughout, the highest three, ranging 11th (“*S18_Substance abuse and Mental health*”), 16th (“*S18_Discordant MLTCs*”), and 19th (“*S18_Pain+ (incl. Migraines)*”), respectively.

MLTC clusters with hypertension and/or cardiometabolic-related conditions were among the highest-ranking MLTC clusters in terms of YLL in their respective age-group, with “*S55_Cardiometabolic”* ranking 5th overall and 2nd within its age group, and “B*55_Hypertension and Cardiometabolic*” ranking 12th overall and likewise 2nd within its age-group. Other Cardiometabolic clusters likewise rank between 2nd and 3rd within their age groups except for the oldest age group 74+. Cancer and pulmonary-related clusters ranked among the highest-ranking MLTC clusters for YLL in UK Biobank (cancer 1st and pulmonary 3rd rank in both age groups in UK Biobank).

An overview of SAIL and UK Biobank MLTC clusters ranked by their predicted YYL, can be seen in Fig. 4.

Tables detailing all predicted associations for mortality, number of days spent in hospital, and number of GP use with 95% confidence intervals, number of participants per MLTC cluster, prevalence of MLTC cluster, as well as rankings for all the above-mentioned outcomes and all MLTC clusters together with YLL and YLL multiplied by prevalence are available in Supplement 4 (Tables S4.1–S4.3).

### Sensitivity analysis

We performed a sex-stratified analysis of the association of MLTC clusters with outcomes in SAIL participants. Poisson and Negative binomial regressions were performed in SAIL (our largest and most representative cohort) stratified by sex of each age group and outcome. The same models were used as described in the main analysis, using the same covariates except for sex. In the youngest age group 18–36 years, females were found to have larger strengths of association with the risk of clinical outcomes/heal service use. Please see Supplement 5 (Tables S5.1–S5.6) for full results.

## Discussion

In this study, we show that MLTC clusters provide additional insights into the association of MLTCs with the outcomes: mortality, hospitalisations, and GP use. MLTC clusters have distinct associations with the odds of experiencing adverse health outcomes and increased service use, over and above the effect of LTC counts. When adjusted for LTC count and other factors, MLTC clusters showed clear associations with higher risk for mortality, hospitalisations, and GP use in the two largest cohorts, SAIL and UK Biobank. The relative impact of MLTC clusters over counts on health outcomes decreased with increasing age. However, the differences in the effect sizes between MLTC clusters remain throughout, suggesting that the MLTC clusters play an important role in determining health outcomes and resource use, which is independent of increasing numbers of LTCs.

Using LCA to group LTCs into clusters in people with MLTCs, produced largely similar results across three UK cohorts: SAIL, UK Biobank, and UKHLS. For example, MLTC clusters defined predominantly by pulmonary MLTCs appear in almost all age groups across UK Biobank, SAIL and UKHLS. Similarly, there are MLTC clusters primarily defined by mental health problems, hypertension and/or cardiometabolic disorders, cancer, painful conditions, and (in SAIL) substance misuse, in most age groups. The main discrepancies between the MLTC clusters derived from the three databanks are likely due to the different way these databanks record LTCs for their participants, with each databank using either a distinct list of LTCs or Read codes, as well as variations between self-reported vs. practitioner-recorded diagnoses and health outcomes. Similar trends to the ones we report here, can be seen in previous research clustering LTCs into groups, despite very clear differences in number and types of diseases assessed as well as how the MLTC clusters were named. In papers with relatively few and self-reported LTCs in older adults, MLTC clusters formed around Cardiometabolic/cardiovascular LTCs, Respiratory LTCs, Neuropsychiatric/cognitive LTCs and MSK/arthritis and often included a MLTC cluster that combined multiple different LTCs and could be interpreted as discordant, similar to the MLTC clusters derived for older adults in UKHLS.^25^, ^26^, ^27^, ^28^ Cardiovascular clusters in these previous papers consistently rank relatively high for mortality and hospitalisations. The only other paper that conducted clustering on different age groups found comparable clusters to our SAIL analysis showing the importance of substance abuse for mortality.^3^ Although the MLTC clusters we derived from the different databanks were largely similar, it is important to keep in mind that there are notable differences between them. For example, the two hypertension MLTC clusters in the SAIL age-groups 55–73 and 74+ years have lower relative prevalence (compared to other MLTC clusters in the same age-groups) for some of the cardiometabolic LTCs than is the case in the hypertension-related clusters of age group 37–55 in the same databank. It is therefore always important to look at the exact composition of the MLTC clusters to understand their similarities and differences. One ought to take these differences into account when comparing MLTC clusters and their relation to outcomes.

MLTC clusters in older age groups had slightly reduced relative impact on odds of mortality compared to people without MLTCs than was observed in the younger age groups, due to the low overall mortality rate in younger adults. MLTC clusters had higher strengths of association on mortality than the highest number of LTCs in several MLTC clusters in the age group 55–73 when also adjusting for counts, which shows the added value of looking at MLTC clusters together with counts. Similar variations in effect sizes were previously observed between MLTC clusters and adverse health outcomes using a large UK general practice dataset.^8^

Our YLL analysis also shows the high importance of addressing substance misuse and mental health clusters as well as cardiometabolic clusters, especially in younger and middle-aged people, as these are the MLTC clusters with some of the most years of life lost overall and in their age groups, respectively.

SAIL data on LTCs is based on Read codes entered by a physician during routinely collected data, whereas UK Biobank data relies on self-reported diagnoses of diseases, the same as UKHLS, although the latter uses a much smaller list of LTCs participants can report. Similarly, outcome measures were recorded differently. Comparisons between MLTC clusters were most valid when looking within each dataset. Nonetheless, the similarity of the MLTC clusters between databanks allows for some comparison, while keeping the limitations in mind. There were two types of potential biases which should be considered while interpreting the findings from our work: sampling and information bias. Ther was considerably heterogeneity in population characteristics of the three cohorts. SAIL was administrative data with a more representative sample to the general population while the other two research cohorts are vulnerable to selection bias. This is likely to make the population characteristics of the participants across the three cohorts quite heterogenous. For e.g. in SAIL cohort, substance misuse related clusters were identified in both 37–54 years and 55–73 years age groups, but no corresponding clusters were identified in UK Biobank in the same age groups respectively. This could be due to under representation of those with substance misuse in UK Biobank. Secondly, there is a possibility of bias due to information collection as SAIL and UK Biobank outcomes were based on linked records of death registers and health services data, while UKHLS data on hospitalisations and GP use are self-reported. Self-reported data is known to be different from record-based data due to recall bias and reporting biases. As such, the comparative potential between UKHLS and the other two databases was limited.

Years of Life Lost was calculated for all clusters and age groups based on the UK national life expectancy in 2019 with a life expectancy at birth of 81.2 years. Using life expectancy at birth may underestimate the association of MLTC clusters with mortality in older groups. We used YLL to compare clusters across different age groups and databanks. However, when focusing on older age groups, other measures that value mortality differently from YLL may be appropriate as a death close to or past life expectancy is not intrinsically less important in real life than death further away from life expectancy.

As this was a secondary data analysis, there were no formal sample size calculations. The generally smaller sample size, small number of LTCs recorded in UKHLS and resulting lower number of people with MLTCs, as well as the self-reported nature of hospitalisations and GP use, makes the UKHLS data harder to interpret and harder to compare to SAIL and UK Biobank. The number of people assigned to UKHLS clusters are relatively low when compared to the other two databanks. As such, interpretation, and comparison of strengths of associations in UKHLS need to consider the possibility of sparse data bias, especially when comparing between databanks. Likewise, the smaller number of observations in UK Biobank and UKHLS reduces power compared to SAIL and may explain the statistically non-significant association of some MLTC clusters in these databanks with outcomes better than an actual lack of association. During the regression modelling, UKHLS MLTC clusters show little to no association with health outcomes and LTC counts alone generally lead to better model parsimony than including MLTC clusters in all but the youngest age group in UKHLS. It is nonetheless encouraging to see, that somewhat similar MLTC clusters emerge from the LCA in UKHLS and the other two databanks. Naming of MLTC clusters in LCA is a complex issue and the name of MLTC clusters can greatly influence a reader's interpretation of the results presented. When interpreting the results for individual MLTC clusters and/or similarly named MLTC clusters, it is important to look in detail into the exact composition of each MLTC cluster.^29^ The main purpose of our statistical modelling was to describe the association of clusters with the outcomes adjusted for covariates. The relationship between covariables such as deprivation, frailty, and behavioural factors and clusters is likely very complex as diverse underlying LTCs are influenced in different ways and magnitudes by different confounders. We therefore opted to fit the models based on statistical methods alone, despite the risk of potentially excluding clinically important confounders when selection is based on such an approach. The in-depth analysis and interpretation of the varied interrelationships between clusters, covariates, and outcomes warrants further analysis and causative modelling. The other limitation of this study is the possibility of residual and unmeasured confounding, which is a common limitation of observational studies, especially with measurement errors in capturing smoking and alcohol consumption levels. Of note, the information on smoking and alcohol consumption and physical activity levels in UKHLS were collected in wave 2 which was on average 12 months after the baseline, this is based on the assumption that the differences in social-behavioural factors are likely to be a lot smaller over a 1–2-year period (between the first wave from Jan 2009 to March 2011 and second wave from Jan 2010 to March 2012) than they will be over the entire 10 year follow-up of the study, and that the margin of error between the first and second wave is therefore acceptable.

This research shows that the combinations of LTCs in MLTC clusters have a distinct association with health outcomes and resource use, in addition to the effect of number of LTCs, particularly in younger age groups. Our work highlights the heterogenous nature of people living with MLTCs and the importance of developing tailored responses to people with different MLTCs particularly those with the highest risk of adverse outcomes such as “*Pain+*” cluster in the age-group 18–36 years and the “*Hypertension, Diabetes & Heart disease*” cluster in the age-group 37–54 years. The findings from this study will be used to inform patient selection within the PERFORM trial which will design and test personalised exercise-based interventions for people with MLTCs that have a known benefit from exercise.^10^ Improved risk stratification of people with MLTCs, for example, through taking greater account of MLTC clusters in addition to LTC counts and other sociodemographic factors, may have important implications for practice (in relation to secondary prevention), policy (with distribution of resources), and research (intervention development and targeting), for people living with MLTCs.

## Contributors

The study was conceptualised by BDJ, EM, FSM, LS, RST, SJK, SJS, SA. BDJ, FSM, JL, LS, SJK, SA and EM contributed to the methodology. Data analysis was carried out by BDJ, JL, LS, SJK, SA and EM. SJK and LS have verified the data analysis. Data was accessed by BDJ, LS, SJK and SA. Data curation was by BDJ, BIN, DM, FSM, PH and RST. SJK wrote the first draft of the paper. All authors reviewed and edited the manuscript. Data visualisation was by LS and SJK. Supervision was provided by BDJ, FSM, EM, RST and SJS. Project administration was by RST, FSM, SJS, BDJ and ZA. Funding was acquired by BDJ, EM, FSM, KJ, NG, PD, PO, RAE, RST, SAS, SB, SD, SJS, TI, CJG.

## Data sharing statements

The data that support the findings of this study are available from SAIL, UK Biobank and UKHLS project site, subject to successful registration and application process. We have shared the relevant links for applying for data access below.

SAIL: https://saildatabank.com/data/apply-to-work-with-the-data/.

UK Biobank: https://www.ukbiobank.ac.uk/enable-your-research/apply-for-access.

UKHLS: https://www.understandingsociety.ac.uk/documentation/access-data/.

## Declaration of interests

SJS is Clinical Lead for National Respiratory Audit Programme–Pulmonary Rehabilitation and also a National Institute for Health Research (NIHR) Senior Investigator. This work was supported by the NIHR Leicester Biomedical Research Centre (BRC). KJ declares funding from NIHR Applied Research Collaboration West Midlands and Sub-committee chair for NIHR Programme Grants for Applied Health Research (payment to institution). BIN declares receiving funding from NIHR, vs. Arthritis, NIHR-EPSRC, Glasgow Knowledge Exchange Fund and for external PhD examination. RAE declares receipt of speaker fees (Boeringher June 2021; Moderna April 2023) and ERS Group 01.02 Pulmonary Rehabilitation and Chronic Care Secretary (unpaid), and ATS Pulmonary Rehabilitation Assembly Chair (unpaid). SD declares NIHR Applied Research Collaboration: South West Peninsula (PenARC; payment to institution), receipt of the following NIHR grants (payment to institution): NIHR151938; NIHR204099; RP-PG-0514-20,002; NIHR201038; NIHR201070; NIHR200428, receipt of grants (payment to institution): Gillings Family foundation (ID 943008); The Stroke Association (ID 901902); NIHR School for Primary Care Research—Exeter internal fund (ID 856766); Academic Health Science Network South West (ID 1355693), receipt of textbook royalties (John Wiley & Sons), support for meeting attendance from NIHR (p-PG-0514- 20,002) and Health Research Council New Zealand (21/826; 18/254), and membership of NIHR Programme Grant for Applied Research funding panel committee and The Stroke Association research funding panel. SJK declares receipt of conference funding from School of Health and Wellbeing, University of Glasgow. SAS declares presidency of the UK Society of Behavioural Medicine, membership of HTA Clinical Evaluations and Trials Committee (2016–2020), membership of Commissioning Panel for the National Institute of Health Research (NIHR) Policy Research Programme (2019–2022), and membership of Chief Scientist Office HIPS committee (2018–2023). FSM declares grants from NIHR during the conduct of the study; grants from Wellcome, grants from Innovate UK, grants from Innovative Medicines Initiative (IMI2), grants from UKRI, grants from EPSRC, grants from MRC, grants from Chief Scientist office Scotland, outside the submitted work; and MRC CARP Panel membership. Dr Greaves reports grants from National Institute for Health Research (NIHR) during the conduct of the study.
