# Supplementary material for: Association of latent class analysis-derived multimorbidity clusters with adverse health outcomes in patients with multiple long-term conditions: comparative results across three UK cohorts
Source: eClinicalMedicine. 2024 Jun 28;74:102703. doi: 10.1016/j.eclinm.2024.102703 (PMC11261399; doi:10.1016/j.eclinm.2024.102703)
Supplement: Supplement Material [file mmc1.pdf]

Supplement 1 LTCs and variables available in the different databases

Table S1.1: Long term conditions (LTCs) in UK Biobank, SAIL, and UKHLS used for clustering and statistical modelling

Biobank (43)	SAIL (43)	UKHLS (13)
Alcohol problems	Alcohol problems	
Anorexia nervosa or bulimia	Anorexia nervosa or bulimia	
Anxiety	Anxiety	
Asthma	Asthma	Asthma
Atrial fibrillation	Atrial fibrillation	
Bronchiectasis	Bronchiectasis	
Cancer	Cancer	Cancer or malignancy
Chronic fatigue syndrome	Chronic fatigue syndrome	
Chronic kidney disease	Chronic kidney disease	
Chronic liver disease	Chronic liver disease	Any liver condition
Chronic sinusitis	Chronic sinusitis	
Chronic obstructive pulmonary disease	Chronic obstructive pulmonary disease	COPD[2]
Coronary heart disease	Coronary heart disease	Coronary heart disease[1]
Dementia	Dementia	
Depression	Depression	Clinical depression
Diabetes mellitus	Diabetes mellitus	Diabetes
Diverticular disease	Diverticular disease	
Endometriosis	Endometriosis	
Epilepsy	Epilepsy	Epilepsy
Glaucoma	Glaucoma	
Heart failure	Heart failure	Congestive heart failure
Hypertension	Hypertension	Hypertension
Inflammatory bowel disease	Inflammatory bowel disease	
Irritable bowel syndrome	Irritable bowel syndrome	
Meniere’s disease	Meniere’s disease	
Migraine	Migraine	
Multiple sclerosis	Multiple sclerosis	
Osteoporosis	Osteoporosis	
Psychoactive substance misuse	Psychoactive substance misuse	
Painful condition	Painful condition	
Parkinson’s disease	Parkinson’s disease	
Peripheral vascular disease	Peripheral vascular disease	
Pernicious anaemia	Pernicious anaemia	
Polycystic ovarian syndrome	Polycystic ovarian syndrome	
Prostate disorders	Prostate disorders	
Psoriasis or eczema	Psoriasis or eczema	
Rheumatoid Arthritis / Connective tissue disease / Inflammatory arthropathies	Rheumatoid Arthritis / Connective tissue disease / Inflammatory arthropathies	Arthritis

Schizophrenia or bipolar affective disorder	Schizophrenia or bipolar affective disorder	
Stroke or transient ischaemic attack	Stroke or transient ischaemic attack	Stroke
Thyroid disease	Thyroid disease	Thyroid disease[3]
Treated constipation	Treated constipation	
Treated dyspepsia	Treated dyspepsia	
Viral hepatitis	Viral hepatitis	

[1] Collapsed into single ‘coronary heart disease’ together with “Angina” and “Heart attack or myocardial infarction” in line with list of 43 LTCs. [2] Collapsed into single ‘COPD’ together with “Emphysema” and “Chronic bronchitis” in line with list of 43 LTCs. [3] “Hyperthyroidism” and “hypothyroidism” collapsed into single ‘thyroid disease’ outcome in line with list of 43 LTCs.

Table S1.2: Variables available in UK Biobank, SAIL, and UKHLS used for statistical modelling

Measure	UK Biobank	SAIL	UKHLS
LTC count	Based on presence/absence of 43 LTCs<br>1: 01- LTCs;<br>2: 2 LTCs;<br>3: 3 LTCs;<br>4: 4 LTCs;<br>5: >4 LTCs	Based on presence/absence of 43 LTCs<br>1: 01- LTCs;<br>2: 2 LTCs;<br>3: 3 LTCs;<br>4: 4 LTCs;<br>5: >4 LTCs	Based on presence/absence of 13 LTCs<br>1: 01- LTCs;<br>2: 2 LTCs;<br>3: 3 LTCs;<br>4: >3 LTCs;
Mortality	Linked death register data	Linked death register data	N/A
Hospitalisations	Linked hospital data. Number of unique admissions for any cause. Unique admissions were counted as admissions that were a minimum of 24h apart	Linked hospital data. Number of unique admissions for any cause. Unique admissions were counted as admissions that were a minimum of 24h apart	Number of self-reported hospital admissions
GP use	Number of unique days on which any read codes were entered by a primary care provider	Number of unique days on which any read codes were entered by a primary care provider	Number of self-reported GP encounters
Days spent in hospital	Linked hospital data. Number of unique admissions for any cause. Total number of days spent in hospital	Linked hospital data. Number of unique admissions for any cause. Total number of days spent in hospital	Self-reported number of days spent in hospital
Sex	1: Female<br>2: Male	1: Female<br>2: Male<br>3: Undefined	1: Male<br>2: Female
Age	37-54 years<br>55-73 years	18-36 years<br>37-54 years<br>55-73 years<br>>73 years	18-36 years<br>37-54 years<br>55-73 years<br>>73 years
Deprivation	Townsend deprivation score	Welsh Index of Multiple Deprivation (WIMD score)	N/A
Ethnicity	0: White<br>1: Asian or Asian British<br>2: Black or Black British<br>3: Chinese<br>4: Mixed<br>5 Other	N/A	1: White<br>2: Mixed<br>3: Asian or Asian British<br>4. Chinese<br>5: Black or Black British<br>6: Other
Smoking	1: Never<br>2: Previously<br>3: Current	N/A	0: Never<br>1: Previous<br>2: Current

			(Collected at Wave 2)
Alcohol frequency	0: Never<br>1: on special occasions only<br>2: 1-3 times per month<br>3: 1-2 times per week<br>4: 3-4 times per week<br>5: (Almost daily)	N/A	1: Never/ up to 2 times per year<br>2: Up to 1-2 times per month<br>3: 1-4 times per week<br>4: Daily or almost daily<br>(Collected at Wave 2)
BMI	Continuous	N/A	Continuous
Physical activity	1: high<br>2: medium<br>3: low<br>4: none	N/A	Continuous.<br>Number of days walked at least 30 mins in past 4 weeks.<br>(Collected at Wave 2)
Frailty	Frailty index: fraction based on 5 domains (yes/no): grip, energy, slowness, weight loss, new low physical activity<br>0: frailty in none of the domains<br>0.2: frailty in 1 domain<br>0.4: frailty in 2 domains<br>0.6: frailty in 3 domains<br>0.8: frailty in 4 domains<br>1.0: frailty in 5 domains	N/A	N/A
Self-rated health	1: Excellent<br>2: Fair<br>3: Good<br>4: Poor	N/A	N/A

Supplement 2: Cluster composition and naming:

Considerations:

- Rarer conditions might not have a high prevalence but could be driver for differences between clusters.
- In some age groups, very common conditions appear with high prevalence in almost all clusters but don't drive difference between clusters (e.g. depression in younger age groups, hypertension in older age groups).
- LCA finds clusters that are unique and differ from each other by a latent, unobserved effect → some conditions appear predominantly in one of the clusters and not others, making between-clusters pattern important.

Applied convention for naming:

- Considered between cluster differences first, include LTCs that had substantially higher prevalence in one cluster compared to all others in name (rule of thumb approximately twice than next highest cluster).
- Labels include LTCs with 100% within-cluster prevalence and/or the highest between-cluster prevalence.
- Labelled according to affected systems where LTCs with highest within and between prevalence affect similar physiology and body systems (e.g. Pulmonary, cardiovascular, etc.)
- Used '+' when cluster named after single condition with highest within and/or between cluster prevalence.
- Where a single LTC dominates the cluster , a + was added to the name to indicate that a collection of other LTCs are also in this cluster albeit without other obvious between-cluster differences that warrant naming.
- Clusters with several LTCs with high within and between cluster prevalence which affect multiple systems and without one single dominant LTC in the cluster, were labelled Discordant.

Note:

The clusters have been named for convenience in discussion and write-up. Clusters are complex and no naming convention will comprehensively describe a cluster. Readers are advised to investigate the prevalence of LTCs in detail when interpreting the data and results.

UK Biobank 37-54 years					
Cluster name	Pulmonary	Pain+	Depression & Anxiety	Hypertension & Cardiometabolic	Cancer, Thyroid disease & RA
#	1	2	3	4	5
Predicted class proportion	0.23	0.12	0.13	0.38	0.14
Estimated class population, based on modal probability (n)	9683	5075	5253	16131	6007
Long term condition	Conditional item response probabilities				
Alcohol	0.00	0.00	0.02	0.01	0.01
Anorexia/Bulimia	0.00	0.00	0.02	0.00	0.01
Anxiety	0.04	0.05	0.19	0.03	0.09
Asthma	**1.00**	0.03	0.17	**0.21**	0.00
Atrial Fibrillation	0.00	0.01	0.00	0.01	0.01
Bronchiectasis	0.01	0.00	0.00	0.00	0.00
Cancer	0.09	0.09	0.08	0.08	**0.22**
Chronic Fatigue Syndrome	0.01	0.02	0.03	0.01	0.03
Chronic Kidney Disease	0.00	0.00	0.00	0.02	0.01
Chronic Liver Disease	0.00	0.00	0.00	0.00	0.01
Chronic Sinusitis	0.02	0.03	0.01	0.01	0.03
COPD	0.05	0.02	0.02	0.02	0.03
Coronary Heart Disease	0.02	0.03	0.02	0.10	0.05
Dementia	0.00	0.00	0.00	0.00	0.00
Depression	0.10	0.09	**1.00**	0.11	0.04
Diabetes	0.04	0.04	0.04	**0.22**	0.09
Diverticular disease	0.01	0.01	0.01	0.01	0.03
Endometriosis	0.03	0.04	0.03	0.01	0.07
Epilepsy	0.02	0.02	0.02	0.02	0.05
Glaucoma	0.01	0.01	0.01	0.01	0.02
Heart Failure	0.00	0.00	0.00	0.01	0.01
Hypertension	0.15	0.17	0.19	**1.00**	0.08
Inflammatory Bowel Disease	0.02	0.02	0.01	0.01	0.05
Irritable Bowel Syndrome	0.06	0.10	0..09	0.03	0.14
Meniere's disease	0.00	0.01	0.00	0.00	0.01
Migraine	0.08	0.14	0.10	0.04	0.19
Multiple Sclerosis	0.01	0.01	0.01	0.01	0.02
Osteoporosis	0.01	0.02	0.01	0.01	0.04
Psychoactive substance misuse	0.00	0.00	0.00	0.00	0.00
Painful condition	**0.24**	**1.00**	**0.25**	**0.22**	0.00
Parkinson's disease	0.00	0.00	0.00	0.00	0.00
Peripheral vascular disease	0.00	0.01	0.00	0.00	0.01
Pernicious Anaemia	0.01	0.01	0.00	0.00	0.02
Polycystic Ovarian Syndrome	0.01	0.01	0.01	0.00	0.02
Prostate disorders	0.01	0.01	0.00	0.01	0.01
Psoriasis or Eczema	0.19	0.11	0.08	0.05	0.18
Rheumatoid arthritis/ Inflammatory arthropathies/ Connective tissue disease	0.04	0.04	0.02	0.03	0.12
Schizophrenia or bipolar disorder	0.01	0.01	0.02	0.01	0.03
Stroke or TIA	0.01	0.01	0.02	0.04	0.04
Thyroid disease	0.10	0.11	0.10	0.09	**0.24**
Treated constipation	0.00	0.00	0.00	0.00	0.00
Treated dyspepsia	0.14	**0.21**	0.15	0.14	**0.24**
Viral Hepatitis	0.01	0.01	0.01	0.00	0.01

Legend: **Bold red: >=50% prevalence within Cluster; Bold black: 20-59% prevalence within cluster**;

Green background: substantially higher prevalence between clusters (ca double or more);

Yellow background: substantially lower prevalence between clusters (no more than 1/4th of next highest cluster);

Grey: highest between-cluster prevalences, but very low prevalence overall. Mostly ignoring these between cluster differences for naming unless it's a unique appearance of an LTC in one cluster.

Naming Rational for Biobank 37-54 years:

Pulmonary:
Highest prevalence of Asthma between and within cluster (100%)COPD and Bronchiectasis highest prevalence in this cluster compared to all other clusters (highest between cluster prevalence). Bronchiectasis only grouped into this cluster. There's a clear clustering of pulmonary disorders. Other conditions of similar or substantially lower prevalence than in other clusters.

Pain+:
Painful condition has highest prevalence within and between clusters. Not much else of note. Dyspepsia next highest within cluster but not substantially higher between clusters. Other conditions of similar or substantially lower prevalence than in other clusters.

Depression and Anxiety:
Depression highest level within and between clusters. Anxiety highest level between clusters. Other conditions of similar or substantially lower prevalence than in other clusters.

Hypertension and Cardiometabolic:
Hypertension 100% = highest level within and between clusters. But also highest level of diabetes between clusters with prevalence substantially higher than all other clusters and highest level of heart disease between clusters. Diabetes and Coronary heart disease are both associated with hypertension. Other conditions of similar or substantially lower prevalence than in other clusters.

Cancer, Thyroid disease, and Rheumatoid arthritis:
Highest between cluster level of Cancer (approximately 3x higher), thyroid disease (2x higher) and rheumatoid arthritis (3x higher).. Of note is the complete absence of "painful conditions". 6% of cancers for females in this age group are thyroid cancers (cancer research UK). Only cluster with liver disease (<1%) Cancer, thyroid disease, and dyspepsia highest within cluster prevalence. Highest prevalence (albeit with less than 3% difference) also for Endometriosis, Epilepsy, IBD, Osteoporosis. Cluster perhaps reflects discordant multimorbidity.

UK Biobank 55-73 years				
Cluster name	Pulmonary	Mental health & Pain	Hypertension & Cardiometabolic	Cancer+
#	1	2	3	4
Predicted class proportion	0.17	0.27	0.43	0.13
Estimated class population, based on modal probability (n)	20630.00	33168.00	52746.00	16464.00
	Conditional item response probabilities			
Alcohol	0.00	0.01	0.00	0.00
Anorexia/Bulimia	0.00	0.00	0.00	0.00
Anxiety	0.02	0.07	0.02	0.02
Asthma	1.00	0.08	0.12	0.08
Atrial Fibrillation	0.01	0.02	0.03	0.01
Bronchiectasis	0.02	0.01	0.00	0.00
Cancer	0.11	0.04	0.06	1.00
Chronic Fatigue Syndrome	0.01	0.02	0.00	0.01
Chronic Kidney Disease	0.00	0.00	0.01	0.00
Chronic Liver Disease	0.00	0.00	0.01	0.00
Chronic Sinusitis	0.02	0.02	0.01	0.01
COPD	0.12	0.04	0.04	0.03
Coronary Heart Disease	0.06	0.07	0.23	0.06
Dementia	0.00	0.00	0.00	0.00
Depression	0.09	0.18	0.06	0.07
Diabetes	0.05	0.03	0.28	0.05
Diverticular disease	0.03	0.05	0.02	0.02
Endometriosis	0.01	0.03	0.00	0.01
Epilepsy	0.01	0.02	0.01	0.01
Glaucoma	0.02	0.03	0.03	0.03
Heart Failure	0.00	0.00	0.01	0.00
Hypertension	0.36	0.34	0.88	0.44
Inflammatory Bowel Disease	0.02	0.02	0.01	0.01
Irritable Bowel Syndrome	0.04	0.10	0.01	0.02
Meniere's disease	0.00	0.01	0.00	0.01
Migraine	0.03	0.11	0.01	0.04
Multiple Sclerosis	0.00	0.01	0.00	0.00
Osteoporosis	0.05	0.08	0.02	0.06
Psychoactive substance misuse	0.00	0.00	0.00	0.00
Painful condition	0.29	0.51	0.33	0.27
Parkinson's disease	0.00	0.01	0.00	0.00
Peripheral vascular disease	0.00	0.01	0.01	0.00
Pernicious Anaemia	0.01	0.01	0.00	0.00
Polycystic Ovarian Syndrome	0.00	0.00	0.00	0.00
Prostate disorders	0.03	0.05	0.05	0.02
Psoriasis or Eczema	0.10	0.09	0.04	0.03
Rheumatoid arthritis/ Inflammatory arthropathies/ Connective tissue disease	0.04	0.08	0.04	0.04
Schizophrenia or bipolar disorder	0.00	0.01	0.01	0.00
Stroke or TIA	0.02	0.03	0.08	0.03
Thyroid disease	0.10	0.18	0.11	0.11
Treated constipation	0.00	0.00	0.00	0.00
Treated dyspepsia	0.16	0.29	0.14	0.13
Viral Hepatitis	0.00	0.01	0.00	0.00

Legend: **Bold red: >=50% prevalence within Cluster**; **Bold black: 20-59% prevalence within cluster**;

Green background: substantially higher prevalence between clusters (ca double or more);

Yellow background: substantially lower prevalence between clusters (no more than 1/4th of next highest cluster);

Grey: highest between-cluster prevalences, but very low prevalence overall. Mostly ignoring these between cluster differences for naming unless it's a unique appearance of an LTC in one cluster.

Naming Rational for Biobank 55-73 years:

Pulmonary:
Asthma highest level within and between clusters (100%). COPD highest between clusters (3X higher than the next highest). Also highest level of Bronchiectasis (but it's below 3% total prevalence). Painful conditions and Hypertension are on par with other clusters in the age group. Other conditions of similar or substantially lower prevalence than in other clusters.

Mental health and Pain:
This cluster has the highest levels of Depression and Anxiety of all clusters. Pain >50% within cluster, but not substantially higher than for other clusters (pain is ubiquitous in the age group). However, other conditions that are also associated with pain are at their highest in-between cluster prevalence (Migraines, endometriosis, IBS, RA).

Note: comparing the clusters between SAIL and Biobank, it seems that this cluster is very similar to the substance abuse cluster in SAIL for the same age group. Biobank has an absence of reported drug abuse (reporting bias). This cluster does have the highest in-between level of Alcohol misuse as well as other diseases that are also in the substance abuse cluster in the same age group in SAIL and would seem otherwise out of place looking just at this cluster. incl. digestive issues. However, these observations do not influence naming, or interpretation as they are speculative.

Hypertension and Cardiometabolic:
Hypertension is highest within and between clusters, Diabetes is highest between clusters (>5x higher prevalence than any other cluster), heart disease highest prevalence between clusters (>3X more than next highest). Makes sense that heart disease would join hypertension & diabetes since hypertension & heart disease correlate. Painful conditions are on par with other clusters. Other conditions of similar or substantially lower prevalence than in other clusters.

Cancer+:
Cancer has the highest within and between clusters. Hypertension is quite high but the prevalence in this cluster is on par with other clusters and Hypertension is ubiquitous in the age group. Similar with painful conditions. No other LTC stick out as higher/lower between clusters.

SAIL 18-36 years					
Suggested name	Asthma+	Pain+ (Incl. migraine)	Depression+	Substance misuse & Mental health	Discordant
#	1	2	3	4	5
Predicted class membership	0.15	0.19	0.33	0.22	0.11
Estimated class population, based on modal probability (n)	8085.00	10321.00	17698.00	11935.00	5779.00
Long term condition	Conditional item response probabilities				
Alcohol	0.02	0.02	0.06	**0.30**	0.02
Anorexia/Bulimia	0.01	0.01	0.06	0.01	0.02
Anxiety	0.01	0.07	0.03	0.15	0.02
Asthma	**1.00**	0.17	0.00	0.09	0.16
Atrial Fibrillation	0.00	0.00	0.00	0.00	0.01
Bronchiectasis	0.00	0.00	0.00	0.00	0.01
Cancer	0.03	0.03	0.09	0.02	0.14
Chronic Fatigue Syndrome	0.01	0.01	0.03	0.00	0.02
Chronic Kidney Disease	0.00	0.01	0.02	0.00	0.09
Chronic Liver Disease	0.00	0.00	0.00	0.00	0.01
Chronic Sinusitis	0.04	0.02	0.06	0.01	0.07
COPD	0.07	0.02	0.05	0.03	0.08
Coronary Heart Disease	0.00	0.00	0.00	0.00	0.00
Dementia	0.00	0.00	0.00	0.00	0.00
Depression	**0.73**	**0.64**	**1.00**	**0.85**	0.07
Diabetes	0.05	0.05	0.11	0.02	**0.24**
Diverticular disease	0.00	0.00	0.00	0.00	0.01
Endometriosis	0.02	0.04	0.05	0.00	0.05
Epilepsy	0.02	0.04	0.03	0.03	0.11
Glaucoma	0.00	0.00	0.00	0.00	0.01
Heart Failure	0.00	0.00	0.00	0.00	0.01
Hypertension	0.03	0.04	0.07	0.01	**0.21**
Inflammatory Bowel Disease	0.01	0.02	0.02	0.00	0.06
Irritable Bowel Syndrome	0.01	0.03	0.02	0.00	0.03
Meniere’s disease	0.00	0.00	0.00	0.00	0.00
Migraine	0.00	**0.22**	0.00	0.00	0.00
Multiple Sclerosis	0.00	0.01	0.01	0.00	0.01
Osteoporosis	0.00	0.00	0.00	0.00	0.02
Psychoactive substance misuse	0.02	0.04	0.01	**0.74**	0.01
Painful condition	0.04	**1.00**	0.05	0.08	0.07
Parkinson’s disease	0.00	0.00	0.00	0.00	0.00
Peripheral vascular disease	0.01	0.02	0.04	0.01	0.06
Pernicious Anaemia	0.00	0.00	0.01	0.00	0.01
Polycystic Ovarian Syndrome	0.04	0.04	0.14	0.01	0.16
Prostate disorders	0.00	0.00	0.00	0.00	0.01
Psoriasis or Eczema	0.01	0.01	0.02	0.01	0.02
Rheumatoid arthritis/ Inflammatory arthropathies/ Connective tissue disease	0.00	0.02	0.01	0.00	0.03
Schizophrenia or bipolar disorder	0.01	0.01	0.03	0.11	0.02
Stroke or TIA	0.00	0.01	0.01	0.00	0.02
Thyroid disease	0.03	0.03	0.09	0.01	0.17
Treated constipation	0.01	0.07	0.03	0.01	0.08
Treated dyspepsia	0.11	**0.19**	0.13	0.08	**0.21**
Viral Hepatitis	0.00	0.00	0.00	0.00	0.00

Legend: **Bold red: >=50% prevalence within Cluster**; **Bold black: 20-59% prevalence within cluster**;

Green background: substantially higher prevalence between clusters (ca double or more);

Yellow background: substantially lower prevalence between clusters (no more than 1/4th of next highest cluster);

Grey: highest between-cluster prevalences, but very low prevalence overall. Mostly ignoring these between cluster differences for naming unless it’s a unique appearance of an LTC in one cluster.

Naming Rational for SAIL 18 - 36 years:

Asthma+:
Asthma highest prevalence within and between clusters. Depression above 50% but on par with all but one cluster. COPD higher than most clusters but not the highest, Bronchiectasis in another cluster → likely Asthma specific, not "pulmonary". Other conditions of similar or substantially lower prevalence than in other clusters.

Pain+ (Incl. Migraine):
Painful conditions have highest prevalence between and within cluster. Migraine is highest between clusters and also a pain disorder. Depression high but very similar to other clusters. Dyspepsia relatively high, but not exclusively in this cluster and possible correlation with analgesic medication. Other conditions of similar or substantially lower prevalence than in other clusters.

Depression+:
Depression is the only condition with a high within-cluster prevalence, and the highest between cluster prevalence. PCOS shows up, but similar prevalence to another cluster. Anorexia/bulimia is also highest in this cluster. Argument for calling it mental health, due to eating disorder, but very low prevalence and different profile to other mental health clusters suggests not to. Other conditions of similar or substantially lower prevalence than in other clusters.

Substance misuse and Mental health:
Highest level of psychoactive substance and alcohol misuse between clusters (psychoactive highest prevalence within cluster as well). Anxiety highest prevalence between clusters, same with schizophrenia. Depression second highest between clusters. Other conditions of similar or substantially lower prevalence than in other clusters.

Discordant multimorbidity:
This cluster has the highest between-cluster prevalence of Cancer, Chronic Kidney disease, Diabetes, Epilepsy, Hypertension, IBD, and Thyroid disease. Also is the only cluster where some of the much lower prevalence LTCs (for this age group) appear incl. Atrial fibrillation, Bronchiectasis, Chronic liver disease, Diverticulitis, Glaucoma, and Heart failure. Substantially lower prevalence of depression than in other clusters in the age group. No single or combined LTCs are particularly prominent in identifying the cluster, so represents mixed/discordant multimorbidity.

Sail 37-54 years					
0Cluster name	Pulmonary	Pain+ (incl. Migraine & RA)	Substance misuse & Mental health	Cardiometabolic	Discordant
#	1	2	3	4	5
Predicted class membership	0.11	0.26	0.13	0.25	0.24
Estimated class population (n)	15873.00	36929.00	18740.00	34808.00	33597.00
Long term condition	Conditional item response probabilities				
Alcohol	0.02	0.01	0.33	0.03	0.01
Anorexia/Bulimia	0.00	0.00	0.01	0.00	0.01
Anxiety	0.01	0.08	0.21	0.00	0.01
Asthma	0.88	0.15	0.12	0.09	0.02
Atrial Fibrillation	0.00	0.00	0.01	0.03	0.01
Bronchiectasis	0.02	0.00	0.00	0.00	0.00
Cancer	0.06	0.06	0.03	0.06	0.19
Chronic Fatigue Syndrome	0.01	0.01	0.00	0.00	0.03
Chronic Kidney Disease	0.01	0.02	0.01	0.13	0.02
Chronic Liver Disease	0.00	0.00	0.02	0.02	0.00
Chronic Sinusitis	0.06	0.04	0.02	0.02	0.10
COPD	0.23	0.05	0.07	0.04	0.05
Coronary Heart Disease	0.01	0.01	0.01	0.05	0.00
Dementia	0.00	0.00	0.00	0.00	0.00
Depression	0.52	0.59	0.86	0.39	0.73
Diabetes	0.10	0.10	0.08	0.38	0.11
Diverticular disease	0.02	0.02	*0.01*	*0.03*	0.04
Endometriosis	0.02	0.02	0.01	0.01	0.07
Epilepsy	0.02	0.03	0.05	0.02	0.03
Glaucoma	0.00	0.00	0.00	0.02	0.01
Heart Failure	0.00	0.00	0.00	0.02	0.00
Hypertension	0.15	0.15	0.11	0.77	0.09
Inflammatory Bowel Disease	0.02	0.02	0.01	0.01	0.04
Irritable Bowel Syndrome	0.01	0.04	0.01	0.01	0.03
Meniere's disease	0.00	0.00	0.00	0.00	0.01
Migraine	0.00	0.22	0.00	0.00	0.00
Multiple Sclerosis	0.00	0.01	0.00	0.00	0.02
Osteoporosis	0.01	0.02	0.01	0.01	0.02
Psychoactive substance misuse	0.01	0.02	0.25	0.00	0.00
Painful condition	0.16	1.00	0.25	0.18	0.04
Parkinson's disease	0.00	0.00	0.00	0.00	0.00
Peripheral vascular disease	0.02	0.03	0.03	0.04	0.05
Pernicious Anaemia	0.01	0.01	0.00	0.00	0.01
Polycystic Ovarian Syndrome	0.01	0.01	0.00	0.01	0.04
Prostate disorders	0.01	0.01	0.01	0.01	0.02
Psoriasis or Eczema	0.01	0.01	0.01	0.01	0.02
Rheumatoid arthritis/ Inflammatory arthropathies/ Connective tissue disease	0.01	0.04	0.00	0.01	0.03
Schizophrenia or bipolar disorder	0.00	0.01	0.09	0.01	0.02
Stroke or TIA	0.01	0.02	0.02	0.04	0.01
Thyroid disease	0.07	0.08	0.03	0.08	0.21
Treated constipation	0.02	0.10	0.05	0.02	0.05
Treated dyspepsia	0.23	0.31	0.23	0.18	0.25
Viral Hepatitis	0.00	0.00	0.00	0.00	0.00

Legend: **Bold red: >=50% prevalence within Cluster; Bold black: 20-59% prevalence within cluster**;

Green background: substantially higher prevalence between clusters (ca double or more);

Yellow background: substantially lower prevalence between clusters (no more than 1/4th of next highest cluster);

Grey: highest between-cluster prevalences, but very low prevalence overall. Mostly ignoring these between cluster differences for naming unless it's a unique appearance of an LTC in one cluster.

Naming Rational for SAIL 37 - 54 years:

Depression is ubiquitous in this age group and sticks out by its absence in one of the clusters rather than its high prevalence in all other clusters.

Pulmonary:
highest in between level of Asthma, Bronchiectasis, and COPD, all pulmonary LCDs. Second-lowest in-between level of Depression for all clusters. Other conditions of similar or substantially lower prevalence than in other clusters.

Pain+ (Incl. Migraine and Rheumatoid Arthritis)**:**
Painful conditions highest in between and within cluster prevalence. Highest-between cluster prevalence of migraine. Constipation highest between cluster prevalence. Other conditions of similar or substantially lower prevalence than in other clusters.

Substance misuse and Mental health:
Highest level of psychoactive substance abuse and alcohol abuse between clusters, highest between-cluster levels of anxiety, and schizophrenia/bipolar, highest level (between and within) of depression. Epilepsy also slightly higher than in other clusters (but less than 3% difference) – common complication of some drug misuse.

Cardiometabolic:
Highest between-cluster level of hypertension, diabetes, chronic kidney disease, and coronary heart disease. Also, atrial fibrillation and stroke highest in this cluster. Lowest level of depression of all clusters. Other conditions of similar or substantially lower prevalence than in other clusters.

Discordant multimorbidity
Highest level of cancer and thyroid disease of all clusters. Highest level of endometriosis, IBD, Meniere's, and lowest level of painful conditions. Only cluster containing multiple sclerosis.

SAIL 55-73 years							
Cluster name	Pulmonary	Pain+ (Incl. Migraine & RA)	Mental health	Substance misuse & Complex multimorbidity	Hypertension+	Cardio-metabolic	Cancer+
#	1	2	3	4	5	6	7
Predicted class membership	0.10	0.17	0.17	0.04	0.34	0.10	0.09
Estimated class population	23796	42802	40803	9001	84063	24734	21694
Long term condition	Conditional item response probabilities						
Alcohol	0.02	0.01	0.05	*0.07*	0.01	0.01	0.00
Anorexia/Bulimia	0.00	0.00	0.00	0.00	0.00	0.00	0.00
Anxiety	0.02	0.05	0.07	0.27	0.01	0.01	0.00
Asthma	*0.60*	0.13	0.15	0.35	0.08	0.07	0.06
Atrial Fibrillation	0.03	0.01	0.02	0.08	0.03	0.18	0.03
Bronchiectasis	0.11	0.00	0.00	0.01	0.00	0.00	0.00
Cancer	0.10	0.10	0.09	0.16	0.07	0.10	1.00
Chronic Fatigue Syndrome	0.00	0.01	0.01	0.01	0.00	0.00	0.00
Chronic Kidney Disease	0.08	0.06	0.07	0.26	0.19	0.28	0.08
Chronic Liver Disease	0.00	0.01	0.01	0.02	0.01	0.01	0.00
Chronic Sinusitis	0.03	0.03	0.06	0.03	0.02	0.01	0.02
COPD	1.00	0.04	0.05	0.36	0.04	0.09	0.04
Coronary Heart Disease	0.07	0.03	0.02	0.21	0.06	0.34	0.04
Dementia	0.00	0.00	0.01	0.03	0.00	0.01	0.00
Depression	0.27	0.40	0.54	0.69	0.21	0.19	0.19
Diabetes	0.15	0.13	0.16	0.40	0.35	0.45	0.13
Diverticular disease	0.06	0.06	0.12	0.16	0.05	0.06	0.07
Endometriosis	0.00	0.00	0.00	0.00	0.00	0.00	0.00
Epilepsy	0.01	0.02	0.03	*0.05*	0.01	0.02	0.01
Glaucoma	0.02	0.01	0.03	0.03	0.04	0.03	0.03
Heart Failure	0.02	0.00	0.00	0.08	0.00	0.14	0.00
Hypertension	0.37	0.32	0.23	0.60	1.00	0.50	0.41
Inflammatory Bowel Disease	0.01	0.02	0.03	0.02	0.01	0.01	0.02
Irritable Bowel Syndrome	0.01	0.04	0.03	0.08	0.01	0.00	0.01
Meniere's disease	0.00	0.01	0.01	0.01	0.01	0.01	0.01
Migraine	0.00	0.13	0.00	0.03	0.00	0.00	0.00
Multiple Sclerosis	0.00	0.01	0.01	0.00	0.00	0.00	0.00
Osteoporosis	0.06	0.06	0.08	0.09	0.02	0.02	0.05
Psychoactive substance misuse	0.01	0.01	0.02	0.07	0.00	0.00	0.00
Painful condition	0.34	1.00	0.14	0.87	0.23	0.33	0.13
Parkinson's disease	0.00	0.00	0.01	0.02	0.00	0.01	0.01
Peripheral vascular disease	0.07	0.05	0.07	0.12	0.06	0.13	0.05
Pernicious Anaemia	0.01	0.01	0.01	0.01	0.01	0.01	0.00
Polycystic Ovarian Syndrome	0.00	0.00	0.00	0.00	0.00	0.00	0.00
Prostate disorders	0.03	0.02	0.04	0.05	0.03	0.07	0.26
Psoriasis or Eczema	0.01	0.01	0.01	0.02	0.01	0.01	0.00
Rheumatoid arthritis/ Inflammatory arthropathies/ Connective tissue disease	0.03	0.06	0.03	0.03	0.01	0.02	0.01
Schizophrenia or bipolar disorder	0.00	0.00	0.03	0.04	0.00	0.00	0.00
Stroke or TIA	0.04	0.03	0.04	0.17	0.06	0.13	0.03
Thyroid disease	0.06	0.09	0.17	0.14	0.09	0.09	0.06
Treated constipation	0.03	0.11	0.07	0.31	0.02	0.04	0.03
Treated dyspepsia	0.23	0.39	0.35	0.32	0.17	0.10	0.17
Viral Hepatitis	0.00	0.00	0.00	0.00	0.00	0.00	0.00

Legend: **Bold red: >=50% prevalence within Cluster**; **Bold black: 20-59% prevalence within cluster**;

Green background: substantially higher prevalence between clusters (ca double or more);

Light blue background: one of two clusters with similar prevalence that's substantially higher than in other clusters

Naming Rational for SAIL 55 - 73 years:

This is one of the most difficult groups to name clusters due to the number of clusters and the added complexity of that. Hypertension is ubiquitous in this age group.

Pulmonary:
Asthma highest in between and within cluster prevalence, Bronchiectasis and COPD highest between cluster prevalence. Other conditions of similar or substantially lower prevalence than in other clusters.

Pain+ (incl. Migraine and Rheumatoid arthritis):
Cluster with 100% painful disorders, although other cluster also have high pain prevalence. Highest between cluster level of migraines, and rheumatoid arthritis which are also pain related. Highest level of dyspepsia, but it's on par with other clusters and correlated with analgesic medication.

Mental health:
Depression has the highest prevalence within the cluster, but it's not the highest level between clusters. No other diseases stick out. This is one of two clusters with a higher prevalence of schizophrenia/bipolar disease and one of two clusters with higher diverticular disease and thyroid disease prevalence. Difficult to name, but probably mental health is best, as it is second rank for both Depression and Schizophrenia.

Substance misuse and Complex multimorbidity:
Highest between cluster level of alcohol and psychoactive substance misuse, highest level of anxiety, one of two clusters with high kidney disease and coronary heart disease, high within cluster level of depression (though not sticking out between levels), one of two clusters with high diverticular disease and peripheral vascular disease. High within cluster prevalence of several LTCs including epilepsy, glaucoma, IBS, constipation. Looks like a lot of the conditions that appear predominantly in this cluster could also be complications from prolonged substance misuse. substance misuse and mental health conditions also cluster in other age groups.

Hypertension+:
Hypertension is the only LTC that sticks out within and between clusters although the difference to the next highest level is not as stark as for other age groups. Hypertension without substantially higher prevalence of related medically significant LTCs. However, this is one of two clusters with higher levels of stroke. Other conditions of similar or substantially lower prevalence than in other clusters.

Cardiometabolic:
Highest between cluster level of atrial fibrillation, one of two clusters with high prevalence of coronary heart disease and chronic kidney disease, highest level for heart failure, one of two clusters with higher levels of peripheral vascular disease and stroke and TIA. High within cluster level of hypertension (though only 3rd highest between clusters). One of two clusters with higher kidney disease levels. Relatively high levels of diabetes, but not standing out between clusters. Overall, driven by combinations of cardiometabolic conditions.

Cancer+:
Highest level of Cancer between and within cluster, high level of prostate disease, likely cancer related (Prostate cancer makes up 31% of cancers in men of the age group and is the most common type of cancer in men in the age group (Cancer Research UK). Hypertension on par with other clusters. Other conditions of similar or substantially lower prevalence than in other clusters.

SAIL 74+ years						
Cluster name	Pulmonary	Pain+	Mental health & Neurological disorders	Hypertension+	Cardiometabolic	Cancer+
	1	2	3	4	5	6
Predicted class membership	0.10	0.19	0.11	0.41	0.09	0.11
Estimated class population, based on modal probability (n)	15432.00	28832.00	16136.00	61886.00	13220.00	17399.00
Long term condition	Conditional item response probabilities					
Alcohol	0.01	0.00	0.02	0.00	0.00	0.00
Anorexia/Bulimia	0.00	0.00	0.00	0.00	0.00	0.00
Anxiety	0.04	0.04	0.16	0.01	0.02	0.01
Asthma	**0.59**	0.13	0.05	0.05	0.10	0.05
Atrial Fibrillation	0.11	0.05	0.10	0.11	**0.48**	0.11
Bronchiectasis	0.10	0.00	0.00	0.00	0.00	0.00
Cancer	0.18	0.17	0.16	0.13	**0.20**	**0.69**
Chronic Fatigue Syndrome	0.00	0.00	0.00	0.00	0.00	0.00
Chronic Kidney Disease	**0.26**	**0.22**	**0.28**	**0.44**	**0.56**	**0.22**
Chronic Liver Disease	0.00	0.01	0.00	0.00	0.00	0.00
Chronic Sinusitis	0.02	0.02	0.01	0.01	0.01	0.01
COPD	**1.00**	0.05	0.07	0.04	0.17	0.06
Coronary Heart Disease	0.13	0.05	0.11	0.13	**0.40**	0.13
Dementia	0.03	0.01	**0.26**	0.03	0.04	0.03
Depression	**0.20**	**0.20**	**0.55**	0.09	**0.22**	0.09
Diabetes	**0.22**	0.15	**0.22**	**0.36**	**0.36**	**0.22**
Diverticular disease	0.13	0.19	0.13	0.06	0.14	0.12
Endometriosis	0.00	0.00	0.00	0.00	0.00	0.00
Epilepsy	0.01	0.01	0.03	0.01	0.01	0.01
Glaucoma	0.06	0.07	0.06	0.09	0.07	0.08
Heart Failure	0.07	0.00	0.03	0.02	**0.47**	0.02
Hypertension	**0.51**	**0.54**	**0.48**	**0.83**	**0.60**	**0.52**
Inflammatory Bowel Disease	0.01	0.02	0.01	0.01	0.01	0.01
Irritable Bowel Syndrome	0.01	0.04	0.02	0.00	0.02	0.01
Meniere's disease	0.01	0.01	0.01	0.01	0.01	0.01
Migraine	0.00	0.01	0.00	0.00	0.00	0.00
Multiple Sclerosis	0.00	0.00	0.00	0.00	0.00	0.00
Osteoporosis	0.12	0.19	0.13	0.05	0.09	0.03
Psychoactive substance misuse	0.01	0.00	0.02	0.00	0.00	0.00
Painful condition	**0.45**	**0.62**	**0.52**	**0.30**	**0.53**	**0.22**
Parkinson's disease	0.01	0.01	0.05	0.01	0.01	0.02
Peripheral vascular disease	0.12	0.11	0.09	0.12	0.18	0.11
Pernicious Anaemia	0.01	0.01	0.02	0.01	0.02	0.01
Polycystic Ovarian Syndrome	0.00	0.00	0.00	0.00	0.00	0.00
Prostate disorders	0.08	0.01	0.04	0.02	0.07	**0.42**
Psoriasis or Eczema	0.01	0.01	0.01	0.01	0.01	0.00
Rheumatoid arthritis/ Inflammatory arthropathies/ Connective tissue disease	0.03	0.05	0.01	0.01	0.02	0.01
Schizophrenia or bipolar disorder	0.00	0.00	0.03	0.00	0.00	0.00
Stroke or TIA	0.10	0.04	**0.24**	0.14	**0.20**	0.12
Thyroid disease	0.09	0.12	0.13	0.12	0.13	0.03
Treated constipation	0.13	0.19	**0.34**	0.04	0.18	0.10
Treated dyspepsia	**0.25**	**0.44**	0.18	0.11	**0.23**	**0.16**
Viral Hepatitis	0.00	0.00	0.00	0.00	0.00	0.00

Legend: **Bold red: >=50% prevalence within Cluster;** **Bold black: 20-59% prevalence within cluster**;

Green background: substantially higher prevalence between clusters (ca double or more);

Yellow background: substantially lower prevalence between clusters (no more than 1/4th of next highest cluster);

Grey: highest between-cluster prevalences, but very low prevalence overall. Mostly ignoring these between cluster differences for naming unless it's a unique appearance of an LTC in one cluster.

Naming Rational for SAIL 74+ years:

As with depression in younger groups, hypertension is ubiquitous. There are also similar and comparatively high levels of Kidney disease, Diabetes, Painful conditions, dyspepsia etc. in almost all clusters.

Pulmonary:
COPD at 100%. Highest between and within prevalence of COPD, highest between-cluster prevalence of Asthma, and Bronchiectasis. Hypertension at background level. Other conditions of similar or substantially lower prevalence than in other clusters.

Pain+:
Highest level of pain conditions although not substantially higher than other clusters due to high background prevalence. Also contains highest between cluster level prevalence of IBS, IBD, Migraine, and Rheumatoid arthritis - all disorders with significant pain. Hypertension is at background level. Chronic liver disease appears only in this cluster, but at a very low level (1%) – treatment related? Other conditions of similar or substantially lower prevalence than in other clusters.

Mental health and Neurological disorders:
Depression and dementia, epilepsy and Parkinson have highest between cluster prevalence. This group combines the highest between cluster level of anxiety and schizophrenia/bipolar = mental health cluster & highest levels of dementia, epilepsy, and Parkinson = neurological cluster Also contains highest prevalence of alcohol misuse and psychoactive substance misuse, though both at low levels (2%). Other conditions of similar or substantially lower prevalence than in other clusters.

Hypertension+:
Hypertension at highest prevalence of all clusters, not much else that stands out. Even common complications that are associated with hypertension in clusters in other age groups are more prevalent in other clusters. This is one of two clusters with the highest diabetes level, but the prevalence is not substantially higher than in other clusters, same with pain. Diverticulitis is lower than in all other clusters. Dyspepsia also slightly lower. "Simple" hypertension? Other conditions of similar or substantially lower prevalence than in other clusters.

Cardiometabolic:
Highest prevalence of atrial fibrillation, coronary heart disease, and heart failure with high levels of vascular disease. Higher level of kidney disease between clusters - complication of heart issues. Hypertension and pain are at background level. Kidney disease highest within level prevalence. Other conditions of similar or substantially lower prevalence than in other clusters.

Cancer+:
Cancer highest prevalence between and within cluster. Hypertension is highly prevalent in all groups and not very high in this group in relative terms. Prostate disorders have highest prevalence between cluster -likely related to cancer – prostate cancer is one of the highest types of cancer in men in this age group (Cancer Research UK).

UKHLS 18-36	Pulmonary	Cardiometabolic	Depression & Asthma
Predicted class membership	0.392	0.364	0.244
Estimated class population, based on modal probability (n)	270	251	168
Long term condition	Conditional item response probabilities		
Asthma	**1.00**	0.10	**1.00**
Arthritis	**0.23**	**0.21**	0.05
Heart failure	0.00	0.00	0.00
Coronary heart disease	0.02	0.06	0.00
Stroke or TIA	0.02	0.04	0.02
Thyroid disease	0.14	0.18	0.01
COPD	0.14	0.06	0.03
Liver disease	0.07	0.14	0.03
Cancer	0.06	0.07	0.02
Diabetes	0.06	**0.23**	0.03
Epilepsy	0.13	0.13	0.00
Hypertension	**0.33**	**0.55**	0.07
Depression	0.10	**0.59**	**1.00**

Legend: **Bold red: >=50% prevalence within Cluster**; **Bold black: 20-59% prevalence within cluster**;

Green background: substantially higher prevalence between clusters (ca double or more);

Yellow background: substantially lower prevalence between clusters (no more than 1/4th of next highest cluster);

Grey: highest between-cluster prevalences, but very low prevalence overall. Mostly ignoring these between cluster differences for naming unless it's a unique appearance of an LTC in one cluster.

Naming Rational for UKHLS 18-36 years:

Pulmonary:

Asthma at 100 %, same as for Cluster 2, but joint by highest between-cluster prevalence of COPD. second highest level of hypertension and one of two clusters high for Arthritis.

Cardiometabolic:

Highest levels of Hypertension, Diabetes, and Coronary heart disease, all count to cardiometabolic. Liver disease also highest between cluster. Depression high within cluster but not between clusters. Arthritis one of two clusters with similar prevalence, same with Epilepsy.

Depression and Asthma:

Asthma and Depression both at 100%, nothing else substantially different than in other clusters (If so, only by being lower prevalence in this cluster)

UKHLS 37-54	Pulmonary	Arthritis+	Diabetes & Hypertension	Cardiovascular	Cancer, Thyroid disease & Depression
Predicted class membership	0.369	0.213	0.096	0.10	0.222
Estimated class population, based on modal probability (n)	805	466	210	218	485
Long term condition	Conditional item response probabilities				
Asthma	**1.00**	0.00	0.07	**0.26**	0.00
Arthritis	**0.33**	**1.00**	0.10	**0.39**	0.00
Heart failure	0.00	0.00	0.01	0.09	0.01
Coronary heart disease	0.01	0.00	0.06	**1.00**	0.02
Stroke or TIA	0.03	0.04	0.01	0.11	0.07
Thyroid disease	0.10	0.11	0.06	0.05	**0.25**
COPD	0.18	0.07	0.00	0.09	0.13
Liver disease	0.05	0.04	0.02	0.08	0.13
Cancer	0.08	0.09	0.00	0.03	**0.20**
Diabetes	0.08	0.13	**1.00**	**0.21**	**0.21**
Epilepsy	0.05	0.04	0.00	0.02	0.10
Hypertension	**0.35**	**0.50**	**1.00**	**0.57**	**0.62**
Depression	**0.36**	**0.38**	0.01	**0.34**	**0.58**

Legend: **Bold red: >=50% prevalence within Cluster**; **Bold black: 20-59% prevalence within cluster**;

Green background: substantially higher prevalence between clusters (ca double or more);

Yellow background: substantially lower prevalence between clusters (no more than 1/4th of next highest cluster);

Grey: highest between-cluster prevalences, but very low prevalence overall. Mostly ignoring these between cluster differences for naming unless it's a unique appearance of an LTC in one cluster.

Naming Rational for UKHLS 37-54 years:

Hypertension is high in all clusters. So is Depression in all but the first cluster.

Pulmonary:
Asthma at 100% and also the highest prevalence of COPD between all clusters. Rest is lower or comparable to other clusters.

Arthritis+:
Arthritis is primary LTC since it is the only LTC that is substantially higher than in other clusters.

Diabetes and Hypertension:
Diabetes and Hypertension at 100% prevalence. Nothing else substantially higher than in other clusters.

Cardiovascular:
Highest level of heart attack (100%, other clusters below 10%). Also highest between-cluster prevalence of Stroke/TIA and heart failure despite lower overall prevalence of these.

Cancer, Thyroid disease, and Depression:
Highest level of Depression in this cluster, even though it is less than twice as high as in the next highest cluster. Depression is generally high in this age group (with the 1st cluster being the exception). Hypertension second highest but comparable to other clusters. Cancer and thyroid disease have the highest between-cluster prevalence which is at least double that of the next highest cluster). Epilepsy is also nearly twice as high as in the next highest cluster.

UKHLS 55-73	Pulmonary	Hypertension+	Cardiovascular	Cancer, Thyroid disease & depression
Predicted class membership	0.177	0.525	0.142	0.156
Estimated class population, based on modal probability (n)	730	2162	584	641
Long term condition	Conditional item response probabilities			
Asthma	**1.00**	0.09	0.21	0.00
Arthritis	**0.58**	**0.54**	**0.48**	**0.76**
Heart failure	0.00	0.01	0.16	0.01
Coronary heart disease	0.08	0.14	**0.82**	0.08
Stroke or TIA	0.03	0.08	0.19	0.07
Thyroid disease	0.11	0.12	0.07	**0.28**
COPD	**0.32**	0.05	0.14	0.11
Liver disease	0.05	0.04	0.04	0.09
Cancer	0.11	0.10	0.10	**0.27**
Diabetes	0.10	**0.31**	**0.33**	**0.25**
Epilepsy	0.02	0.01	0.03	0.04
Hypertension	**0.34**	**1.00**	**0.52**	0.07
Depression	0.16	0.11	0.15	**0.30**

Legend: **Bold red: >=50% prevalence within Cluster;** **Bold black: 20-59% prevalence within cluster**;

Green background: substantially higher prevalence between clusters (ca double or more);

Yellow background: substantially lower prevalence between clusters (no more than 1/4th of next highest cluster);

Grey: highest between-cluster prevalences, but very low prevalence overall. Mostly ignoring these between cluster differences for naming unless it's a unique appearance of an LTC in one cluster.

Naming Rational for UKHLS 55-73 years:

Arthritis is highly prevalent in all clusters in this age group but has not been grouped primarily to one cluster.

Pulmonary:
Asthma at 100%, COPD highest between cluster prevalence. Arthritis high but not different from other clusters and not highest.

Hypertension+:
Hypertension at 100%. None of the other diseases substantially higher in prevalence than in other clusters and in fact mostly within the lower ranges of prevalence compared with other clusters.

Cardiovascular:
Heart failure, coronary heart disease, and stroke are at highest prevalence in this cluster. Nothing else substantially higher.

Cancer, Thyroid disease, and Depression:
Arthritis has the highest within-cluster prevalence, but it is only slightly higher than in other clusters. Highest Between-cluster prevalence of Thyroid disease and Cancer (both more than double than next highest cluster). Highest level of depression, albeit just short of double that of the next highest cluster.

UKHLS 74+	Pulmonary	Arthritis+	Cardiometabolic
Predicted class membership	0.202	0.508	0.29
Estimated class population, based on modal probability (n)	380	959	547
Long term condition	Conditional item response probabilities		
Asthma	**1.00**	0.00	0.00
Arthritis	**0.59**	**1.00**	0.00
Heart failure	0.04	0.05	0.04
Coronary heart disease	0.29	0.31	0.49
Stroke or TIA	0.10	0.11	**0.22**
Thyroid disease	0.08	0.12	0.12
COPD	**0.24**	0.07	0.07
Liver disease	0.03	0.02	0.03
Cancer	0.14	0.15	**0.21**
Diabetes	**0.22**	**0.21**	**0.40**
Epilepsy	0.01	0.01	0.01
Hypertension	**0.55**	**0.70**	**0.78**
Depression	0.06	0.05	0.05

Legend: **Bold red: >=50% prevalence within Cluster**; **Bold black: 20-59% prevalence within cluster**;

Green background: substantially higher prevalence between clusters (ca double or more);

Yellow background: substantially lower prevalence between clusters (no more than 1/4th of next highest cluster);

Grey: highest between-cluster prevalences, but very low prevalence overall. Mostly ignoring these between cluster differences for naming unless it's a unique appearance of an LTC in one cluster.

Naming Rational for UKHLS 55-73 years:

Hypertension is highly prevalent in all clusters in this age group but has not been grouped primarily to one cluster.

Pulmonary:
Asthma at 100%, COPD highest between cluster prevalence. Arthritis within cluster but half that of next highest. Hypertension lowest between cluster.

Arthritis+:
Arthritis at 100%. High within cluster prevalence of Hypertension, but not high between clusters. Other LTCs on par with other clusters.

Cardiometabolic:
Hypertension highest within and between cluster prevalence but similar to other clusters. Highest between cluster prevalence of Diabetes, Stroke. Coronary heart disease also highest between cluster prevalence but not as much of a difference to other clusters than Diabetes and Stroke.

Supplement 3: Clusters of multiple long-term conditions in three UK datasets: a latent class analysis

Note: This supplement has been submitted to the preprint server for Health Sciences (medRxiv) as a pre-print. medRxiv preprint doi: https://doi.org/10.1101/2023.09.05.23294158

Abstract

Introduction: Latent class analysis (LCA) can be used to identify subgroups within populations based on unobserved variables. LCA can be used to explore whether certain long-term conditions (LTC) occur together more frequently than others in patients with multiple-long term conditions. In this manuscript we present findings from applying LCA in three large-scale UK databanks.

Methods: We applied LCA to three different UK databanks: Secure Anonymised Information Linkage databank [SAIL], UK Biobank, and Understanding Society: the UK Household Longitudinal Study [UKHLS] and four different age groups: 18-36, 37-54, 55-73, and 74+ years. The optimal number of classes in each LCA was determined using maximum likelihood. Sample size adjusted Bayesian Information Criterion (aBIC) was used to assess model fit and elbow plots and model entropy were used to assess the best number of latent classes in each model.

Results: Between three to six clusters were identified in the different datasets and age groups. Although different in detail, similar types of clusters were identified between datasets and age groups which combine disorders around similar systems incl. Cardiometabolic clusters, Pulmonary clusters, Mental health clusters, Painful conditions clusters, and cancer clusters.

Introduction

Latent class analysis (LCA) is a method of finite mixture modelling used to identify unobserved subgroups (i.e. 'latent classes') within a population, based on a series of observed indicator variables [31]. LCA assumes that the distribution of the indicator variables is due to the existence of a finite number of underlying latent classes in the population. Typically applied in behavioural and social sciences, LCA has gained traction in medicine as a means of clustering patients into underlying disease phenotypes, based on their clinical and demographic characteristics. LCA has also been used as a method for identifying clusters of multiple long-term conditions that exist across different populations [32-36]. Identifying clusters of multiple LTCs may facilitate deeper understanding and extraction of the underlying cause and effects of multimorbidity profiles on patient and healthcare outcomes.

LCA facilitates the use of statistical inference in the selection of the optimal model solution and does not rely on arbitrary distance-based measures used in other methods for cluster identification (e.g. k-means or hierarchical clustering). As such, LCA is regarded as a more statistically robust and reproducible method of clustering. Additionally, in simulation studies where the underlying distribution of latent classes was known but concealed during analyses, LCA performed better than other clustering algorithms at accurately classifying individuals into the correct cluster [34,37]. In clustering at the individual rather than condition level, LCA may uncover a more accurate reflection of the clinical landscape whereby highly prevalent conditions can appear across several identified multiple long-term condition clusters.

Therefore, in this analysis we apply LCA to identify and characterise clusters of multiple long-term conditions across three UK population datasets.

Methods

Data source

This study is part of an ongoing NIHR-funded Research project “Personalised exercise rehabilitation for people with multiple long-term conditions (PERFORM)”. The UK Biobank has full ethical approval from the NHS National Research Ethics Service (16/NW/0274). This study was conducted as part of UK Biobank Project 14151. The use and analysis of SAIL data was approved by the SAIL information governance review panel (Project 0830). UKHLS data access and use was granted by the UK Data Service (Project ID: 221571).

This work uses data provided by patients and collected by the NHS as part of their care and support, copyright © (2022), NHS England. Re-used with the permission of the NHS England and UK Biobank. All rights reserved.

This research used data assets made available by National Safe Haven as part of the Data and Connectivity National Core Study, led by Health Data Research UK in partnership with the Office for National Statistics and funded by UK Research and Innovation (research which commenced between 1st October 2020 – 31st March 2021 grant ref MC_PC_20029; 1st April 2021 -30th September 2022 grant ref MC_PC_20058)

Approach

We applied LCA to three UK datasets (Secure Anonymised Information Linkage databank [SAIL], UK Biobank, and Understanding Society: the UK Household Longitudinal Study [UKHLS]) to identify clusters of multiple long-term conditions in people with multimorbidity. To account for different morbidity profiles across the lifespan, LCA was applied separately to the following age strata: 18 – 36 years, 37 – 54 years, 55 – 73 years, and 74+ years. LCA analyses were applied participants aged 18+ years at baseline data in both research datasets (UK Biobank & UKHLS), and to adults (aged 18+ years) registered with a participating practice on 1st January 2011 in the unselected community cohort (SAIL). This date was chosen as *de facto* ‘baseline’ in SAIL as electronic data capture was most complete after this period, plus it coincided approximately with baseline recruitment of the other datasets [21].

For LCA, input indicators were single LTCs that were derived from either self-report (UK Biobank & UKHLS) or from Read codes [with associated prescription data for some LTCs] from individually linked healthcare data (SAIL). In our analyses, LTCs were deemed present if self-reported or recorded in linked health data, or otherwise absent, meaning there were no missing input data in our LCA models. Due to heterogeneity of data collection across these datasets, different LTCs were used in the definition of multimorbidity and subsequent construction of LCA models. In UK Biobank and

SAIL, multimorbidity was defined using 43 LTCs derived from a list commonly applied in multimorbidity research [38] (Table 1). In UKHLS, data were collected on only 17 LTCs, some of which were merged to map with LTCs on the longer list (e.g. 'hyperthyroidism' and 'hypothyroidism' were merged to become 'thyroid disease'). Resultantly, a total of 13 LTCs were used to define multimorbidity in UKHLS (Table 2). Only individuals with 2 or more LTCs (i.e. multimorbidity) were included in LCA. The number of people included in LCA per dataset and for each age group is presented in Table S3..

Table S3.1. Number of participants with multimorbidity included in LCA models per age group in each dataset.

Age Strata	Datasets		
	**SAIL (n)**	**UK Biobank (n)**	**UKHLS (n)**
18 - 36 years	53,818	-	689
37 – 54 years	139,943	42,149	2,184
55 – 73 years	246,893	123,008	4,117
74+ years	152,905	-	1,886
Total	593,559	165,157	8,876

UK Biobank included in only 2 of 4 age strata due to age limits of participants. n reflects number of people with multimorbidity (i.e. 2 or more LTCs)

LCA Parameters

LCA was conducted in R using the *poLCA* package [23]. LCA was applied iteratively to identify the optimal number of latent classes in each age group and dataset combination (n = 10). For each combination, we constructed and compared LCA models containing 1 to 10 latent classes. As described above, our indicator variables were single LTCs recorded in each of the datasets (n=43 SAIL & UK Biobank; n=13 UKHLS). *poLCA* uses the expectation-maximization (EM) algorithm with a Newton-Raphson step to identify the maximum likelihood estimates of the model parameters. In our analyses, the EM algorithm was set to complete a maximum of 5000 iterations to achieve model convergence on the maximum likelihood solution. Each LCA model was repeated 25 times to improve the search for a global, rather than local, maximum solution. Model parameters were then validated against models with increasing number of random start values (n = 50 & 100). If the maximum likelihood solution (i.e. the single largest log-likelihood value) could not be replicated from increasing repetitions of model fitting, this indicated poor fit of the data to the specified model and the model was rejected.

Model selection criteria & cluster assignment

Identifying the optimal latent class solution requires careful consideration of multiple criteria. Here, we interpreted the best model through a combined assessment of model fit data with substantive

knowledge and clinical interpretability of clusters. Firstly, we used the Bayesian Information Criterion (BIC) and sample size adjusted BIC (aBIC) to guide model selection. These criteria are derived from the maximum likelihood solution and award model parsimony, with lower values indicating better model fit. Simulation studies have suggested the BIC to be the most reliable model fit indicator, particularly in large samples [39]. Despite this, in LCA models with large sample sizes and high number of indicators (as in our SAIL & UK Biobank analyses), the BIC tends to favour more complex models with a higher number of classes. The BIC was therefore not definitive in our model selection and used as a guide only. We plotted the model fit data using elbow plots, to identify where any subsequent increases in number of latent classes resulted in comparatively lesser improvement in BIC, as has been suggested previously [40]. In addition to this, we assessed the underlying distribution of LTCs within the latent classes for clinical interpretability. Where models may have had similar performance in terms of statistical criteria, the substantive clinical interpretation and face validity of identified clusters were considered prior to model selection. Finally, we considered model entropy, with higher values (close to 1) representing models with more accurate assignment of individuals into the appropriate classes.

After identifying the optimal LCA solution, individuals were assigned to the latent class for which they had the highest posterior probability. Conditional item response probabilities were used to investigate within cluster prevalence of each individual LTC, then the clusters were subsequently labelled based on within and between cluster prevalence of these LTCs, as detailed below.

Applied convention for labelling MLTC clusters:

Considered between cluster differences first, include LTCs that had substantially higher prevalence in one cluster compared to all others in name (rule of thumb approximately twice than next highest cluster)

Labels include LTCs with 100% within-cluster prevalence and/or the highest between-cluster prevalence

Labelled according to affected systems where LTCs with highest within and between prevalence affect similar physiology and body systems (e.g. Pulmonary, cardiovascular, etc.)

Used '+' when cluster named after single condition with highest within and/or between cluster prevalence

Where a single LTC dominates the cluster , a + was added to the name to indicate that a collection of other LTCs are also in this cluster albeit without other obvious between-cluster differences that warrant naming

Clusters with several LTCs with high within and between cluster prevalence which affect multiple systems and without one single dominant LTC in the cluster, were labelled Discordant

Note:

The clusters have been named for convenience in discussion and write-up. Clusters are complex and no naming convention will comprehensively describe a cluster. Readers are advised to investigate the prevalence of LTCs in detail when interpreting the data and results.

Results

Identified MLTC clusters

Below we present elbow plots containing model fit criteria and statistics and rationale for selection of the optimal LCA model in each age group and dataset combination.

SAIL LCA Models

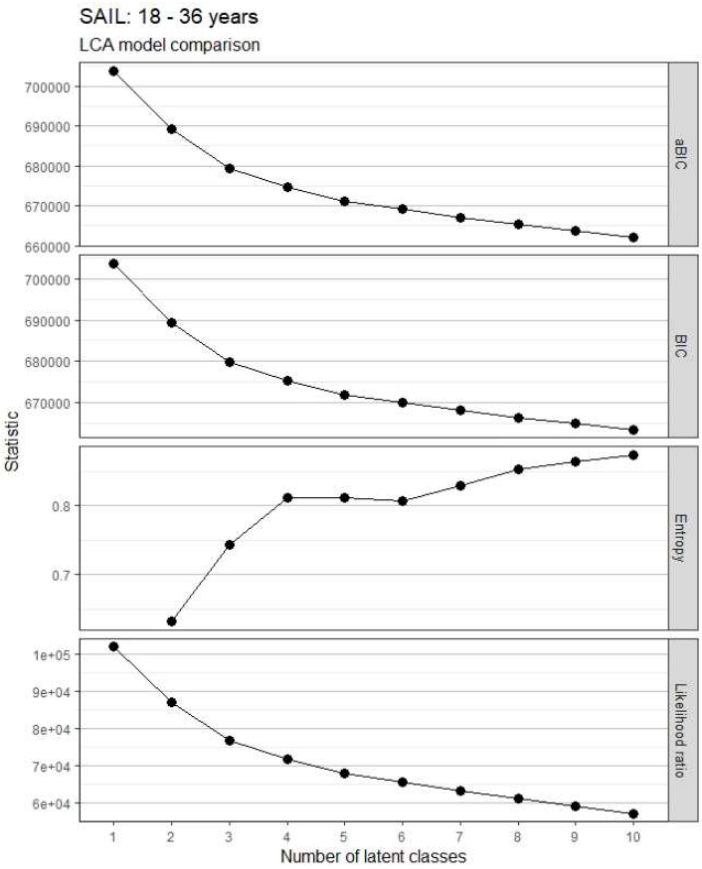

Figure S3.1. Model fit statistics for LCA models in adults 18-36 years in SAIL

Model selected: 5-class model.

Reason: Stepwise improvement in aBIC and BIC was reduced beyond the 5-class solution. Only local maxima were identified in 6-class model and beyond, meaning poor data fit and models being rejected. 5-class model had good clinical interpretability and better fit statistics than 4-class model, with almost identical classification.

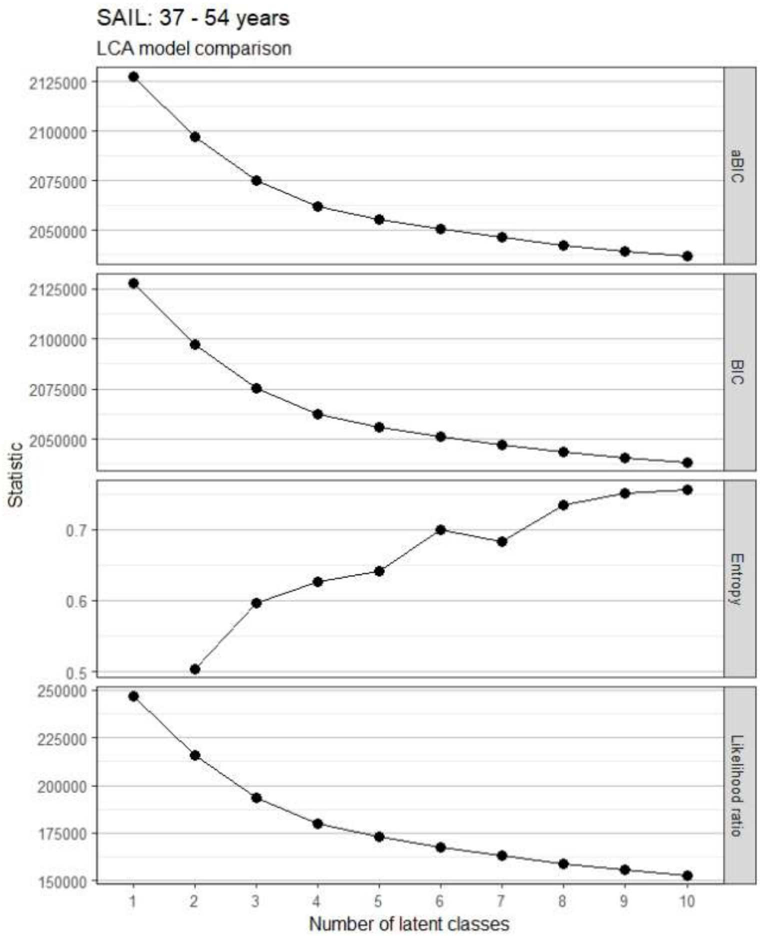

Figure S3.2. Model fit statistics for LCA models in adults 37-54 years in SAIL

Model selected: 5-class model.

Rationale: Stepwise improvement in aBIC and BIC reduces beyond the 5-class model. Failure to replicate maximum likelihood function in 7-class model onwards. Marginal improvement in model classification between 5-class and 6-class model, but 6-class did not improve clinical interpretability.

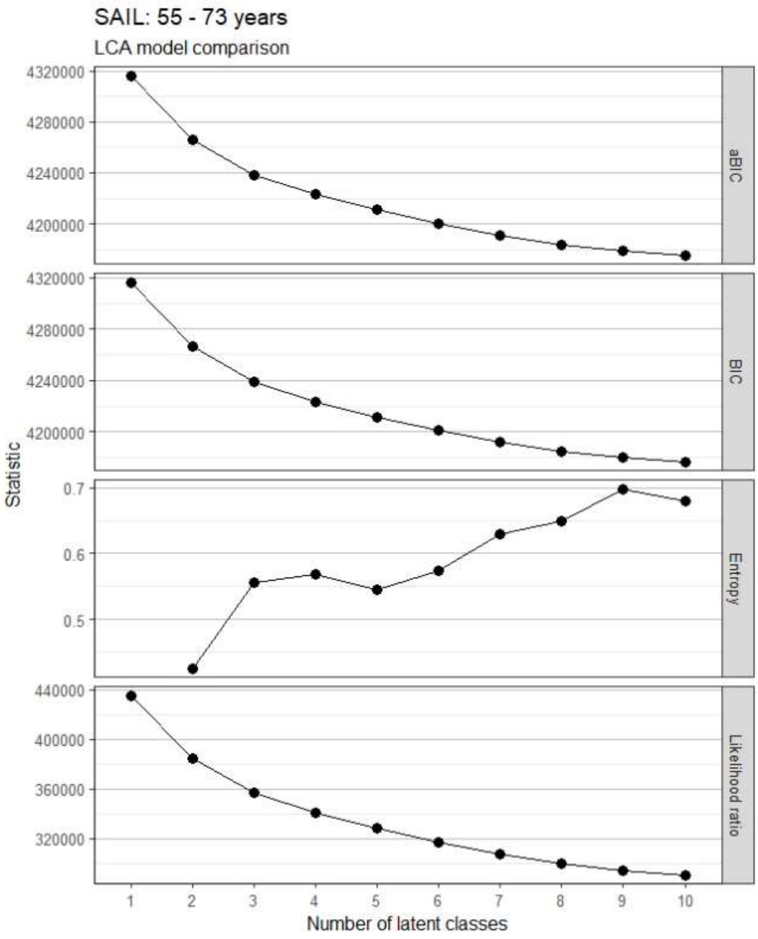

Figure S3.3. Model fit statistics for LCA models in adults 55 - 73 years in SAIL

Model selected: 7-class model.

Rationale: Improvement in aBIC and BIC smaller after 6-class model. Classification improves in 7-class model. Clinical interpretation of 7-class model better than that of additional models with small differences in fit criteria and classification.

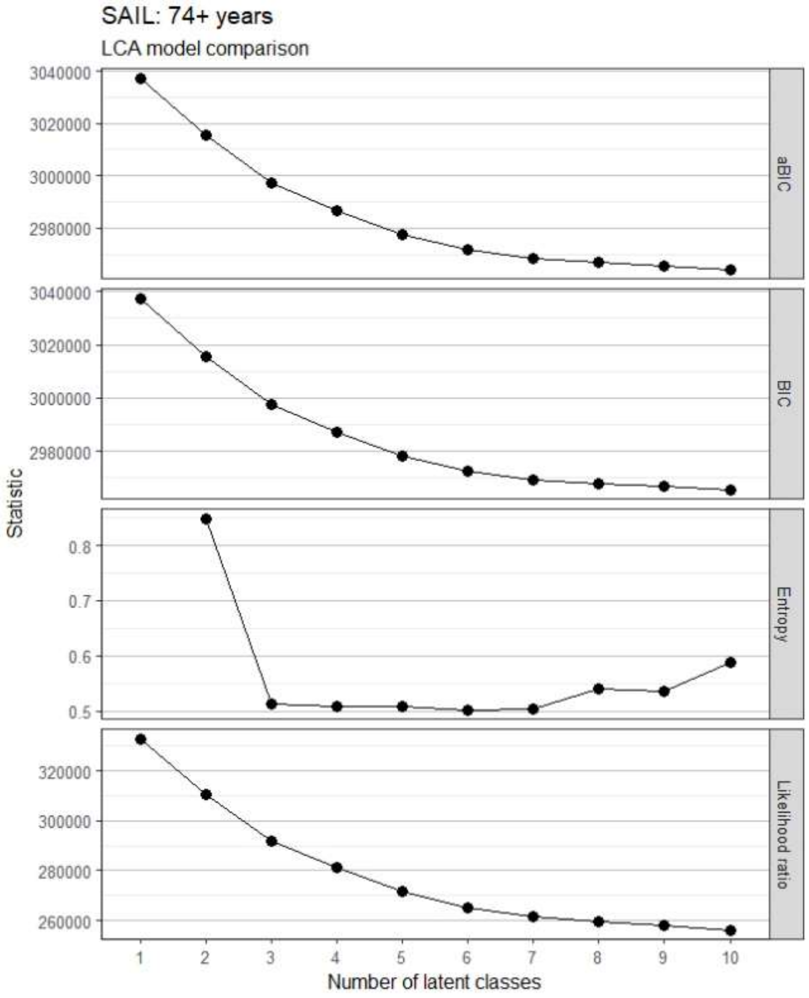

Figure S3.4. Model fit statistics for LCA models in adults 55 - 73 years in SAIL

Model selected: 6-class model.

Rationale: Stepwise improvement in aBIC and BIC reduces around 5 to 6-class model. All models beyond 2-class model have similar classification. Two class model not appropriate as showed very similar clinical profiles split only by the presence/absence of COPD. Similar model fit between 6-class and 7-class, but clinical interpretation favourable in 6-class model.

UK Biobank LCA Models

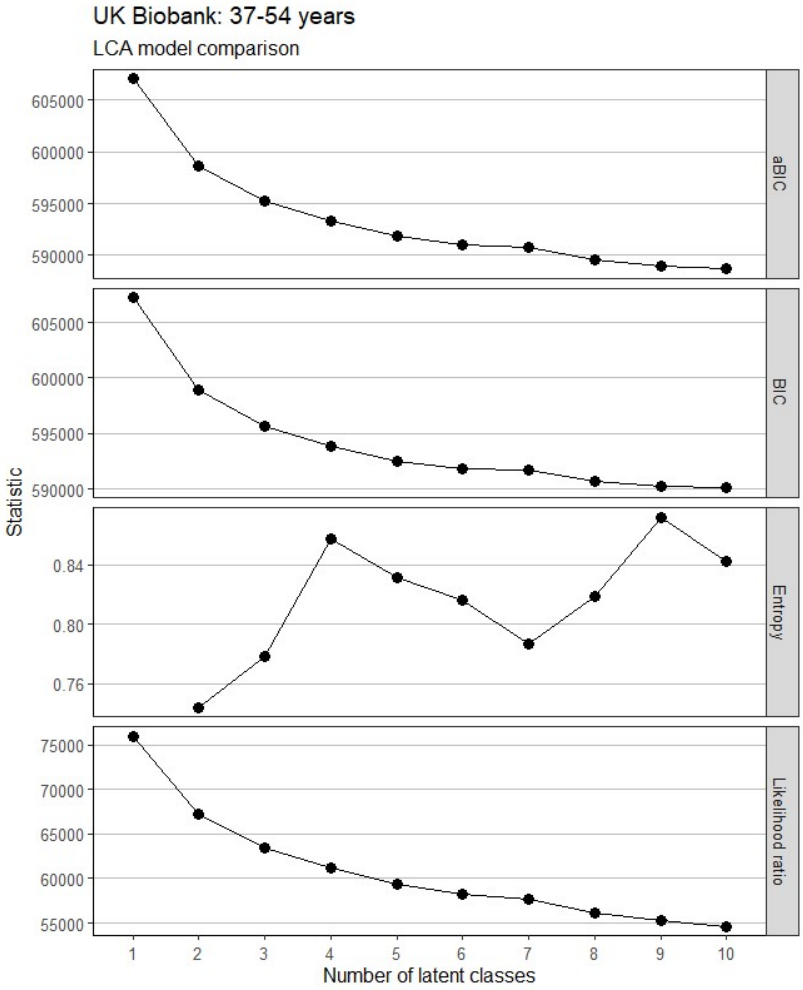

Figure S3.5. Model fit statistics for LCA models in adults 37 – 54 years in UK Biobank

Model selected: 5-class model.

Rationale: Stepwise improvement in aBIC and BIC reduced beyond the 5-class model. Failed to replicate maximum log-likelihood function in 6-class model onwards, so models rejected due to instability and poor data fit.

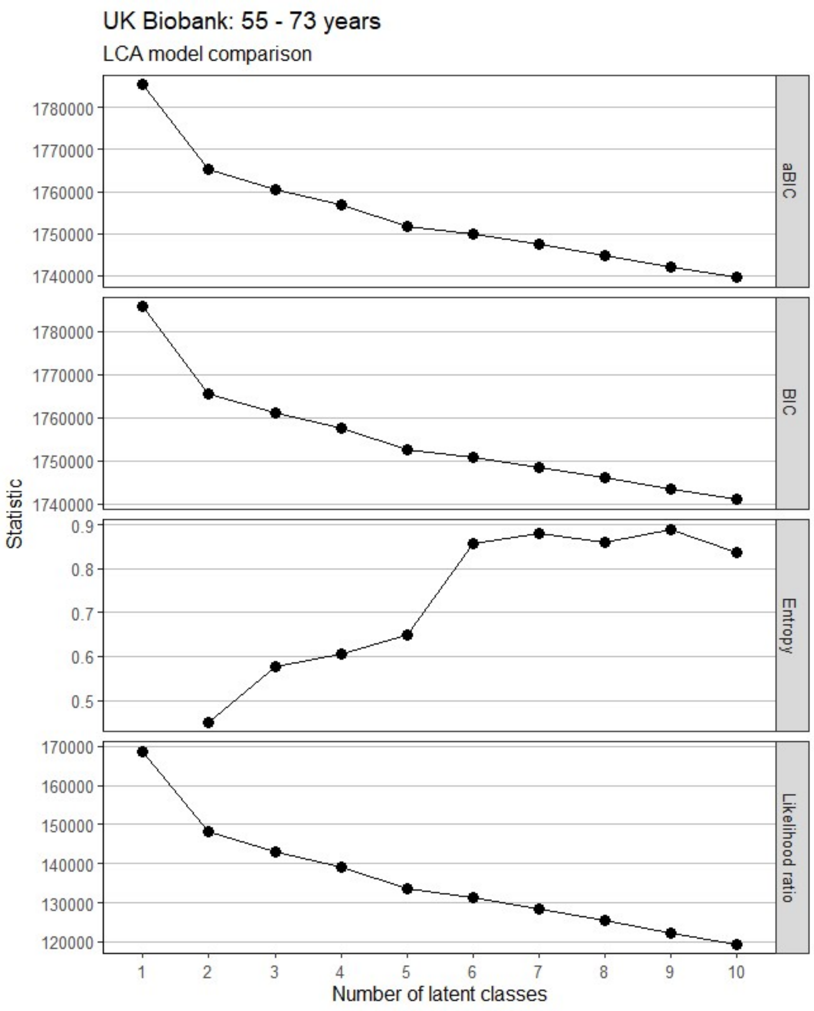

Figure S3.6. Model fit statistics for LCA models in adults 55 - 73 years in UK Biobank

Model selected: 4-class model.

Rationale: Stepwise improvement in aBIC and BIC reduced beyond the 5-class model. However, failed to replicate maximum log-likelihood function in 5-class model onwards, so these models were rejected due to instability and poor data fit.

UKHLS LCA Models

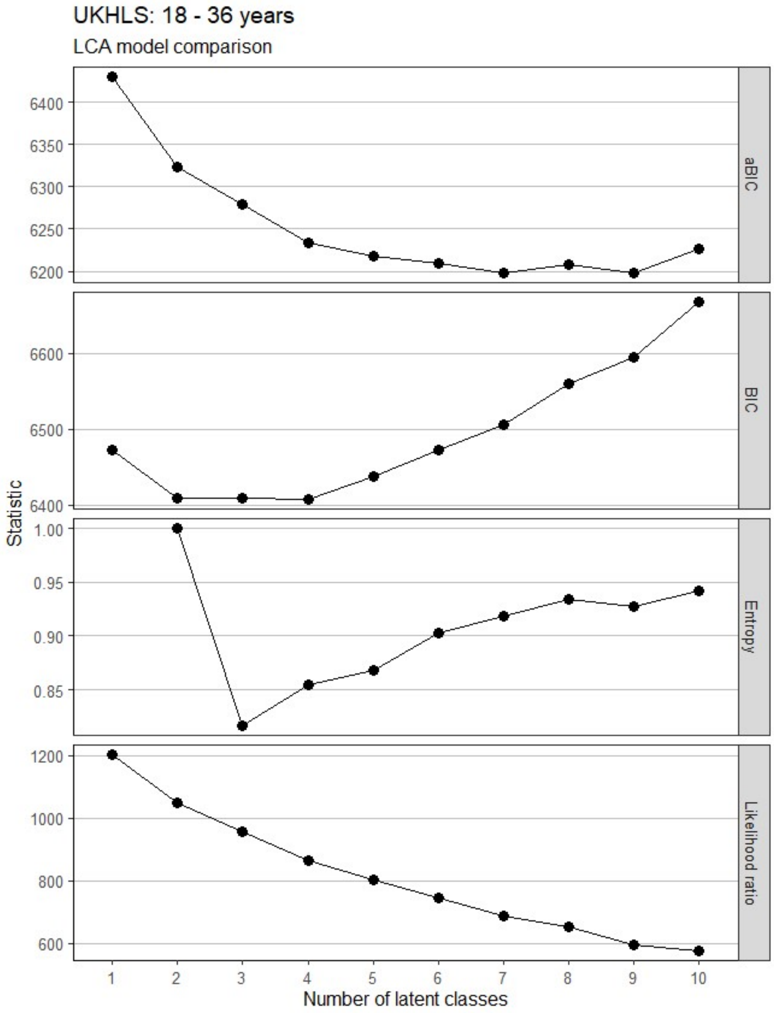

Figure S3.7. Model fit statistics for LCA models in adults 18 – 36 years in UKHLS

Model selected: 3-class model.

Rationale: BIC almost identical in 2, 3 and 4-class models, reduction in aBIC improvement beyond 4-class model. Failed to replicate maximum log-likelihood in 4-class models and beyond so these were rejected. Clinical interpretation of 3-class model preferable vs 2-class model. Classification of this model was still good despite being the lowest among comparable models.

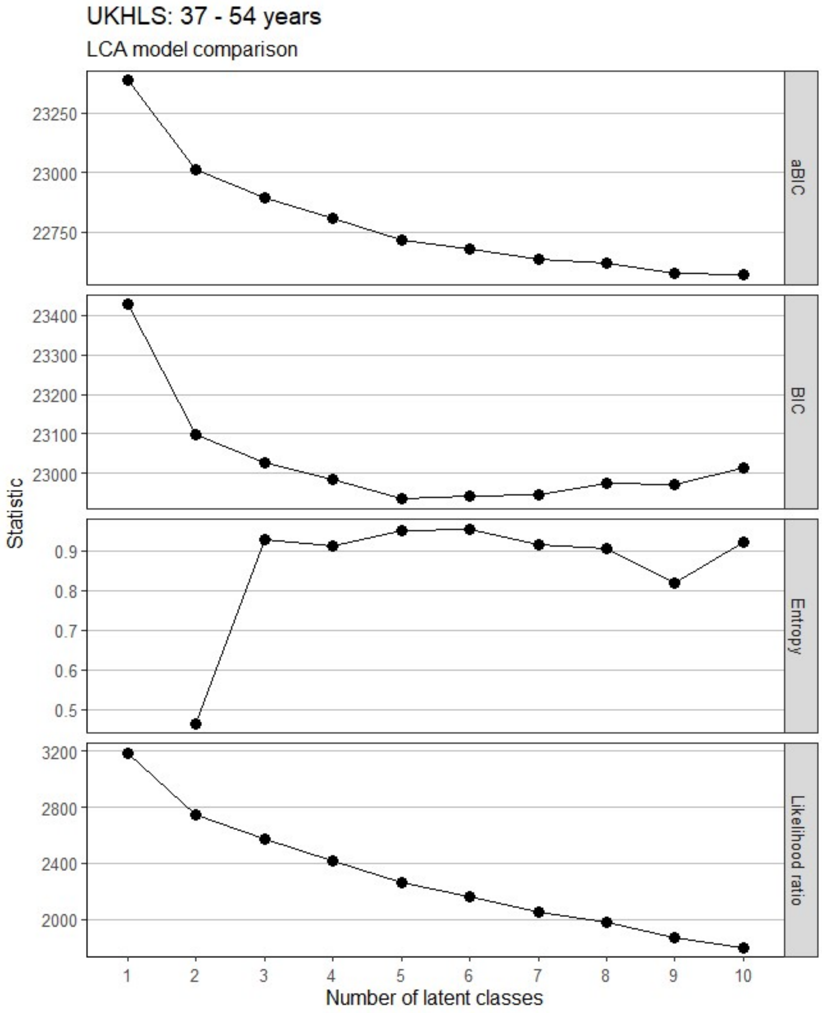

Figure S3.8. Model fit statistics for LCA models in adults 37 - 54 years in UKHLS

Model selected: 5-class model.

Rationale: Lowest BIC and deflection of aBIC at 5-class model. Larger models were unstable and therefore rejected. Very clear clinical interpretation.

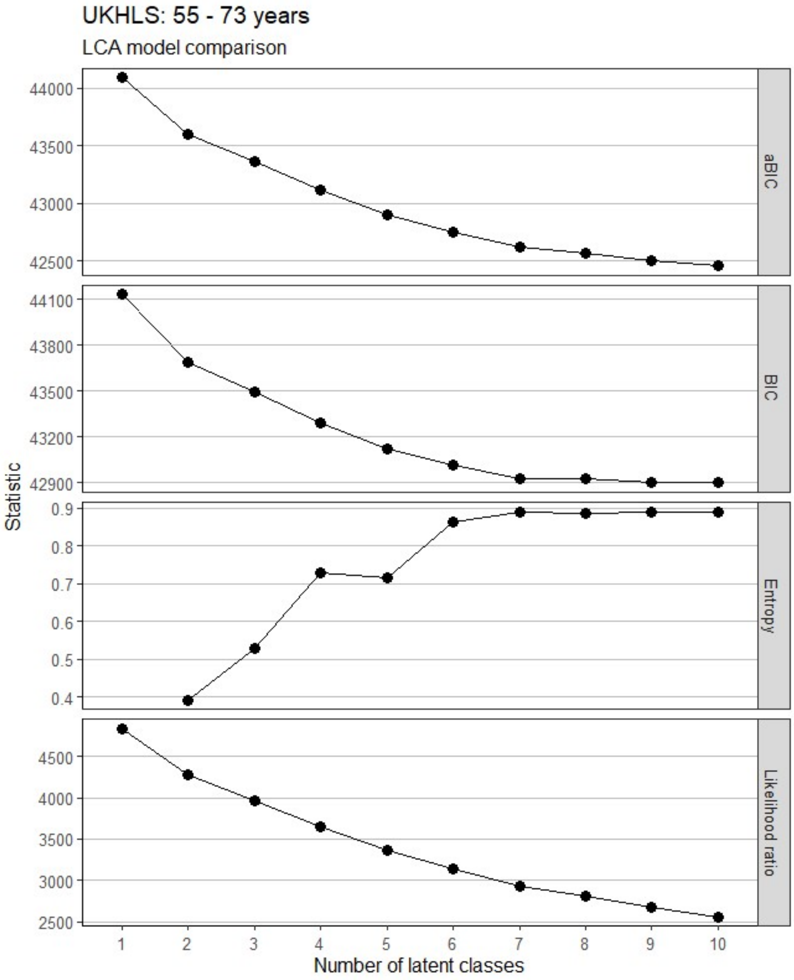

Figure S3.9. Model fit statistics for LCA models in adults 55 – 73 years in UKHLS

Model selected: 4-class model.

Rationale: BIC and aBIC showed consistent improvement until 7-class model, but maximum log-likelihood function could not be replicated in any models beyond the 4-class model. Indeed, the EM algorithm failed to converge after 5000 iterations in the 7 to 10-class models, highlighting instability of these models. 4-class model had improved classification and clinical interpretation compared to smaller models.

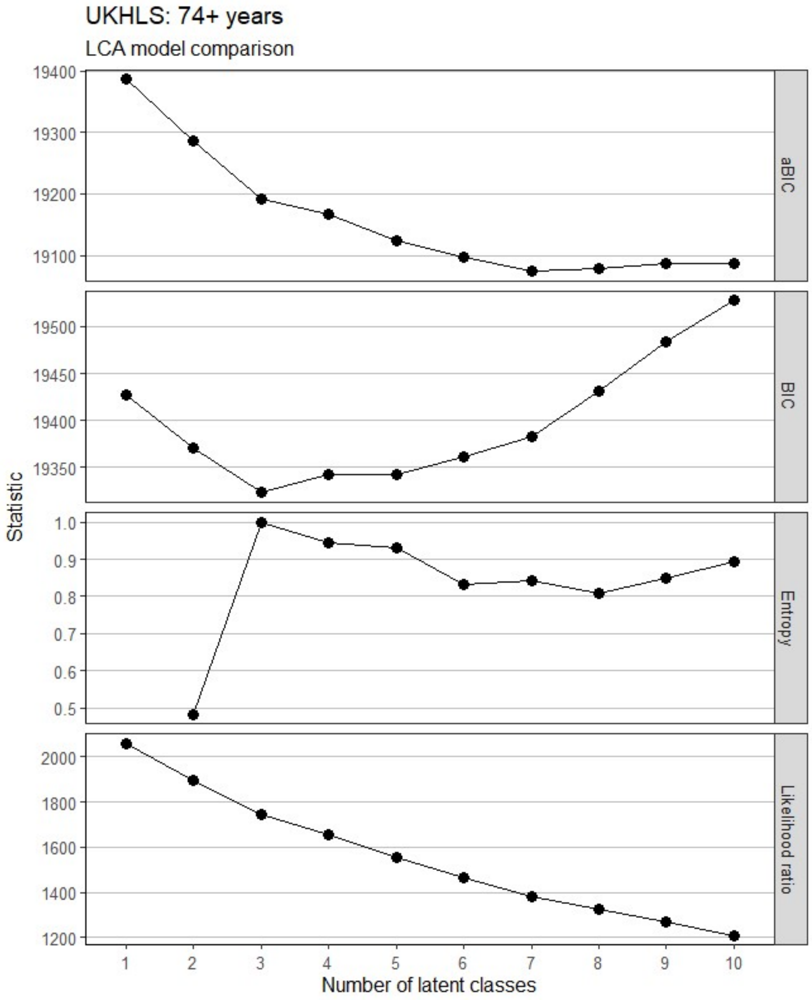

Figure S3.10. Model fit statistics for LCA models in adults 74+ years in UKHLS

Model selected: 3-class model.

Rationale: Lowest BIC and reduced improvement of aBIC beyond the 3-class model. Model classification in 3-class model is ideal (entropy = 1). Model failed to converge in 4-class and 6-class model, and maximum likelihood function could not be replicated in 7-class model onwards.

MLTC clusters:

SAIL MLTC Clusters

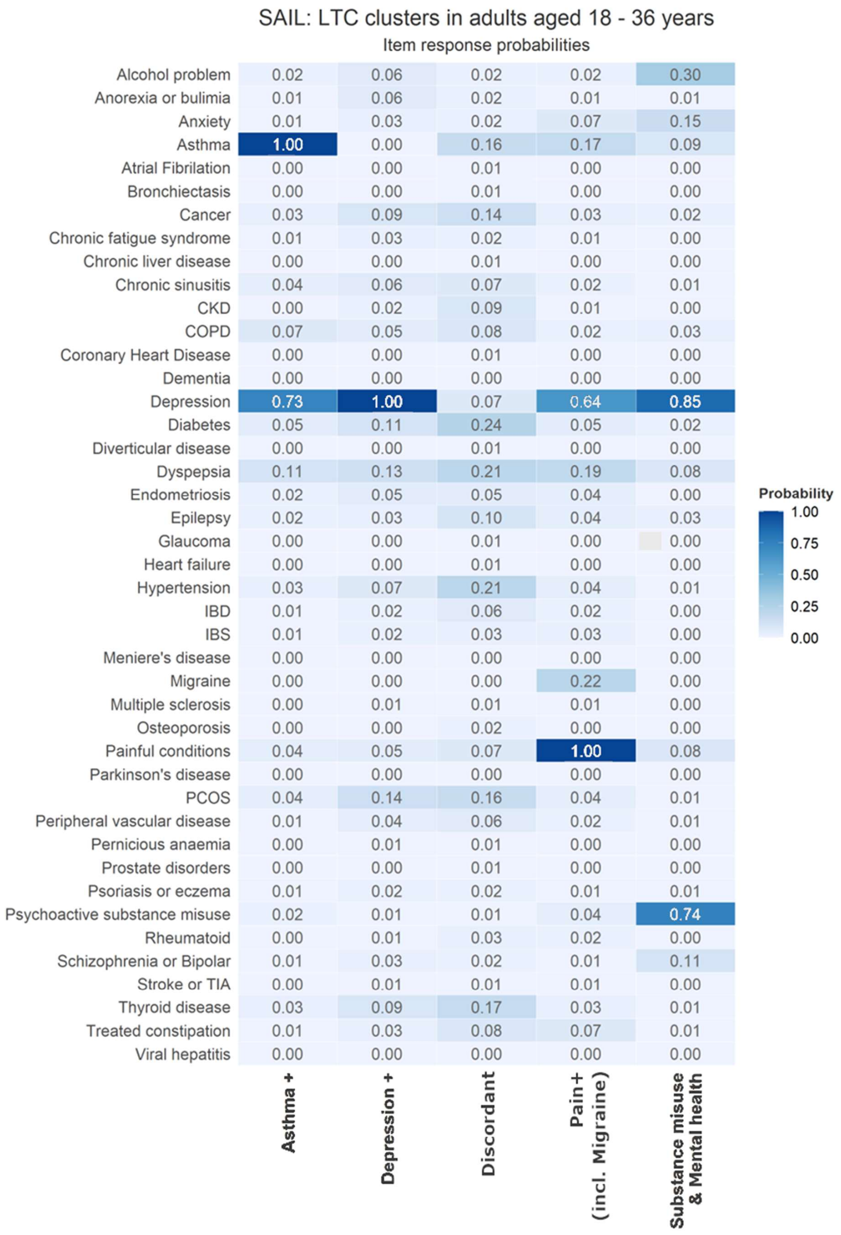

Figure S3.11.

Within cluster conditional item response probabilities for adults 18 - 36 years in SAIL.

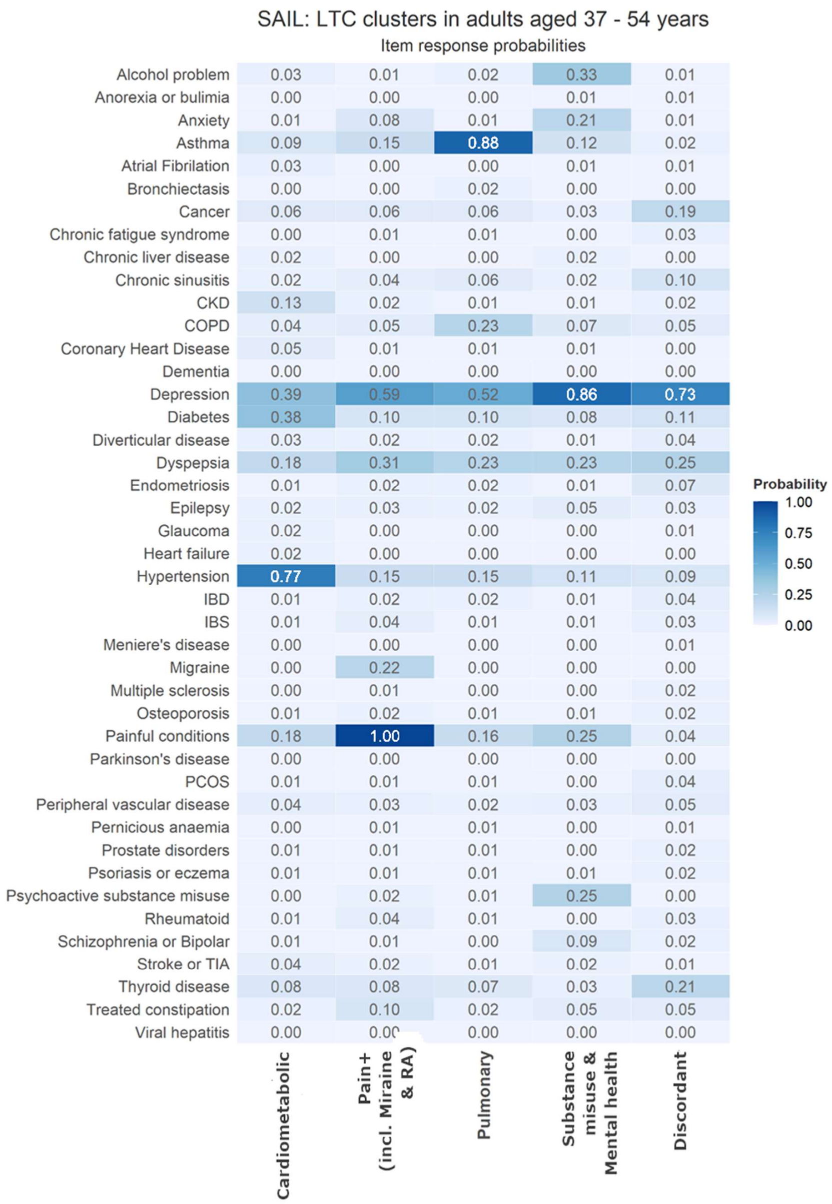

Figure S3.12.

Within cluster conditional item response probabilities for adults 37 - 54 years in SAIL

SAIL: LTC clusters in adults aged 55 - 73 years

Item response probabilities

	Cancer +	Cardiometabolic	Substance misuse & discordant	Hypertension +	Mental health	Pain+ (incl. Migraine & RA)	Pulmonary
Alcohol problem	0.00	0.01	0.07	0.01	0.05	0.01	0.02
Anorexia or bulimia	0.00	0.00	0.00	0.00	0.00	0.00	0.00
Anxiety	0.00	0.01	0.27	0.01	0.07	0.05	0.02
Asthma	0.06	0.07	0.35	0.08	0.15	0.13	0.60
Atrial Fibrilation	0.03	0.18	0.08	0.03	0.02	0.01	0.03
Bronchiectasis	0.00	0.00	0.01	0.00	0.00	0.00	0.11
Cancer	1.00	0.10	0.16	0.07	0.09	0.10	0.10
Chronic fatigue syndrome	0.00	0.00	0.01	0.00	0.01	0.01	0.00
Chronic liver disease	0.00	0.01	0.02	0.01	0.01	0.01	0.00
Chronic sinusitis	0.02	0.01	0.03	0.02	0.06	0.03	0.03
CKD	0.08	0.28	0.26	0.19	0.07	0.06	0.08
COPD	0.04	0.09	0.36	0.04	0.05	0.04	1.00
Coronary Heart Disease	0.04	0.34	0.21	0.06	0.02	0.03	0.07
Dementia	0.00	0.01	0.03	0.00	0.01	0.00	0.00
Depression	0.19	0.19	0.69	0.21	0.54	0.40	0.27
Diabetes	0.13	0.45	0.40	0.35	0.16	0.13	0.15
Diverticular disease	0.07	0.06	0.16	0.05	0.12	0.07	0.06
Dyspepsia	0.17	0.10	0.32	0.17	0.35	0.39	0.23
Endometriosis	0.00	0.00	0.00	0.00	0.00	0.00	0.00
Epilepsy	0.01	0.02	0.05	0.01	0.03	0.02	0.01
Glaucoma	0.03	0.03	0.03	0.04	0.03	0.01	0.02
Heart failure	0.00	0.14	0.08	0.00	0.00	0.00	0.02
Hypertension	0.41	0.50	0.60	1.00	0.23	0.32	0.37
IBD	0.02	0.01	0.02	0.01	0.03	0.02	0.01
IBS	0.01	0.00	0.08	0.01	0.03	0.04	0.01
Meniere's disease	0.01	0.01	0.01	0.01	0.01	0.01	0.00
Migraine	0.00	0.00	0.03	0.00	0.00	0.13	0.00
Multiple sclerosis	0.00	0.00	0.00	0.00	0.01	0.01	0.00
Osteoporosis	0.05	0.02	0.09	0.02	0.08	0.06	0.06
Painful conditions	0.13	0.33	0.87	0.23	0.14	1.00	0.34
Parkinson's disease	0.01	0.01	0.02	0.00	0.01	0.00	0.00
PCOS	0.00	0.00	0.00	0.00	0.00	0.00	0.00
Peripheral vascular disease	0.05	0.13	0.12	0.06	0.07	0.05	0.07
Pernicious anaemia	0.00	0.01	0.01	0.01	0.01	0.01	0.01
Prostate disorders	0.26	0.07	0.05	0.03	0.04	0.02	0.03
Psoriasis or eczema	0.00	0.01	0.02	0.01	0.01	0.01	0.01
Psychoactive substance misuse	0.00	0.00	0.07	0.00	0.02	0.01	0.01
Rheumatoid	0.01	0.02	0.03	0.01	0.03	0.06	0.03
Schizophrenia or Bipolar	0.00	0.00	0.04	0.00	0.03	0.00	0.00
Stroke or TIA	0.03	0.13	0.17	0.06	0.04	0.03	0.04
Thyroid disease	0.06	0.09	0.14	0.09	0.17	0.09	0.06
Treated constipation	0.03	0.04	0.32	0.02	0.07	0.11	0.03
Viral hepatitis	0.00	0.00	0.00	0.00	0.00	0.00	0.00

Figure S3.13.

Within cluster conditional item response probabilities for adults 55 - 73 years in SAIL.

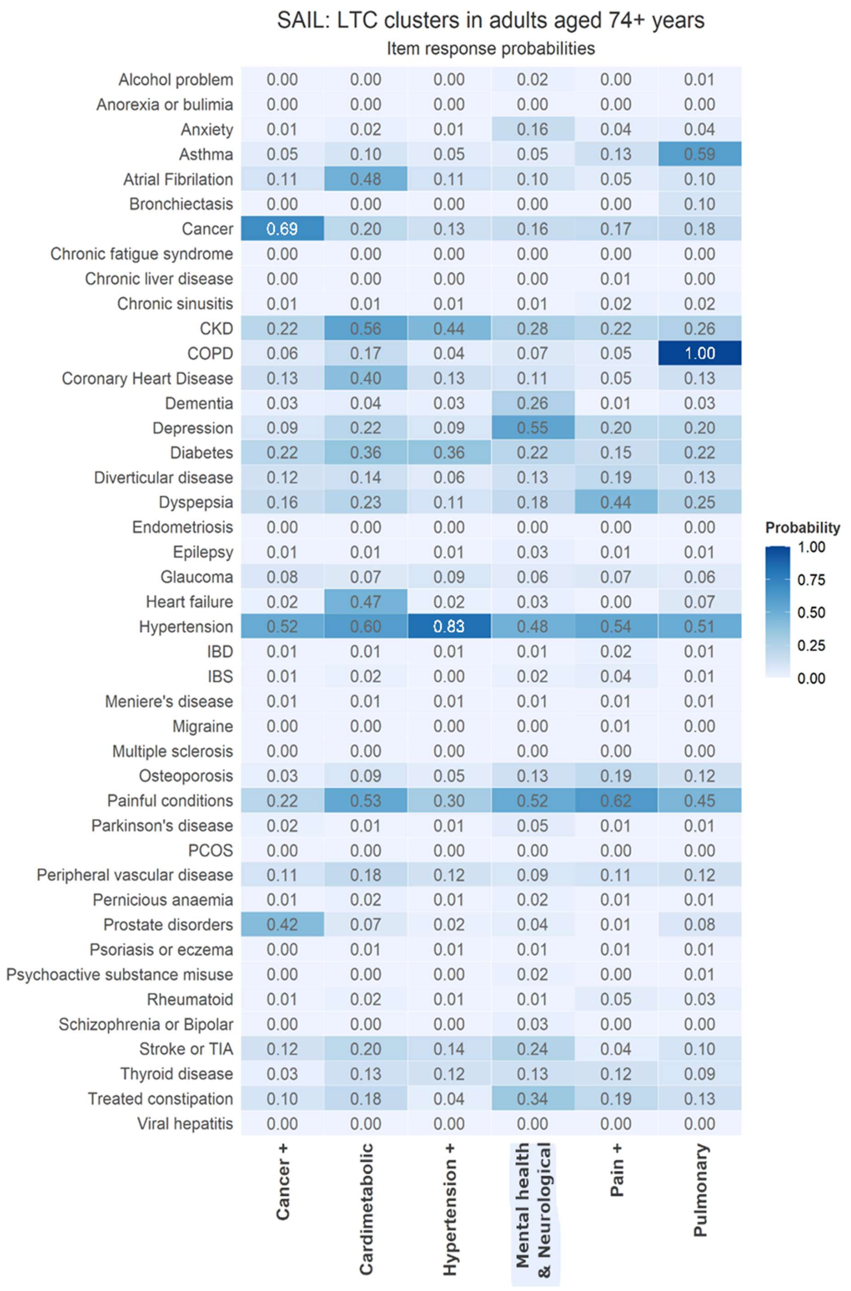

Figure S3.14.

Within cluster conditional item response probabilities for adults 74+ years in SAIL.

UK Biobank MLTC clusters

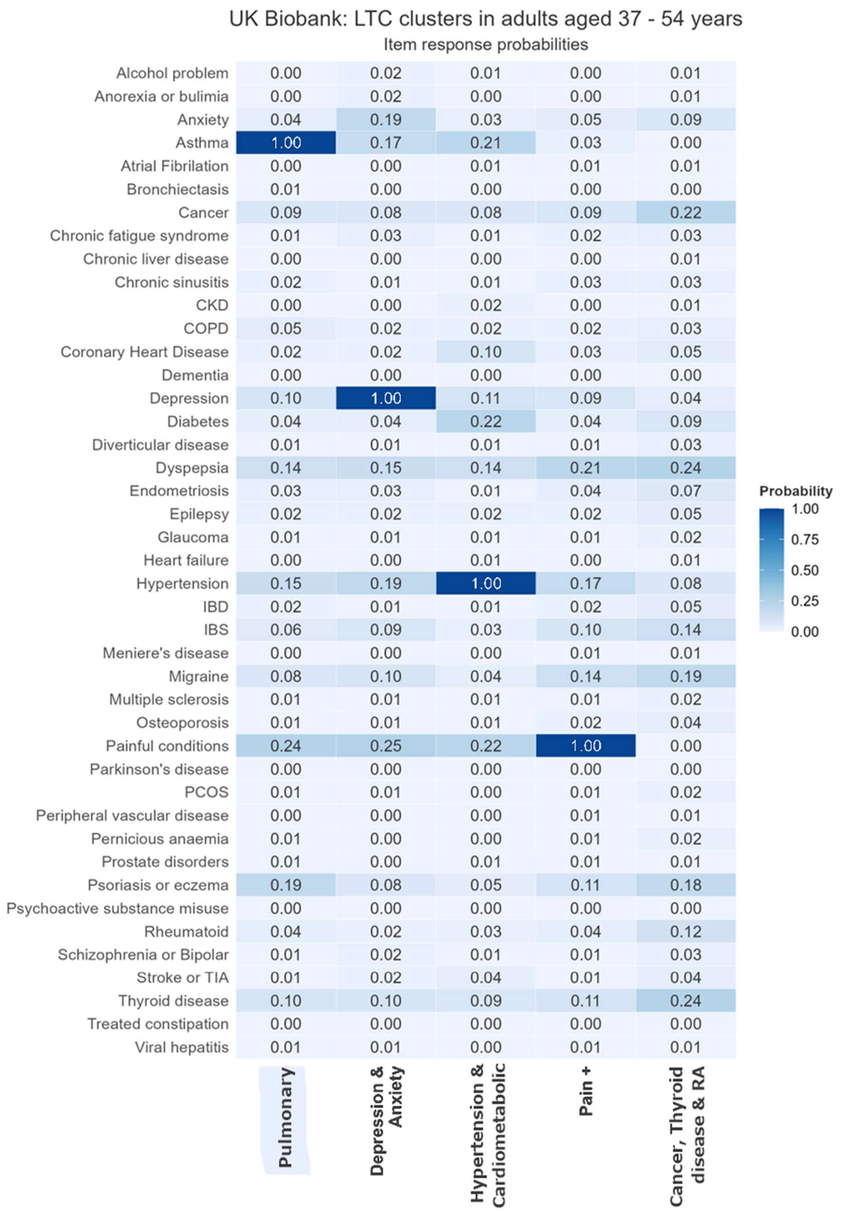

	Pulmonary	Depression & Anxiety	Hypertension & Cardiometabolic	Pain +	Cancer, Thyroid disease & RA
Alcohol problem	0.00	0.02	0.01	0.00	0.01
Anorexia or bulimia	0.00	0.02	0.00	0.00	0.01
Anxiety	0.04	0.19	0.03	0.05	0.09
Asthma	1.00	0.17	0.21	0.03	0.00
Atrial Fibrilation	0.00	0.00	0.01	0.01	0.01
Bronchiectasis	0.01	0.00	0.00	0.00	0.00
Cancer	0.09	0.08	0.08	0.09	0.22
Chronic fatigue syndrome	0.01	0.03	0.01	0.02	0.03
Chronic liver disease	0.00	0.00	0.00	0.00	0.01
Chronic sinusitis	0.02	0.01	0.01	0.03	0.03
CKD	0.00	0.00	0.02	0.00	0.01
COPD	0.05	0.02	0.02	0.02	0.03
Coronary Heart Disease	0.02	0.02	0.10	0.03	0.05
Dementia	0.00	0.00	0.00	0.00	0.00
Depression	0.10	1.00	0.11	0.09	0.04
Diabetes	0.04	0.04	0.22	0.04	0.09
Diverticular disease	0.01	0.01	0.01	0.01	0.03
Dyspepsia	0.14	0.15	0.14	0.21	0.24
Endometriosis	0.03	0.03	0.01	0.04	0.07
Epilepsy	0.02	0.02	0.02	0.02	0.05
Glaucoma	0.01	0.01	0.01	0.01	0.02
Heart failure	0.00	0.00	0.01	0.00	0.01
Hypertension	0.15	0.19	1.00	0.17	0.08
IBD	0.02	0.01	0.01	0.02	0.05
IBS	0.06	0.09	0.03	0.10	0.14
Meniere's disease	0.00	0.00	0.00	0.01	0.01
Migraine	0.08	0.10	0.04	0.14	0.19
Multiple sclerosis	0.01	0.01	0.01	0.01	0.02
Osteoporosis	0.01	0.01	0.01	0.02	0.04
Painful conditions	0.24	0.25	0.22	1.00	0.00
Parkinson's disease	0.00	0.00	0.00	0.00	0.00
PCOS	0.01	0.01	0.00	0.01	0.02
Peripheral vascular disease	0.00	0.00	0.00	0.01	0.01
Pernicious anaemia	0.01	0.00	0.00	0.01	0.02
Prostate disorders	0.01	0.00	0.01	0.01	0.01
Psoriasis or eczema	0.19	0.08	0.05	0.11	0.18
Psychoactive substance misuse	0.00	0.00	0.00	0.00	0.00
Rheumatoid	0.04	0.02	0.03	0.04	0.12
Schizophrenia or Bipolar	0.01	0.02	0.01	0.01	0.03
Stroke or TIA	0.01	0.02	0.04	0.01	0.04
Thyroid disease	0.10	0.10	0.09	0.11	0.24
Treated constipation	0.00	0.00	0.00	0.00	0.00
Viral hepatitis	0.01	0.01	0.00	0.01	0.01

Figure S3.15.

Within cluster conditional item response probabilities for adults 37 - 54 years in UK Biobank.

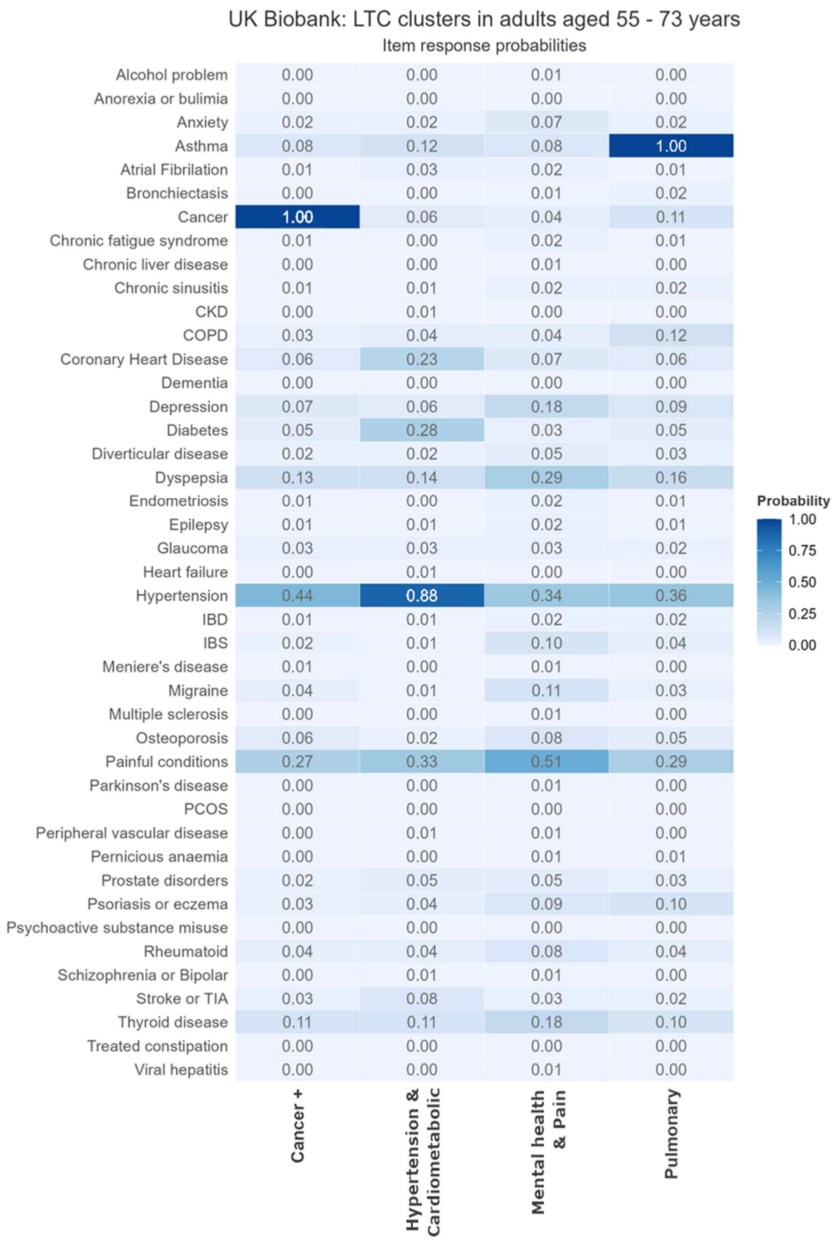

Figure S3.16.

Within cluster conditional item response probabilities for adults 55-73 years in UK Biobank.

UKHLS MLTC clusters

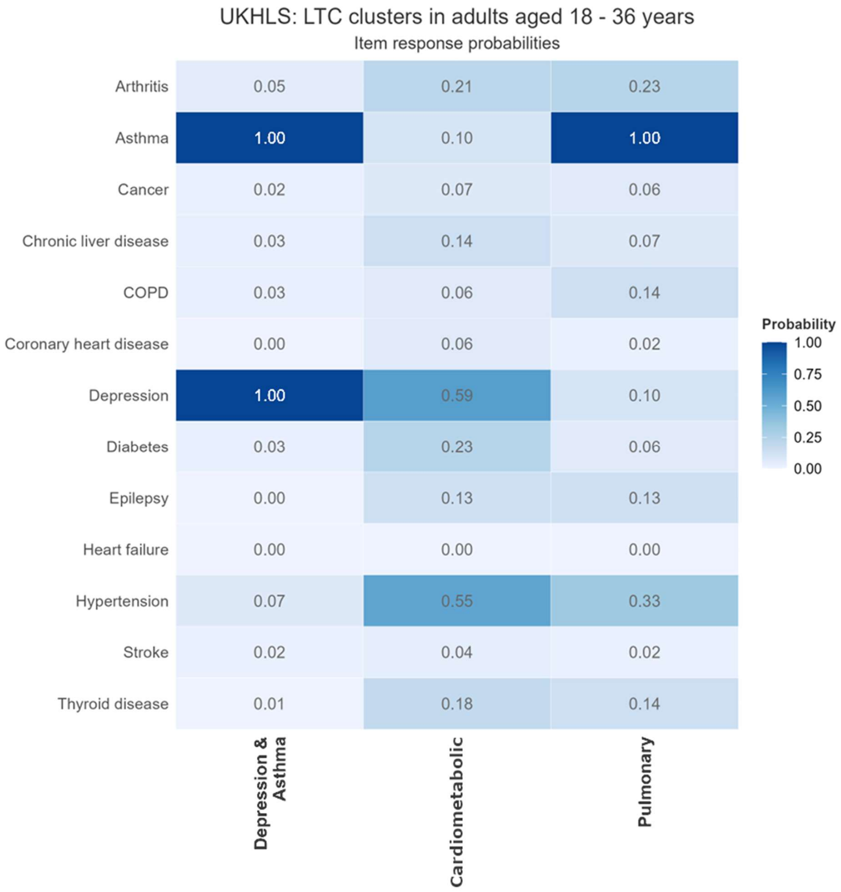

Figure S3.17.

Within cluster conditional item response probabilities for adults 18 - 36 years in UKHLS.

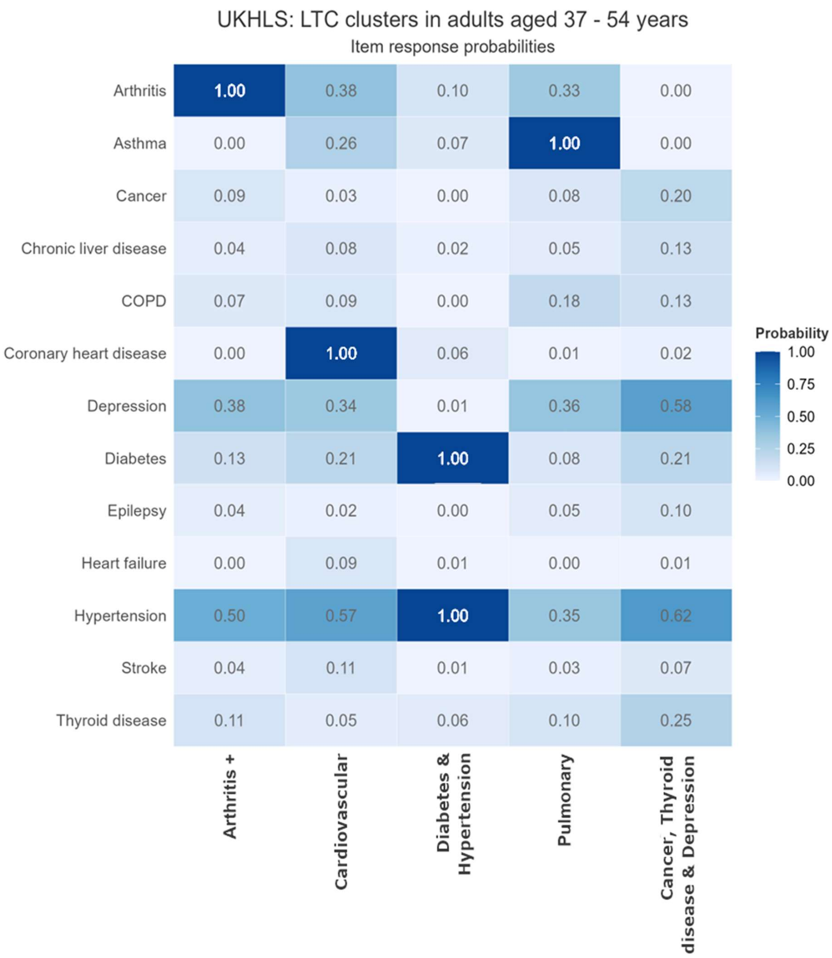

Figure S3.18.

Within cluster conditional item response probabilities for adults 37 – 54 years in UKHLS.

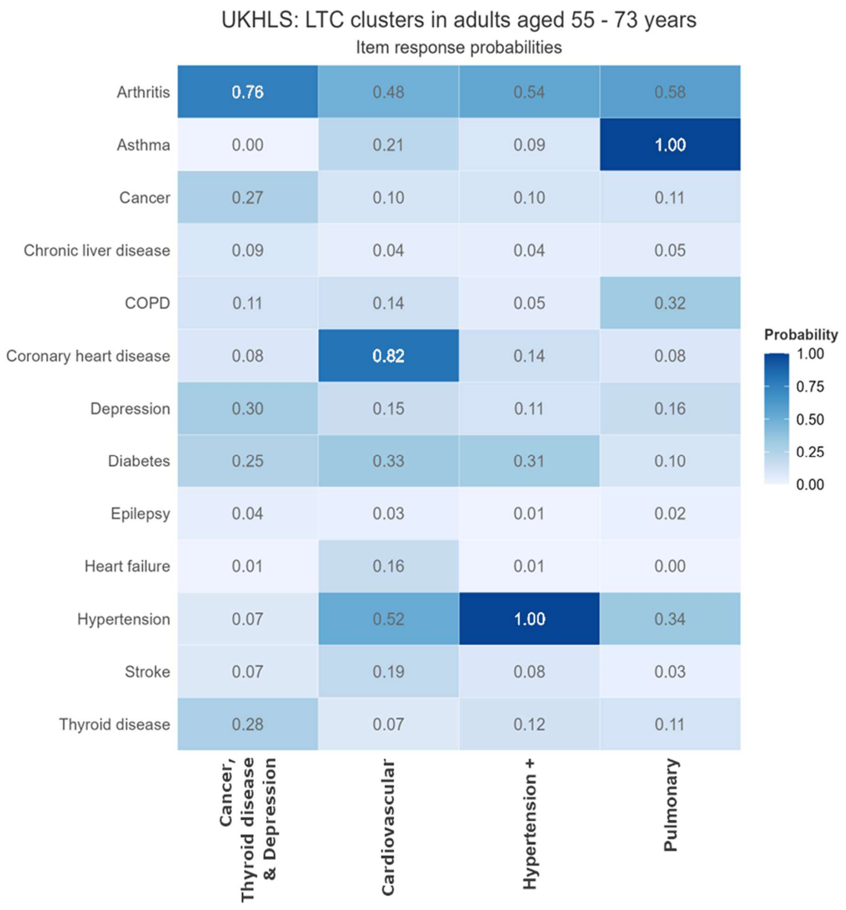

Figure S3.19. Within cluster conditional item response probabilities for adults 55 - 73 years in UKHLS.

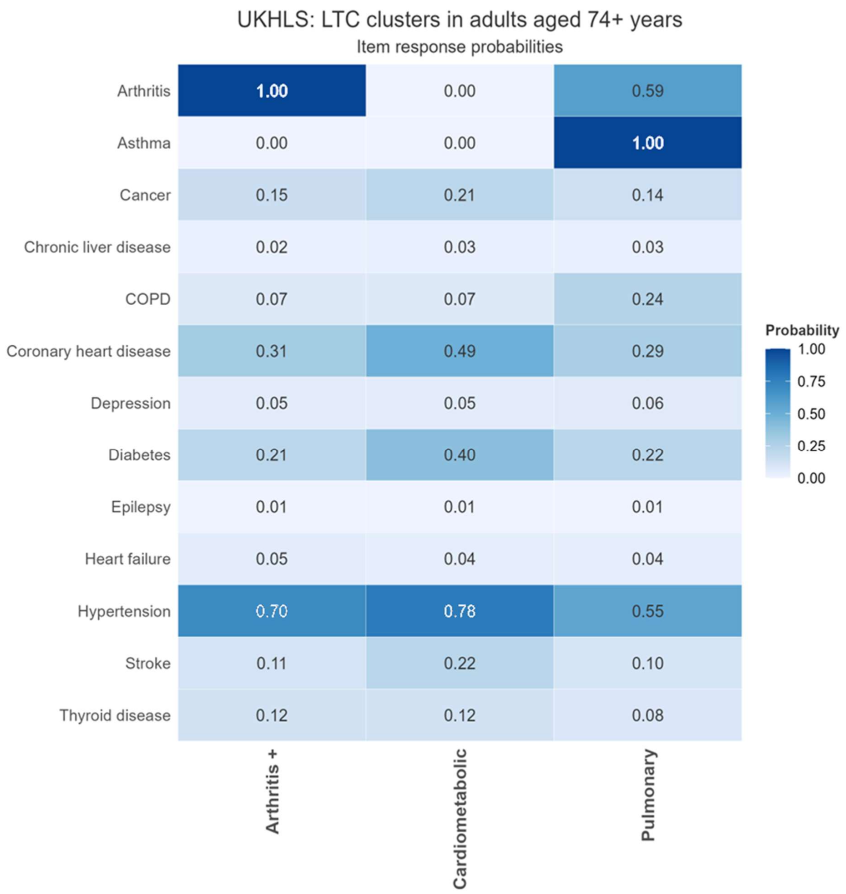

Figure S3.20.

Within cluster conditional item response probabilities for adults 74+ years in UKHLS.

MLTC Clusters – Sociodemographic characteristics

SAIL

SAIL: 18-36 years

Table S3.2. Sociodemographic characteristics of adults 18-36 years in SAIL stratified by multimorbidity clusters.

	No multimorbidity	Asthma+	Pain+ (incl. Migraine)	Substance misuse & Mental health	Depression+	Discordant
N (%)	503,188 (90.3)	8,085 (1.5)	10,321 (1.9)	11,935 (2.1)	17,698 (3.2)	5,779 (1.0)
Predicted probability of cluster membership	--	0.88 (0.14)	0.84 (0.12)	0.86 (0.19)	0.92 (0.10)	0.95 (0.12)
Female, n (%)	235,864 (46.9)	5,240 (64.8)	7,280 (70.5)	4,425 (37.1)	13,445 (76.0)	3,597 (62.2)
Age, years (SD)	26.9 (5.5)	28.9 (5.2)	30.1 (4.9)	29.5 (4.7)	29.8 (4.8)	29.9 (5.1)
Number of LTCs, median (IQR) [long list n=43]	0 (0, 0)	2 (2, 2)	3 (2, 3)	2 (2, 3)	2 (2, 2)	2 (2, 2)
Smoking, n (%)						
Never	299,015 (63.4)	4,155 (51.4)	4,746 (46.1)	1,531 (13.0)	8,394 (47.6)	3,829 (66.6)
Previous	134,116 (28.4)	3,164 (39.1)	4,377 (42.5)	7,847 (66.6)	7,464 (42.4)	1,615 (28.1)
Current	38,487 (8.2)	764 (9.5)	1,168 (11.4)	2,402 (20.4)	1,765 (10.0)	306 (5.3)
Deprivation score[1], median (IQR)	19.02 (11.4, 32.0)	21.6 (12.9, 35.2)	24.6 (15.0, 38.8)	29.4 (17.8, 43.9)	21.7 (13.1, 35.4)	19.5 (11.9, 32.7)
Deprivation rank[2,] median (IQR)	875 (413, 1387)	752 (334, 1283)	621 (257, 1112)	478 (190, 934)	739 (330, 1269)	851 (390, 1349)

Continuous data presented as mean (SD) unless otherwise stated, categorical data as n (%).[1] Welsh Government measure of relative multiple deprivation for small areas. Deprivation score (higher score=higher level of deprivation) rated across 8 types of deprivation employment, income, education, health, community safety, geographical access to services, housing, physical environment. [2] Welsh Government measure of relative multiple deprivation for small areas – relative ranking from 1 (most deprived area) to 1896 (least deprived area)

SAIL: adults 37 – 54 years

Table S3.3. Sociodemographic characteristics of adults 37 – 54 years in SAIL stratified by multimorbidity cluster.

	**No multimorbidity**	**Pulmonary**	**Pain+ (incl. Migraine & RA)**	**Substance misuse & Mental health**	**Cardiometabolic**	**Discordant**
N (%)	439,997 (75.9)	15,873 (2.7)	36,929 (6.4)	18,740 (3.2)	34,804 (6.0)	33,597 (5.8)
Predicted probability of cluster membership	--	0.78 (0.14)	0.78 (0.16)	0.86 (0.16)	0.81 (0.16)	0.75 (0.17)
Female, n (%)	203,289 (46.2)	9,567 (60.3)	25,275 (68.4)	7,841 (41.8)	15,846 (45.5)	23,833 (70.9)
Age, years	45.62 (5.04)	46.52 (5.08)	47.12 (4.96)	45.39 (5.05)	48.60 (4.67)	46.20 (5.09)
Number of LTCs, median (IQR) [long list n=43]	0 (0, 1)	2 (2, 3)	3 (2, 4)	3 (2, 3)	2 (2, 3)	2 (2, 2)
Smoking, n (%)						
Never	235,696 (58.1)	7,561 (47.7)	16,199 (44.3)	3,879 (21.0)	19,111 (55.10)	17,372 (52.4)
Previous	144,061 (35.5)	6,998 (44.1)	17,011 (46.5)	11,283 (61.0)	13,380 (38.6)	13,443 (40.5)
Current	25,750 (6.4)	1,298 (8.2)	3,398 (9.3)	3,350 (18.1)	2,194 (6.3)	2,360 (7.1)
Deprivation score [1], median (IQR)	16.9 (10.2, 27.5)	19.6 (11.6, 32.9)	23.1 (14.3, 36.1)	25.8 (15.6, 39.7)	20.1 (12.2, 33.0)	18.0 (10.9, 29.8)
Deprivation rank [2], median (IQR)	991 (524, 1485)	844 (386, 1361)	683 (299, 1178)	586 (240, 1072)	818 (382, 1331)	924 (465, 1431)

Continuous data presented as mean (SD) unless otherwise stated, categorical data as n (%).[1] Welsh Government measure of relative multiple deprivation for small areas. Deprivation score (higher score=higher level of deprivation) rated across 8 types of deprivation employment, income, education, health, community safety, geographical access to services, housing, physical environment. [2] Welsh Government measure of relative multiple deprivation for small areas – relative ranking from 1 (most deprived area) to 1896 (least deprived area)

SAIL: adults 55 – 73 years

Table S3.4. Sociodemographic characteristics of adults 55 - 73 years in SAIL stratified by multimorbidity cluster.

	No multimorbidity	Pulmonary	Pain+ (Incl. migraine & RA)	Mental health	Substance misuse & Discordant multimorbidity	Hypertension+	Cardio-metabolic	Cancer+
N (%)	243,746 (49.7)	23,796 (4.9)	42,802 (8.7)	40,803 (8.3)	9,001 (1.8)	84,063 (17.1)	24,734 (5.0)	21,694 (4.4)
Predicted probability of cluster membership	--	0.75 (0.20)	0.69 (0.18)	0.84 (0.17)	0.67 (0.20)	0.73 (0.14)	0.74 (0.19)	0.69 (0.17)
Female, n (%)	117,484 (48.2)	12,162 (51.1)	28,949 (67.6)	25,464 (62.4)	5,528 (61.4)	41,297 (49.1)	7,790 (31.5)	10,057 (46.4)
Age, years	62.8 (5.1)	65.4 (5.1)	64.0 (5.3)	63.5 (5.2)	65.74 (5.3)	65.0 (5.2)	66.6 (5.0)	65.6 (5.1)
Number of LTCs, median (IQR) [long list n=43]	0 (0, 1)	4 (3, 5)	3 (2, 4)	2 (2, 3)	7 (6, 8)	3 (2, 3)	4 (3, 5)	3 (2, 3)
Smoking, n (%)								
Never	128,359 (56.6)	5,912 (24.9)	20,321 (47.8)	20,809 (51.5)	2,849 (31.7)	43,937 (52.4)	10,339 (41.9)	11,787 (54.9)
Previous	87,718 (38.7)	15,787 (66.5)	19,860 (46.8)	19,860 (46.8)	5,486 (61.0)	36.827 (43.9)	13,373 (54.2)	8,932 (41.6)
Current	10,745 (4.5)	2,049 (8.6)	2,296 (5.4)	2.296 (5.4)	659 (7.3)	3,109 (3.7)	950 (3.9)	734 (3.4)
Deprivation score[1] , median (IQR)	15.6 (9.4, 24.5)	21.3 (13.4, 34.5)	20.5 (12.7, 33.2)	16.8 (10.5, 27.0)	24.5 (15.0, 38.1)	17.6 (10.8, 28.4)	18.7 (11.4, 30.5)	15.1 (9.2, 24.1)
Deprivation rank[2] , median (IQR)	1071 (623, 1533)	767 (352, 1239)	794 (376, 1294)	998 (537, 1496)	628 (268, 1118)	946 (497, 1435)	892 (447, 1382)	1107 (650, 1557)

Continuous data presented as mean (SD) unless otherwise stated, categorical data as n (%).[1] Welsh Government measure of relative multiple deprivation for small areas. Deprivation score (higher score=higher level of deprivation) rated across 8 types of deprivation employment, income, education, health, community safety, geographical access to services, housing, physical environment. [2] Welsh Government measure of relative multiple deprivation for small areas – relative ranking from 1 (most deprived area) to 1896 (least deprived area)

SAIL: adults (74+ years)

Table S3.5. Sociodemographic characteristics of adults 74+ years in SAIL stratified by multimorbidity cluster

	No multimorbidity	Pulmonary	Pain+	Mental health & Neurological	Hypertension+	Cardiometabolic	Cancer+
N (%)	44,799 (22.7)	15,432 (7.8)	28,832 (14.6)	16,136 (8.2)	61,886 (31.3)	13,220 (6.7)	17,399 (8.8)
Predicted probability of cluster membership	--	0.75 (0.22)	0.65 (0.17)	0.69 (0.20)	0.68 (0.18)	0.73 (0.20)	0.67 (0.20)
Female, n (%)	24,809 (55.4)	8,216 (53.2)	22,393 (77.7)	11,837 (73.4)	37,859 (61.2)	7,139 (54.0)	4,377 (25.2)
Age, years	80.4 (5.3)	80.7 (4.9)	81.3 (5.4)	83.3 (5.9)	81.4 (5.3)	82.8 (5.6)	81.1 (5.2)
Number of LTCs, median (IQR) [long list n=43]	1 (0, 1)	5 (4, 6)	4 (3, 5)	5 (4, 6)	3 (2, 3)	6 (5, 7)	3 (2, 4)
Smoking, n (%)							
Never	25,081 (63.9)	5,449 (35.4)	18,408 (64.4)	10,068 (62.8)	37,994 (61.6)	7,350 (55.8)	9,823 (56.9)
Previous	13,265 (33.8)	9,400 (61)	9,744 (34.1)	5,651 (35.3)	22,762 (36.9)	5,680 (43.1)	7,198 (41.7)
Current	897 (2.3)	552 (3.6)	450 (1.6)	304 (1.9)	927 (1.5)	148 (1.1)	249 (1.4)
Deprivation score[1] , median (IQR)	16.3 (9.7, 26.2)	19.4 (11.9, 31.8)	17.1 (10.5, 27.4)	17.8 (10.8 -29.0)	16.8 (10.4, 26.7)	17.5 (10.9, 28.1)	14.9 (8.9, 23.2)
Deprivation rank[2] , median (IQR)	1032 (571, 1520)	858 (421, 1347)	977 (527, 1466)	937 (484, 1437)	997 (553, 1472)	954 (507, 1421)	1120 (681, 1581)

Continuous data presented as mean (SD) unless otherwise stated, categorical data as n (%).[1] Welsh Government measure of relative multiple deprivation for small areas. Deprivation score (higher score=higher level of deprivation) rated across 8 types of deprivation employment, income, education, health, community safety, geographical access to services, housing, physical environment. [2] Welsh Government measure of relative multiple deprivation for small areas – relative ranking from 1 (most deprived area) to 1896 (least deprived area)

UK BIOBANK

UK BIOBANK: adults 37 – 54 years

Table S3.6. Sociodemographic characteristics of adults 37 – 54 years in UK Biobank stratified by multimorbidity cluster

	**No multimorbidity**	**Pulmonary**	**Pain+**	**Depression & Anxiety**	**Hypertension & Cardiometabolic**	**Cancer, Thyroid disease & Rheumatoid Arthritis**
N (%)	151,975 (78.3)	9,680 (5.0)	5,072 (2.6)	5,250 (2.7)	16,126 (8.3)	6,006 (3.1)
Predicted probability of cluster membership	--	0.90 (0.16)	0.97 (0.11)	0.82 (0.15)	0.82 (0.16)	0.99 (0.05)
Female, n (%)	82,942 (54.6)	6,174 (63.8)	3,255 (64.2)	3,702 (70.5)	7,592 (47.1)	4,204 (70.0)
Age, years	47.5 (4.2)	47.7 (4.2)	48.6 (4.1)	47.9 (4.1)	49.2 (3.9)	48.4 (4.1)
Number of LTCs, median (IQR) [long list n=43]	0 (0, 1)	2 (2, 3)	2 (2, 3)	2 (2, 3)	2 (2, 3)	2 (2, 2)
Townsend score, median (IQR) [missing data for n=330]	-1.9 (-3.5, 0.9)	-1.5 (-3.3, 1.6)	-1.3 (-3.2, 1.8)	-0.8 (-3.0, 2.5)	-0.9 (-3.1, 2.2)	-1.5 (-3.4, 1.6)
White ethnicity, n (%) [missing data for n=1,149]	138,121 (91.4)	8,958 (93.0)	4,685 (92.8)	4,945 (94.7)	14,279 (89.1)	5,580 (93.4)
BMI, kg/m$^{2, mean}$ (SD) [missing data for n=1,225]	26.7 (4.6)	28.0 (5.6)	28.1 (5.4)	28.2 3 (6.0)	30.9 (6.2)	27.0 (5.2)
Smoking, n (%) [missing for n=983]						
Never	93,293 (61.7)	5,481 (56.9)	2,753 (54.6)	2,675 (51.2)	8,783 (54.7)	3,367 (56.3)
Previous	39,106 (25.9)	2,684 (27.9)	1,432 (28.4)	1,392 (26.6)	4,792 (29.9)	1,717 (28.7)
Current	18,802 (12.4)	1,465 (15.2)	854 (17.0)	1,161 (22.2)	2,475 (15.4)	894 (15.0)
Alcohol weekly units. median (IQR) [missing data for n=1,377]	9.5 (1.4, 21.0)	7.0 (0.0, 18.0)	6.0 (0.0, 18.0)	4.5 (0.0, 17.0)	9.0 (0.0, 24.0)	5.5 (0.0, 15.0)
Alcohol group, n (%) [missing data for n=1,377]						
Low risk (<15 units/week)	92,848 (61.5)	6,517 (67.8)	3,532 (70.1)	3,693 (71.1)	9,828 (61.4)	4,422 (74.2)

	No multimorbidity	Pulmonary	Pain+	Depression & Anxiety	Hypertension & Cardiometabolic	Cancer, Thyroid disease & Rheumatoid Arthritis
Increasing risk (14 – 35 units/week)	48,130 (31.9)	2,551 (26.5)	1,225 (24.3)	1,126 (21.7)	4,516 (28.2)	1,267 (21.3)
High risk (>35 units/week)	9,953 (6.6)	549 (5.7)	281 (5.6)	376 (7.2)	1,653 (10.3)	267 (4.5)
Physical Activity, n (%) [missing data for n= 2,867]						
High	25,984 (17.4)	1,175 (12.3)	446 (8.9)	355 (6.9)	1,256 (7.9)	544 (9.2)
Medium	110,615 (73.9)	7,113 (74.4)	3,726 (74.4)	3,799 (73.4)	11,697 (73.8)	4,570 (77.1)
Low	4,390 (2.9)	385 (4.0)	237 (4.7)	281 (5.4)	793 (5.0)	237 (4.0)
None	8,725 (5.8)	894 (9.3)	601 (12.0)	743 (14.4)	2,101 (13.3)	575 (9.7)
Frailty[1], n (%) [data missing for n=4,405]						
Robust	96,507 (64.9)	4,713 (49.7)	2,310 (46.6)	1,812 (35.4)	6,511 (41.6)	2,904 (49.5)
Pre-frail	50,005 (33.6)	4,183 (44.1)	2,195 (44.3)	2,720 (53.2)	7,757 (49.6)	2,652 (45.2)
Frail	2,128 (1.4)	589 (6.2)	448 (9.1)	584 (11.4)	1,372 (8.8)	314 (5.4)
Self-rated health, n (%) [data missing for n=1,575]						
Excellent	32,014 (21.2)	748 (7.8)	295 (5.9)	239 (4.6)	608 (3.8)	449 (7.6)
Fair	24,641(16.3)	3,083 (32.1)	1,710 (34.1)	1,929 (37.3)	6,269 (39.3)	1,934 (32.6)
Good	91,191 (60.5)	4,720 (49.2)	2,339 (46.6)	1,984 (38.3)	6,508 (40.8)	2,903 (48.9)
Poor	3,008 (2.0)	1,052 (11.0)	678 (13.5)	1,023 (19.8)	2,555 (16.0)	654 (11.0)

Continuous data presented as mean (SD) unless otherwise stated, categorical data as n (%). [1] Frailty according to the frailty phenotype.

UK BIOBANK: adults 55 – 73 years

Table S3.7. Sociodemographic characteristics of adults 55 – 73 years in UK Biobank stratified by multimorbidity cluster

	No multimorbidity	Pulmonary	Mental Health & Pain	Hypertension & Cardiometabolic	Cancer+
N (%)	185,265 (60.1)	20,624 (6.7)	33,165 (10.8)	52,740 (17.1)	16,460 (5.3)
Predicted probability of cluster membership	--	0.68 (0.15)	0.85 (0.15)	0.81 (0.14)	0.79 (0.12)
Female, n (%)	98,778 (53.3)	12,520 (60.7)	21,891 (66.0)	21,982 (41.7)	10,259 (62.3)
Age, years	61.6 (4.0)	62.1 (4.1)	62.3 (4.1)	63.1 (4.0)	63.2 (4.0)
Number of LTCs, median (IQR) [long list n=43]	1 (0, 1)	2 (2, 3)	2 (2, 3)	2 (2, 3)	2 (2, 3)
Townsend score, median (IQR) [missing data for n=293]	-2.5 (-3.8, -0.2)	-2.1 (-3.7, 0.6)	-2.1 (-3.6, 0.5)	-1.9 (-3.5, 1.1)	-2.3 (-3.7, 0.3)
White ethnicity, n (%) [missing data for n=1,627]	178,325 (96.8)	19,803 (96.5)	32,227 (97.7)	49,562 (94.4)	16,084 (98.0)
BMI (kg/m^2) [missing data for n=1879]	26.8 (4.2)	28.3 (5.1)	27.7 (4.8)	29.9 (5.2)	27.7 (4.7)
Smoking, n (%) [missing data for n=1,965]					
Never	99,657 (54.1)	10,286 (50.2)	16,207 (49.2)	23,017 (44.0)	7,929 (48.4)
Previous	68,417 (37.2)	8,570 (41.8)	13,358 (40.6)	24,488 (46.8)	7,052 (43.1)
Current	16,034 (8.7)	1,630 (8.0)	3,380 (10.3)	4,871 (9.3)	1,393 (8.5)
Alcohol weekly units. median (IQR) [missing data for n=2,019]	9.5 (1.7, 21.0)	7.5 (0.0, 18.0)	6.0 (0.0, 15.5)	9.0 (0.0, 22.0)	7.5 (0.0, 18.0)
Alcohol group, n (%) [missing data for n=2,019]					
Low risk (<15 units/week)	114,879 (62.4)	13,840 (67.5)	23,832 (72.4)	32,962 (62.9)	11,107 (67.8)
Increasing risk (14 – 35 units/week)	58,710 (31.9)	5,520 (26.9)	7,591 (23.1)	15,582 (29.8)	4,407 (26.9)
High risk (>35 units/week)	10,467 (5.7)	1,138 (5.6)	1,491 (4.5)	3,840 (7.3)	869 (5.3)
Physical Activity, n (%) [missing data for n=4,282]					
High	15,307 (8.4)	1,033 (5.1)	1,309 (4.0)	1,879 (3.6)	767 (4.7)

	No multimorbidity	Pulmonary	Mental Health & Pain	Hypertension & Cardiometabolic	Cancer+
Medium	153,650 (84.1)	16,455 (80.9)	26,800 (81.8)	41,491 (80.1)	13,473 (82.8)
Low	5,980 (3.3)	1,032 (5.1)	1,786 (5.5)	3,026 (5.8)	786 (4.8)
None	7,875 (4.3)	1,815 (8.9)	2,855 (8.7)	5,415 (10.5)	1,238 (7.6)
Frailty[1], n (%) [missing data for n=7,181]					
Robust	118,624 (65.4)	9,569 (47.6)	15,144 (46.8)	21,707 (42.4)	8,239 (51.1)
Pre-frail	60,012 (33.1)	9,131 (45.4)	14,790 (45.7)	24,777 (48.4)	6,953 (43.2)
Frail	2,688 (1.5)	1,419 (7.1)	2,422 (7.5)	4,675 (9.1)	923 (5.7)
Self-rated health, n (%) [missing data for n=1,910]					
Excellent	40,566 (22.0)	1,292 (6.3)	2,111 (6.4)	2,242 (4.3)	1,263 (7.7)
Fair	23,844 (12.9)	6,608 (32.3)	10,690 (32.5)	19,743 (37.7)	4,881 (29.9)
Good	117,591 (63.8)	10,786 (52.7)	17,237 (52.4)	24,716 (47.2)	8,971 (54.9)
Poor	2,302 (1.3)	1,800 (8.8)	2,854 (8.7)	5,614 (10.7)	1,228 (7.5)

Continuous data presented as mean (SD) unless otherwise stated, categorical data as n (%). [1] Frailty according to the frailty phenotype.

UKHLS

UKHLS: adults 18 – 36 years

Table S3.81. Sociodemographic characteristics of adults 18-36 years in UKHLS stratified by multimorbidity cluster

	No multimorbidity	Pulmonary	Depression & Asthma	Cardiometabolic
N (%)	15,416 (95.7)	270 (1.7)	168 (1.0)	251 (1.6)
Predicted probability of cluster membership	--	0.95 (0.07)	0.85 (0.13)	0.99 (0.07)
Female, n (%)	8,454 (54.8)	176 (65.2)	124 (73.8)	175 (69.7)
Age, years	27.3 (5.5)	28.2 (5.4)	27.9 (5.0)	30.2 (4.8)
Number of LTCs, median (IQR) [from short list n=13]	0 (0, 0)	2 (2, 2)	2 (2, 3)	2 (2, 2)
White ethnicity, n (%)	10,015 (68.3)	226 (83.7)	146 (86.9)	213 (84.9)
BMI (kg/m^2)	24.7 (4.8)	27.1 (6.6)	26.1 (6.7)	28.1 (7.9)
Smoking *, n (%)				
Never	4,483 (46.8)	64 (35.0)	29 (24.8)	65 (36.9)
Previous	2,570 (26.9)	52 (28.4)	41 (35.0)	38 (21.6)
Current	2,520 (26.3)	67 (36.6)	47 (40.2)	73 (41.5)
Alcohol *, n (%)				
Never, or up to 2 times/year	1,929 (24.4)	41 (26.1)	27 (29.0)	46 (31.1)
Up to 1-2 times/month	2,317 (29.3)	51 (32.5	30 (32.3)	50 (33.8)
1-4 times/week	3,221 (40.8)	56 (35.7)	29 (31.2)	40 (27.0)
Daily or almost daily	430 (5.5)	9 (5.7)	7 (7.5)	12 (8.1)
Health-related quality of life				
SF-12 Physical Component [1]	54.0 (7.0)	46.2 (11.7)	48.0 (12.2)	45.7 (12.8)
SF-12 Mental Component [2]	50.4 (9.4)	48.4 (10.2)	37.1 (12.3)	39.5 (13.1)
EQ-5D Index Score [3], median (IQR)	1.00 (0.80, 1.00)	0.80 (0.69, 1.00)	0.73 (0.69, 0.85)	0.73 (0.62, 0.85)
Self-rated health, n (%)				
Excellent	3,947 (25.7)	25 (9.3)	9 (5.4)	8 (3.2)

Commented [SK1]: @Lewis Steel define what's in brackets

	No multimorbidity	Pulmonary	Depression & Asthma	Cardiometabolic
Very good	5,838 (37.9)	56 (20.7)	28 (16.7)	47 (18.7)
Good	4,078 (26.5)	84 (31.1)	56 (33.3)	60 (23.9)
Fair	1,212 (7.9)	59 (21.9)	45 (26.8)	79 (31.5)
Poor	313 (2.0)	46 (17.0)	30 (17.9)	57 (22.7)

Continuous data presented as mean (SD) unless otherwise stated, categorical data as n (%). *Smoking and alcohol were not collected at baseline, data presented here from second wave of data collection. [1] Score of <50 recommended as cut off for determining a 'physical condition'. [2] Score < 42 may be indicative of clinical depression. [3] EQ-5D index score mapped from SF-12 using.

UKHLS: adults 37 – 54 years

Table S3.9. Sociodemographic characteristics of adults 37 - 54 years in UKHLS stratified by multimorbidity clusters.

	No multimorbidity	Pulmonary	Arthritis+	Diabetes & Hypertension	Cardiovascular	Cancer, Thyroid disease & Depression
N (%)	14,542 (86.9)	805 (4.8)	466 (2.8)	210 (1.3)	218 (1.3)	485 (2.9)
Predicted probability of cluster membership	--	0.99 (0.03)	0.99 (0.04)	0.81 (0.16)	0.92 (0.11)	0.99 (0.03)
Female, n (%)	7,758 (53.4)	553 (68.7)	313 (67.2)	109 (51.9)	98 (45.0)	315 (65.0)
Age, years	44.8 (5.0)	45.8 (5.2)	47.8 (4.8)	47.8 (5.0)	47.8 (4.8)	46.5 (5.1)
Number of LTCs, median (IQR) [from short list n=13]	0 (0, 1)	2 (2, 3)	2 (2, 3)	2 (2, 3)	3 (2, 4)	2 (2, 2)
White ethnicity, n (%)	11,054 (78.9)	693 (86.2)	388 (83.3)	108 (51.4)	174 (79.8)	414 (85.4)
BMI (kg/m^2)	26.5 (4.9)	28.4 (6.4)	28.9 (6.7)	31.3 (6.5)	29.8 (7.5)	28.8 (6.6)
Smoking*, n (%)						
Never	5,027 (47.4)	220 (35.4)	123 (34.0)	91 (59.9)	46 (28.1)	141 (36.7)
Previous	3,266 (30.8)	204 (32.9)	124 (34.3)	33 (21.7)	47 (28.7)	120 (31.3)
Current	2,321 (21.9)	197 (31.7)	115 (31.8)	28 (18.4)	71 (43.3)	123 (32.0)
Alcohol*, n (%)						
Never up to 2 times/year	1,786 (19.6)	149 (28.6)	95 (30.1)	47 (42,7)	43 (33.1)	99 (30.1)
Up to 1-2 times/month	2,057 (22.5)	145 (27.8)	79 (25.0)	29 (26.4)	34 (26.2)	75 (22.8)

	No multimorbidity	Pulmonary	Arthritis+	Diabetes & Hypertension	Cardiovascular	Cancer, Thyroid disease & Depression
1-4 times/week	4,116 (45.1)	162 (31.1)	97 (30.7)	28 (25.5)	42 (32.3)	115 (35.0)
Daily or almost daily	1,172 (12.8)	65 (12.5)	45 (14.2)	6 (5.5)	11 (8.5)	40 (12.1)
Health-related quality of life						
SF-12 Physical Component [1]	52.4 (8.8)	42.3 (13.5)	37 (13.4)	42.2 (12.3)	33.9 (13.1)	44.0 (13.0)
SF-12 Mental Component [2]	50.4 (9.5)	43.7 (13.5)	43.3 (13.6)	46.9 (11.4)	41.8 (13.3)	41.4 (13.1)
EQ-5D Index Score [3], median (IQR)	0.85 (0.80, 1.00)	0.73 (0.62, 0.85)	0.69 (0.29, 0.80)	0.73 (0.66, 0.85	0.66 (0.19, 0.76)	0.73 (0.62, 0.85)
Self-rated health, n (%)						
Excellent	3,063 (21.1)	42 (5.2)	16 (3.4)	5 (2.4)	3 (1.4)	22 (4.5)
Very good	5,196 (35.8)	125 (15.5)	52 (11.2)	18 (8.6)	13 (6.0)	69 (14.2)
Good	4,203 (28.9)	207 (25.7)	95 (20.4)	66 (31.4)	34 (15.6)	130 (26.8)
Fair	1,595 (11.0)	229 (28.5)	163 (35.0)	65 (31.0)	61 (28.0)	148 (30.5)
Poor	464 (3.2)	202 (25.1)	140 (30.0)	56 (26.7)	107 (49.1)	116 (23.9)

Continuous data presented as mean (SD) unless otherwise stated, categorical data as n (%). *Smoking and alcohol data were not collected at baseline, data presented here from second wave of data collection.

[1] Score of <50 recommended as cut off for determining a 'physical condition'. [2] Score < 42 may be indicative of clinical depression. [3] EQ-5D index score mapped from SF-12.

Commented [SK2]: @Lewis Steell what's in brackets?

UKHLS: adults 55 - 73 years

Table S3.10. Sociodemographic characteristics of adults 55 - 73 years in UKHLS stratified by multimorbidity clusters.

	**No multimorbidity**	**Pulmonary**	**Hypertension+**	**Cardiovascular**	**Cancer, Thyroid disease & Depression**
N (%)	8,191 (66.6)	730 (5.9)	2,162 (17.6)	584 (4.7)	641 (5.2)
Predicted probability of cluster membership	--	0.81 (0.21)	0.88 (0.15)	0.77 (0.16)	0.94 (0.07)
Female, n (%)	4,218 (51.5)	478 (65.5)	1,198 (55.4)	226 (38.7)	436 (68.0)
Age, years	62.7 (5.2)	63.4 (5.1)	64.6 (5.3)	65.4 (5.3)	63.1 (5.1)
Number of LTCs, median (IQR) [from short list n=13]	0 (0, 1)	3 (2, 3)	2 (2, 3)	3 (2, 4)	2 (2, 2)
White ethnicity, n (%)	7,120 (90.5)	684 (93.7)	1,913 (88.6)	529 (90.7)	606 (94.5)
BMI (kg/m^2)	26.4 (4.4)	27.9 (5.5)	28.8 (5.3)	28.9 (5.9)	27.4 (5.4)
Smoking, n (%)					
Never	2,547 (39.9)	189 (30.9)	646 (36.3)	120 (26.8)	188 (36.2)
Previous	2,816 (44.1)	299 (48.9)	856 (48.1)	232 (51.8)	224 (43.1)
Current	1,016 (15.9)	124 (20.3)	279 (15.7)	96 (21.4)	108 (20.8)
Alcohol frequency, n (%)					
Never, or up to 2 times/year	1,127 (19.8)	169 (30.8)	459 (29.1)	153 (40.7)	132 (28.6)
Up to 1-2 times/month	1,029 (18.0)	115 (21.0)	299 (19.0)	64 (17.0)	110 (23.8)
1-4 times/week	2,454 (43.0)	180 (32.9)	519 (32.9)	108 (28.7)	151 (32.7)
Daily or almost daily	1,095 (19.2)	84 (15.3)	300 (19.0)	51 (13.6)	69 (14.9)
Health-related quality of life					
SF-12 Physical Component [1]	49.7 (10.3)	38.4 (14.0)	38.7 (13.5)	30.7 (13.1)	39.3 (13.7)
SF-12 Mental Component [2]	52.9 (8.7)	48.9 (12.4)	50.4 (11.3)	48.2 (12.6)	47.9 (12.6)
EQ-5D Index Score [3], median (IQR)	0.85 (0.78, 1.00)	0.73 (0.62, 0.81)	0.73 (0.62, 0.85)	0.66 (0.20, 0.76)	0.73 (0.62, 0.80)
Self-rated health, n (%)					
Excellent	1,441 (17.7)	25 (3.4)	68 (3.2)	2 (0.3)	27 (4.2)

Commented [SK3]: @Lewis Steel what's in brackets?

	No multimorbidity	Pulmonary	Hypertension+	Cardiovascular	Cancer, Thyroid disease & Depression
Very good	2,771 (33.9)	115 (15.8)	310 (14.4)	41 (7.0)	105 (16.4)
Good	2,378 (29.1)	193 (26.5)	667 (30.9)	84 (14.4)	185 (28.9)
Fair	1,184 (14.5)	212 (29.1)	677 (31.3)	187 (32.0)	195 (30.4)
Poor	390 (4.8)	184 (25.2)	439 (20.3)	270 (46.2)	129 (20.1)

Continuous data presented as mean (SD) unless otherwise stated, categorical data as n (%). *Smoking and alcohol data were not collected at baseline, data presented here from second wave of data collection. [1] Score of <50 recommended as cut off for determining a 'physical condition'. [2] Score < 42 may be indicative of clinical depression. [3] EQ-5D index score mapped from SF-12.

UKHLS: adults 74+ years

Table S3.11. Sociodemographic characteristics of adults 74+ years in UKHLS stratified by multimorbidity clusters.

	**No multimorbidity**	**Pulmonary**	**Arthritis+**	**Cardiometabolic**
N (%)	2,161 (53.4)	380 (9.4)	959 (23.7)	547 (13.5)
Predicted probability of cluster membership	--	1.00 (0)	1.00 (0)	1.00 (0)
Female, n (%)	1,131 (52.3)	232 (61.1)	633 (66.0)	230 (42.1)
Age, years	80.1 (5.0)	79.8 (4.5)	80.2 (4.8)	79.7 (4.8)
Number of LTCs, median (IQR) [from short list n=13]	1 (0, 1)	3 (2, 4)	3 (2, 3)	2 (2, 3)
White ethnicity, n (%)	1,900 (94.0)	356 (93.7)	901 (94)	510 (93.2)
BMI (kg/m^2)	25.2 (4.1)	26.6 (5.2)	26.9 (5.0)	26.2 (4.6)
Smoking*, n (%)				
Never	653 (43.1)	94 (32.4)	304 (42.2)	155 (37.4)
Previous	752 (49.6)	169 (58.3)	371 (51.5)	237 (57.3)
Current	110 (7.3)	27 (9.3)	46 (6.4)	22 (5.3)
Alcohol*, n (%)				
Never up to 2 times/year	388 (31.0)	98 (41.7)	234 (39.4)	119 (35.4)
Up to 1-2 times/month	242 (19.3)	50 (21.3)	114 (19.2)	75 (22.3)
1-4 times/week	394 (31.5)	49 (20.9)	150 (25.3)	87 (25.9)
Daily or almost daily	228 (18.2)	38 (16.2)	96 (16.2)	55 (16.4)
Health-related quality of life				
SF-12 Physical Component [1]	43.1 (12.4)	30.4 (12.5)	31.1 (12.4)	37.1 (12.8)
SF-12 Mental Component [2]	53.1 (9.4)	50.0 (11.5)	51.2 (11.5)	51.7 (10.4)
EQ-5D Index Score [3], median (IQR)	0.80 (0.69, 1.00)	0.69 (0.23, 0.80)	0.69 (0.59, 0.80)	0.73 (0.66, 0.85)
Self-rated health, n (%)				
Excellent	261 (12.2)	6 (1.6)	31 (3.2)	14 (2.6)

	No multimorbidity	Pulmonary	Arthritis+	Cardiometabolic
Very good	579 (26.9)	41 (10.8)	140 (14.6)	80 (14.7)
Good	636 (29.6)	63 (16.6)	217 (22.7)	150 (27.5)
Fair	438 (20.4)	129 (34.0)	327 (34.1)	189 (34.7)
Poor	235 (10.9)	141 (37.1)	243 (25.4)	112 (20.6)

Continuous data presented as mean (SD) unless otherwise stated, categorical data as n (%).*Smoking and alcohol data were not collected at baseline, data presented here from second wave of data collection. [1] Score of <50 recommended as cut off for determining a 'physical condition'. [2] Score < 42 may be indicative of clinical depression. [3] EQ-5D index score mapped from SF-12.

Discussion

Using LCA we identified latent classes of LTCs across three databanks and four age groups. Clusters identified in different age groups in all databanks center around specific systems in the body. For example, in all age groups and databanks LCA identified clusters centring around pulmonary conditions. Other common clusters combine cardiometabolic diseases, mental health or neurological conditions, or painful conditions. Although the exact combination of LTCs in clusters necessarily differ depending on the data used for LCA, previous studies applying LCA to different cohorts or databanks have likewise consistently identified clusters combining conditions around different systems including among others, cardiometabolic clusters, pain-related clusters, respiratory clusters, and neurological/mental health clusters [3,26,27,29,41]. In addition to clusters which group disorders affecting specific systems, discordant clusters are clusters grouping disorders with different aetiologies and affected systems which may have a more complex interpretation. Despite the differences in details on how many conditions were considered and how the conditions were entered into the databanks and cohorts between previous studies, as well as between the different databanks in this study, similar types of clusters tend to emerge across cohorts/databanks. These similarities in clusters suggest that there might be important associations and potentially connected aetiologies between certain LTCs, leading them to cluster together more frequently.

References

1. Excellence NIfHaC. Multimorbidity: clinical assessment and management. 2016. https://www.nice.org.uk/guidance/ng56 (accessed April 2023.
2. Chowdhury SR, Chandra Das D, Sunna TC, Beyene J, Hossain A. Global and regional prevalence of multimorbidity in the adult population in community settings: a systematic review and meta-analysis. *eClinicalMedicine* 2023; **57**.
3. Zhu Y, Edwards D, Mant J, Payne RA, Kiddle S. Characteristics, service use and mortality of clusters of multimorbid patients in England: a population-based study. *BMC Med* 2020; **18**(1): 78.
4. Barnett K, Mercer SW, Norbury M, Watt G, Wyke S, Guthrie B. Epidemiology of multimorbidity and implications for health care, research, and medical education: a cross-sectional study. *Lancet Glob Health* 2012; (1474-547X (Electronic)).
5. Jani BD, Hanlon P, Nicholl BI, et al. Relationship between multimorbidity, demographic factors and mortality: findings from the UK Biobank cohort. *BMC Med* 2019; **17**(1): 74.
6. Makovski TT, Schmitz S, Zeegers MP, Stranges S, van den Akker M. Multimorbidity and quality of life: Systematic literature review and meta-analysis. *Ageing Res Rev* 2019; **53**: 100903.
7. . Aoms. Multimorbidity: a priority for global health research: Academy of medical sciences; 2018.
8. Mair FS, May CR. Thinking about the burden of treatment. *BMJ : British Medical Journal* 2014; **349**: g6680.
9. Smith SM, Wallace E Fau - O'Dowd T, O'Dowd T Fau - Fortin M, Fortin M. Interventions for improving outcomes in patients with multimorbidity in primary care and community settings. 2016; (1469-493X (Electronic)).
10. Smith SM, Wallace E, Clyne B, Boland F, Fortin M. Interventions for improving outcomes in patients with multimorbidity in primary care and community setting: a systematic review. *Syst Rev* 2021; **10**(1): 271.
11. Research NIfHaC. Personalised Exercise-Rehabilitation FOR people with Multiple long-term conditions (multimorbidity)-The PERFORM trial. 2022. fundingawards.nihr.ac.uk/award/NIHR202020 (accessed May 2023.
12. Ho IS, Azcoaga-Lorenzo A, Akbari A, et al. Examining variation in the measurement of multimorbidity in research: a systematic review of 566 studies. *Lancet Public Health* 2021; **6**(8): e587-e97.
13. Johnston MC, Crilly M, Black C, Prescott GJ, Mercer SW. Defining and measuring multimorbidity: a systematic review of systematic reviews. *Eur J Public Health* 2019; **29**(1): 182-9.
14. Lee ES, Koh HL, Ho EQ, et al. Systematic review on the instruments used for measuring the association of the level of multimorbidity and clinically important outcomes. *BMJ Open* 2021; **11**(5): e041219.
15. Skou ST, Mair FS, Fortin M, et al. Multimorbidity. *Nat Rev Dis Primers* 2022; **8**(1): 48.
16. Busija L, Lim K, Szoeke C, Sanders KM, McCabe MP. Do replicable profiles of multimorbidity exist? Systematic review and synthesis. *European journal of epidemiology* 2019; **34**(11): 1025-53.
17. Barnett K, Mercer SW, Norbury M, Watt G, Wyke S, Guthrie B. Epidemiology of multimorbidity and implications for health care, research, and medical education: a cross-sectional study. *Lancet* 2012; **380**(9836): 37-43.
18. Townsend P. Deprivation. *Journal of Social Policy* 1987; **16**(2): 125-46.
19. Jones KH, Ford DV, Thompson S, Lyons RA. A Profile of the SAIL Databank on the UK Secure Research Platform. (2399-4908 (Electronic)).
20. Hanlon PA-O, Hannigan L, Rodriguez-Perez J, et al. Representation of people with comorbidity and multimorbidity in clinical trials of novel drug therapies: an individual-level participant data analysis. (1741-7015 (Electronic)).
21. Hanlon P, Jani BD, Nicholl B, Lewsey J, McAllister DA, Mair FS. Associations between multimorbidity and adverse health outcomes in UK Biobank and the SAIL Databank: A comparison of longitudinal cohort studies. *PLOS Medicine* 2022; **19**(3): e1003931.
22. University of Essex IfSaER. Understanding Society: Waves 1013, 2009-2022 and Harmonised BHPS: Waves 1-18, 1991-2009. [data collection]. *18th Edition UK Data Sercice* 2023; **SN: 6614**.
23. Linzer DA, Lewis JB. poLCA: An R Package for Polytomous Variable Latent Class Analysis. *Journal of Statistical Software* 2011; **42**(10): 1 - 29.
24. StataCorp. Stata Statistical Software: Release 17. In: StataCorp, editor. 17 ed. College Station, TX: College Station, TX: StataCorp LLC; 2021.
25. Bank TW. Life expectancy at birth, total (years) - united kingdom. 2019. https://data.worldbank.org/indicator/SP.DYN.LE00.IN?locations=GB (accessed 01.Jul 2023 2023).
26. Ho HE, Yeh CJ, Wei JC, Chu WM, Lee MC. Trends of Multimorbidity Patterns over 16 Years in Older Taiwanese People and Their Relationship to Mortality. *Int J Environ Res Public Health* 2022; **19**(6).
27. Nguyen QD, Wu C, Odden MC, Kim DH. Multimorbidity Patterns, Frailty, and Survival in Community-Dwelling Older Adults. *J Gerontol A Biol Sci Med Sci* 2019; **74**(8): 1265-70.

28. Olaya B, Moneta MV, Caballero FF, et al. Latent class analysis of multimorbidity patterns and associated outcomes in Spanish older adults: a prospective cohort study. *BMC Geriatr* 2017; **17**(1): 186.
29. Zheng DD, Loewenstein DA, Christ SL, et al. Multimorbidity patterns and their relationship to mortality in the US older adult population. *PLoS One* 2021; **16**(1): e0245053.
30. Weller BE, Bowen NK, Faubert SJ. Latent Class Analysis: A Guide to Best Practice. *Journal of Black Psychology* 2020; **46**(4): 287-311.
31. Collins LM, Lanza ST. Latent class and latent transition analysis: With applications in the social, behavioral, and health sciences: John Wiley & Sons; 2009.
32. Bayes-Marin I, Sanchez-Niubo A, Egea-Cortes L, et al. Multimorbidity patterns in low-middle and high income regions: a multiregion latent class analysis using ATHLOS harmonised cohorts. *BMJ Open* 2020; **10**(7): e034441.
33. Larsen FB, Pedersen MH, Friis K, Glumer C, Lasgaard M. A Latent Class Analysis of Multimorbidity and the Relationship to Socio-Demographic Factors and Health-Related Quality of Life. A National Population-Based Study of 162,283 Danish Adults. *PLoS One* 2017; **12**(1): e0169426.
34. Nichols L, Taverner T, Crowe F, et al. In simulated data and health records, latent class analysis was the optimum multimorbidity clustering algorithm. *J Clin Epidemiol* 2022; **152**: 164-75.
35. Whitson HE, Johnson KS, Sloane R, et al. Identifying Patterns of Multimorbidity in Older Americans: Application of Latent Class Analysis. *J Am Geriatr Soc* 2016; **64**(8): 1668-73.
36. Zhu Y, Edwards D, Mant J, Payne RA, Kiddle S. Characteristics, service use and mortality of clusters of multimorbid patients in England: a population-based study. *BMC medicine* 2020; **18**(1): 1-11.
37. Magidson J, Vermunt J. Latent class models for clustering: A comparison with K-means. *Canadian journal of marketing research* 2002; **20**(1): 36-43.
38. Barnett K, Mercer SW, Norbury M, Watt G, Wyke S, Guthrie B. Epidemiology of multimorbidity and implications for health care, research, and medical education: a cross-sectional study. *The Lancet* 2012; **380**(9836): 37-43.
39. Nylund KL, Asparouhov T, Muthén BO. Deciding on the number of classes in latent class analysis and growth mixture modeling: A Monte Carlo simulation study. *Structural equation modeling: A multidisciplinary Journal* 2007; **14**(4): 535-69.
40. Nylund-Gibson K, Choi AY. Ten frequently asked questions about latent class analysis. *Translational Issues in Psychological Science* 2018; **4**: 440-61.
41. Simoes D, Araujo FA, Severo M, et al. Patterns and Consequences of Multimorbidity in the General Population: There is No Chronic Disease Management Without Rheumatic Disease Management. *Arthritis Care Res (Hoboken)* 2017; **69**(1): 12-20.

Supplement 4: Years of life lost, length of hospitalisation, and number of GP visits ranking of clusters in SAIL and UK Biobank

Table S4.1: SAIL and UK Biobank LTC clusters ranked by predicted Years of Life Lost (YLL)

Cluster	Participants in cluster	Prevalence in databank	Mean age (years)	SD	Predicted Mortality*	95% CI		YLL	95% CI[†]		Rank	YLL x prevalence	Rank with Prevalence
S18_No multimorbidity	503187	0.276	26.92	5.46	0.00	0.004	0.005	0.23	0.21	0.25	33	0.063	33
S18_Asthma+	8085	0.004	28.87	5.24	0.01	0.004	0.008	0.32	0.23	0.40	32	0.001	32
S18_Pain+ (incl. Migraine)	10321	0.006	30.08	4.94	0.02	0.016	0.022	0.97	0.82	1.11	2	0.005	19
S18_Depression +	17698	0.010	29.80	4.75	0.01	0.009	0.013	0.55	0.46	0.64	28	0.005	28
S18_Substance misuse & Mental health	11935	0.007	29.48	4.72	0.03	0.025	0.032	1.48	1.30	1.67	1	0.010	11
S18_Discordant multimorbidity	5779	0.003	29.91	5.09	0.02	0.018	0.026	1.12	0.92	1.33	18	0.004	16
S37_No multimorbidity	439997	0.241	45.62	5.04	0.02	0.014	0.022	0.66	0.51	0.77	24	0.158	24
S37_Pulmonary	15873	0.009	46.52	5.08	0.03	0.020	0.031	0.89	0.70	1.09	3	0.008	22
S37_Pain+ (Incl. Migraines and RA)	36929	0.020	47.12	4.96	0.04	0.030	0.046	1.29	1.02	1.55	15	0.026	13
S37_Substance misuse & Mental health	18740	0.010	45.39	5.05	0.08	0.064	0.098	2.90	2.30	3.51	5	0.030	2
S37_Cardiometabolic	34.804	0.000	48.61	4.67	0.03	0.028	0.042	1.13	0.90	1.37	17	0.000	15
S37_Discordant Multimorbidity	33597	0.018	46.20	5.09	0.03	0.022	0.033	0.95	0.75	1.16	22	0.018	21
B37_No multimorbidity	151,975	0.302	47.54	4.17	0.01	0.011	0.016	0.45	0.38	0.52	29	0.137	29
B37_Pulmonary	9,680	0.019	47.69	4.17	0.02	0.008	0.031	0.65	0.28	1.02	25	0.013	25
B37_Pain+	5,072	0.010	48.63	4.05	0.02	0.008	0.029	0.60	0.25	0.95	27	0.006	27
B37_Depression & Anxiety	5,250	0.010	47.92	4.12	0.02	0.008	0.029	0.61	0.25	0.96	26	0.006	26
B37_Hypertension & Cardiometabolic	16,126	0.032	49.20	3.88	0.03	0.011	0.039	0.81	0.35	1.26	23	0.026	23
B37_Cancer, Thyroid disease & RA	6,006	0.012	48.39	4.08	0.03	0.014	0.052	1.09	0.46	1.72	19	0.013	17
S55_No multimorbidity	243745	0.134	62.78	5.05	0.09	0.091	0.099	1.75	1.68	1.82	13	0.233	10
S55_Pulmonary	23796	0.013	65.41	5.10	0.18	0.167	0.184	2.77	2.64	2.90	6	0.036	3
S55_Pain+ (Incl. Migraines & Rheumatoid arthritis)	42801	0.023	63.98	5.27	0.14	0.133	0.146	2.40	2.29	2.51	9	0.056	6
S55_Mental Health	40803	0.022	63.52	5.18	0.14	0.137	0.150	2.53	2.42	2.65	7	0.057	4

S55_Substance misuse, Mental health & Complex multimorbidity	9001	0.005	65.74	5.25	0.21	0.196	0.219	3.20	3.03	3.38	4	0.016	1
S55_Hypertension+	40803	0.022	65.02	5.19	0.12	0.116	0.126	1.95	1.87	2.04	11	0.044	8
S55_Cardiometabolic	48063	0.026	66.60	5.01	0.17	0.164	0.179	2.50	2.39	2.62	8	0.066	5
S55_Cancer+	24734	0.014	65.57	5.10	0.14	0.128	0.142	2.11	2.01	2.22	10	0.029	7
B55_No multimorbidity	185,265	0.369	61.62	4.05	0.05	0.042	0.057	0.97	0.83	1.11	21	0.356	20
B55_Pulmonary	20,624	0.041	62.11	4.11	0.07	0.051	0.080	1.24	0.96	1.52	16	0.051	14
B55_Mental health & Pain	33,165	0.066	62.29	4.06	0.06	0.044	0.069	1.07	0.83	1.30	20	0.070	18
B55_Hypertension & Cardiometabolic	52,740	0.105	63.09	3.99	0.07	0.057	0.089	1.32	1.03	1.61	14	0.139	12
B55_Cancer+	16,460	0.033	63.17	3.96	0.10	0.078	0.123	1.81	1.40	2.21	12	0.059	9
S74_No multimorbidity	44797	0.025	80.43	5.27	0.47	0.459	0.475	0.36	0.35	0.36	30	0.009	30
S74_Pulmonary	15431	0.008	80.66	4.85	0.63	0.620	0.647	0.34	0.34	0.35	31	0.003	31
S74_Pain+	28832	0.016	81.33	5.42	0.56	0.548	0.566	-0.07	-0.07	-0.07	35	-0.001	35
S74_Mental health & Neurological disorders	16136	0.009	83.26	5.91	0.68	0.668	0.694	-1.40	-1.38	-1.43	38	-0.012	38
S74_Hypertension+	61885	0.034	81.36	5.25	0.56	0.553	0.565	-0.09	-0.09	-0.09	36	-0.003	36
S74_Cardiometabolic	13220	0.007	82.79	5.55	0.66	0.646	0.675	-1.05	-1.03	-1.07	37	-0.008	37
S74_Cancer+	17399	0.010	81.09	5.15	0.54	0.528	0.551	0.06	0.06	0.06	34	0.001	34

total obs in SAIL	1825281.00
total obs in Biobank	502503.00
Average life expectancy UK 2019 (World Bank):	81.20

* Model derived marginal effects with all covariates set to their mean in the cluster

† 95% CI for YLL was calculated based on the 95% CI of predicted mortally in the cluster (Table 2)

Notes:

YLL: predicted marginal effect*(avg. life expectancy-mean Age in this group)

YLL normed to UK live expectancy for any sex in 2019

Table S4.2: SAIL and UK Biobank LTC clusters ranked by predicted days spent in hospital

Cluster	Mean age (years)	SD	Predicted number of days in hospital*	95% CI		Rank
S18_No multimorbidity	26.92	5.46	3.33	3.31	3.34	38
S18_Asthma+	28.87	5.24	3.99	3.87	4.11	36
S18_Pain+ (incl. Migraine)	30.08	4.94	6.13	6.00	6.26	18
S18_Depression +	29.80	4.75	4.83	4.74	4.92	31
S18_Substance misuse & Mental health	29.48	4.72	4.92	4.81	5.03	30
S18_Discordant multimorbidity	29.91	5.09	6.07	5.90	6.24	19
S37_no multimorbidity	45.62	5.04	3.40	3.38	3.41	37
S37_Pulmonary	46.52	5.08	4.11	4.03	4.20	35
S37_Pain+ (Incl. Migraines and RA)	47.12	4.96	5.36	5.30	5.43	27
S37_Substance misuse & Mental health	45.39	5.05	5.21	5.12	5.30	28
S37_Cardiometabolic	48.61	4.67	4.72	4.65	4.79	33
S37_Discordant Multimorbidity	46.20	5.09	4.71	4.64	4.77	34
B37_No multimorbidity	47.54	4.17	10.41	9.92	10.90	11
B37_Pulmonary	47.69	4.17	18.45	16.00	20.90	9
B37_Pain+	48.63	4.05	14.87	12.82	16.91	10
B37_Depression & Anxiety	47.92	4.12	19.89	17.19	22.59	7
B37_Hypertension & Cardiometabolic	49.20	3.88	22.71	19.73	25.69	6
B37_Cancer, Thyroid disease & RA	48.39	4.08	25.93	22.38	29.48	5
S55_No multimorbidity	62.78	5.05	4.77	4.74	4.80	32
S55_Pulmonary	65.41	5.10	5.96	5.88	6.05	22
S55_Pain+ (Incl. Migraines & Rheumatoid arthritis)	63.98	5.27	6.57	6.50	6.64	14
S55_Mental Health	63.52	5.18	5.67	5.61	5.73	23
S55_Substance misuse, Mental health & Complex multimorbidity	65.74	5.25	7.31	7.14	7.47	13
S55_Hypertension+	65.02	5.19	5.52	5.47	5.56	25
S55_Cardiometabolic	66.60	5.01	6.24	6.16	6.33	17

S55_Cancer+	65.57	5.10	7.32	7.22	7.43	12
B55_No multimorbidity	61.62	4.05	19.68	18.36	20.99	8
B55_Pulmonary	62.11	4.11	26.79	24.44	29.14	4
B55_Mental health & Pain	62.29	4.06	27.91	25.49	30.33	3
B55_Hypertension & Cardiometabolic	63.09	3.99	30.96	28.29	33.64	2
B55_Cancer+	63.17	3.96	32.86	29.95	35.78	1
S74_No multimorbidity	80.43	5.27	4.99	4.93	5.04	29
S74_Pulmonary	80.66	4.85	5.99	5.90	6.07	21
S74_Pain+	81.33	5.42	6.41	6.34	6.48	15
S74_Mental health & Neurological disorders	83.26	5.91	5.46	5.38	5.54	26
S74_Hypertension+	81.36	5.25	5.56	5.52	5.60	24
S74_Cardiometabolic	82.79	5.55	6.06	5.96	6.25	20
S74_Cancer+	81.09	5.15	6.35	6.27	6.44	16

Table S4.3: Sail and UK Biobank LTC clusters ranked by number of GP visits

Cluster	Mean age (years)	SD	Predicted number of GP visits*	95% CI		Rank
S18_No multimorbidity	26.92	5.46	76.12	75.89	76.35	31
S18_Asthma+	28.87	5.24	163.52	159.19	167.84	24
S18_Pain+ (incl. Migraine)	30.08	4.94	218.48	214.23	222.73	17
S18_Depression +	29.80	4.75	152.79	150.23	155.34	25
S18_Substance misuse & Mental health	29.48	4.72	196.69	192.92	200.47	19
S18_Discordant multimorbidity	29.91	5.09	193.14	188.14	198.14	22
S37_No multimorbidity	45.62	5.04	109.80	109.80	110.12	26
S37_Pulmonary	46.52	5.08	211.27	208.17	214.36	18
S37_Pain+ (Incl. Migraines and RA)	47.12	4.96	259.05	256.74	261.37	10

S37_Substance misuse & Mental health	45.39	5.05	259.21	255.85	262.57	9
S37_Cardiometabolic	48.61	4.67	222.71	220.35	225.06	16
S37_Discordant Multimorbidity	46.20	5.09	196.60	195.72	198.48	20
B37_No multimorbidity	47.54	4.17	39.45	38.40	40.50	38
B37_Pulmonary	47.69	4.17	67.21	60.82	73.60	34
B37_Pain+	48.63	4.05	62.31	56.23	68.40	36
B37_Depression & Anxiety	47.92	4.12	63.87	57.70	70.04	35
B37_Hypertension & Cardiometabolic	49.20	3.88	71.27	64.56	77.98	32
B37_Cancer, Thyroid disease & RA	48.39	4.08	68.39	61.73	75.06	33
S55_No multimorbidity	62.78	5.05	173.21	172.66	173.77	23
S55_Pulmonary	65.41	5.10	261.26	258.99	263.54	8
S55_Pain+ (Incl. Migraines & Rheumatoid arthritis)	63.98	5.27	302.28	300.46	304.09	2
S55_Mental Health	63.52	5.18	271.79	270.11	273.47	5
S55_Substance misuse, Mental health & Complex multimorbidity	65.74	5.25	326.49	321.80	331.19	1
S55_Hypertension+	65.02	5.19	261.86	260.62	263.09	7
S55_Cardiometabolic	66.60	5.01	290.06	287.66	292.47	3
S55_Cancer+	65.57	5.10	232.86	230.83	234.89	15
B55_No multimorbidity	61.62	4.05	56.10	53.86	58.33	37
B55_Pulmonary	62.11	4.11	85.79	80.54	91.04	28
B55_Mental health & Pain	62.29	4.06	81.14	76.23	86.05	30
B55_Hypertension & Cardiometabolic	63.09	3.99	88.02	82.72	93.33	27
B55_Cancer+	63.17	3.96	81.40	76.36	86.43	29
S74_No multimorbidity	80.43	5.27	195.35	193.94	196.75	21
S74_Pulmonary	80.66	4.85	249.73	247.11	252.34	13
S74_Pain+	81.33	5.42	278.02	275.98	280.05	4
S74_Mental health & Neurological disorders	83.26	5.91	240.40	237.95	242.85	14
S74_Hypertension+	81.36	5.25	253.99	252.73	255.26	11
S74_Cardiometabolic	82.79	5.55	266.06	263.04	269.08	6
S74_Cancer+	81.09	5.15	252.10	249.69	254.50	12

Supplement 5: Sensitivity analysis:

We performed a sex-stratified analysis of the association of Clusters with outcomes in SAIL participants. Poisson and Negative binomial regressions were performed in SAIL (our largest and most representative cohort) stratified by sex of each age group and outcome. The same models were used as described in the main article, using the same covariates except for sex. Results for females and males are presented parallel to each other in table S5.2 and S5.3 for Mortality, Table S5.4 and S5.5 for Hospitalisations, and S5.6 and S5.7 for GP visit

Table S5.1 Mortality analysis females

Females, Mortality, SAIL				
18-36 years	IRR	P-value	95% Confidence interval	
**MLTC Clusters**				
Asthma+	1.71	0.004	1.19	2.45
Pain+ (Incl. Migraine)	5.82	<0.001	3.91	5.95
Depression+	3.49	<0.001	2.80	4.37
Substance misuse & Mental Health	6.70	<0.001	5.89	7.63
Discordant MLTCs	6.60	<0.001	5.27	8.27
**LTC count**				
2 LTCs	1.31	0.008	1.07	1.59
3 LTCs	2.41	<0.001	1.73	3.35
4 LTCs	3.84	<0.001	1.81	8.15
>4 LTCs	4.07	0.049	1.00	16.50
37-54 years	IRR	P-value	95% Confidence interval	
**MLTC Clusters**				
Pulmonary	1.52	<0.001	1.34	1.73
Pain+ (incl. Migraine & RA)	2.12	<0.001	1.95	2.30
Substance misuse & Mental health	4.57	<0.001	4.27	4.89
Cardiometabolic	2.02	<0.001	1.87	2.19
Discordant MLTCs	1.61	<0.001	1.45	1.79
**LTC count**				
2 LTCs	1.27	<0.001	1.18	1.36
3 LTCs	1.78	<0.001	1.62	1.96
4 LTCs	2.38	<0.001	2.05	2.76
>4 LTCs	2.97	<0.001	2.28	3.86
55-73 years	IRR	P-value	95% Confidence interval	
**MLTC Clusters**				
Pulmonary	1.76	<0.001	1.68	1.84
Pain+ (incl. Migraine & RA)	1.47	<0.001	1.40	1.53
Mental health	1.62	<0.001	1.56	1.69
Substance misuse & Discordant MLTCs	2.03	<0.001	1.90	2.16
Hypertension+	1.27	<0.001	1.23	1.32
Cardiometabolic	1.72	<0.001	1.65	1.79
Cancer+	1.39	<0.001	1.32	1.46
**LTC count**				
2 LTCs	1.19	<0.001	1.15	1.22
3 LTCs	1.45	<0.001	1.39	1.50
4 LTCs	1.60	<0.001	1.52	1.68
>4 LTCs	1.76	<0.001	1.65	1.88
74+ years	IRR	P-value	95% Confidence interval	
**MLTC Clusters**				
Pulmonary	1.32	<0.001	1.26	1.38
Pain+	1.20	<0.001	1.15	1.25
Mental Health & Neurological disorders	1.46	<0.001	1.39	1.52
Hypertension+	1.19	<0.001	1.16	1.23
Cardiometabolic	1.39	<0.001	1.33	1.45
Cancer+	1.16	<0.001	1.12	1.20
**LTC count**				
2 LTCs	1.05	<0.001	1.03	1.08
3 LTCs	1.00	<0.001	1.09	1.16
4 LTCs	1.01	<0.001	1.14	1.24
>4 LTCs	1.00	<0.001	1.19	1.32

Table S5.2 Mortality analysis males

**Males, Mortality, SAIL**				
18-36 years	IRR	P-value	95% Confidence interval	
**MLTC Clusters**				
Asthma+	1.14	0.513	0.77	1.70
Pain+ (Incl. Migraine)	3.99	<0.001	3.23	4.93
Depression+	1.85	<0.001	1.46	2.36
Substance misuse & Mental Health	7.19	<0.001	5.79	8.93
Discordant MLTCs	3.66	<0.001	2.72	4.92
**LTC count**				
2 LTCs	1.64	<0.001	1.31	2.04
3 LTCs	3.35	<0.001	2.40	4.70
4 LTCs	3.89	<0.001	1.82	8.27
>4 LTCs	9.45	<0.001	3.03	4.92
37-54 years	IRR	P-value	95% Confidence interval	
**MLTC Clusters**				
Pulmonary	1.33	<0.001	1.16	1.51
Pain+ (incl. Migraine & RA)	2.05	<0.001	1.90	2.22
Substance misuse & Mental health	4.37	<0.001	3.99	4.80
Cardiometabolic	1.82	<0.001	1.65	2.01
Discordant MLTCs	1.43	<0.001	1.30	1.57
**LTC count**				
2 LTCs	1.37	<0.001	1.26	1.48
3 LTCs	2.14	<0.001	1.94	2.36
4 LTCs	2.90	<0.001	2.51	3.34
>4 LTCs	3.46	<0.001	2.79	4.30
55-73 years	IRR	P-value	95% Confidence interval	
**MLTC Clusters**				
Pulmonary	1.96	<0.001	1.86	2.07
Pain+ (incl. Migraine & RA)	1.49	<0.001	1.42	1.55
Mental health	1.42	<0.001	1.36	1.49
Substance misuse & Discordant MLTCs	2.31	<0.001	2.17	2.46
Hypertension+	1.27	<0.001	1.22	1.33
Cardiometabolic	2.04	<0.001	1.93	2.16
Cancer+	1.49	<0.001	1.40	1.59
**LTC count**				
2 LTCs	1.21	<0.001	1.17	1.26
3 LTCs	1.46	<0.001	1.40	1.52
4 LTCs	1.65	<0.001	1.57	1.74
>4 LTCs	1.82	<0.001	1.70	1.95
74+ years	IRR	P-value	95% Confidence interval	
**MLTC Clusters**				
Pulmonary	1.39	<0.001	1.34	1.45
Pain+	1.20	<0.001	1.16	1.23
Mental Health & Neurological disorders	1.45	<0.001	1.41	1.51
Hypertension+	1.20	<0.001	1.16	1.23
Cardiometabolic	1.43	<0.001	1.37	1.49
Cancer+	1.14	<0.001	1.08	1.19
**LTC count**				
2 LTCs	1.05	<0.001	1.03	1.07
3 LTCs	1.12	<0.001	1.09	1.15
4 LTCs	1.18	<0.001	1.14	1.22
>4 LTCs	1.22	<0.001	1.17	1.28

Table S5.3 Hospitalisation analysis females

**Females, Hospitalisations, SAIL**				
18-36 years	IRR	P-value	95% Confidence interval	
**MLTC Clusters**				
Asthma+	1.28	<0.001	1.20	1.36
Pain+ (Incl. Migraine)	2.23	<0.001	1.14	2.33
Depression+	1.65	<0.001	1.58	1.72
Substance misuse & Mental Health	1.50	<0.001	1.45	1.55
Discordant MLTCs	2.45	<0.001	2.33	2.58
**LTC count**				
2 LTCs	1.10	<0.001	1.05	1.15
3 LTCs	1.26	<0.001	1.15	1.38
4 LTCs	1.65	<0.001	1.32	2.08
>4 LTCs	0.60	0.154	0.30	1.21
37-54 years	IRR	P-value	95% Confidence interval	
**MLTC Clusters**				
Pulmonary	1.33	<0.001	1.28	1.37
Pain+ (incl. Migraine & RA)	1.67	<0.001	1.63	1.70
Substance misuse & Mental health	1.57	<0.001	1.53	1.61
Cardiometabolic	1.51	<0.001	1.47	1.54
Discordant MLTCs	1.46	<0.001	1.43	1.50
**LTC count**				
2 LTCs	1.06	<0.001	1.04	1.08
3 LTCs	1.28	<0.001	1.24	1.32
4 LTCs	1.48	<0.001	1.39	1.57
>4 LTCs	1.82	<0.001	1.61	2.05
55-73 years	IRR	P-value	95% Confidence interval	
**MLTC Clusters**				
Pulmonary	1.22	<0.001	1.19	1.25
Pain+ (incl. Migraine & RA)	1.34	<0.001	1.31	1.37
Mental health	1.18	<0.001	1.16	1.21
Substance misuse & Discordant MLTCs	1.41	<0.001	1.36	1.47
Hypertension+	1.17	<0.001	1.16	1.19
Cardiometabolic	1.30	<0.001	1.27	1.32
Cancer+	1.56	<0.001	1.52	1.59
**LTC count**				
2 LTCs	1.05	<0.001	1.03	1.07
3 LTCs	1.23	<0.001	1.21	1.25
4 LTCs	1.34	<0.001	1.30	1.38
>4 LTCs	1.40	<0.001	1.35	1.46
74+ years	IRR	P-value	95% Confidence interval	
**MLTC Clusters**				
Pulmonary	1.17	<0.001	1.13	1.20
Pain+	1.27	<0.001	1.24	1.30
Mental Health & Neurological disorders	1.01	0.648	0.97	1.04
Hypertension+	1.10	<0.001	1.08	1.13
Cardiometabolic	1.18	<0.001	1.14	1.22
Cancer+	1.25	<0.001	1.22	1.28
**LTC count**				
2 LTCs	1.06	<0.001	1.04	1.08
3 LTCs	1.14	<0.001	1.11	1.16
4 LTCs	1.17	<0.001	1.14	1.21
>4 LTCs	1.28	<0.001	1.24	1.34

Table S5.4 Hospitalisation analysis males

**Males, Hospitalisations, SAIL**				
18-36 years	IRR	P-value	95% Confidence interval	
**MLTC Clusters**				
Asthma+	1.17	<0.001	1.13	1.21
Pain+ (Incl. Migraine)	1.73	<0.001	1.69	1.78
Depression+	1.42	<0.001	1.39	1.45
Substance misuse & Mental Health	1.45	<0.001	1.41	1.52
Discordant MLTCs	1.54	<0.001	1.49	1.59
**LTC count**				
2 LTCs	1.12	<0.001	1.08	1.14
3 LTCs	1.66	<0.001	1.58	1.75
4 LTCs	1.42	<0.001	1.23	1.63
>4 LTCs	3.02	<0.001	2.18	4.20
37-54 years	IRR	P-value	95% Confidence interval	
**MLTC Clusters**				
Pulmonary	1.13	<0.001	1.09	1.16
Pain+ (incl. Migraine & RA)	1.52	<0.001	1.49	1.55
Substance misuse & Mental health	1.50	<0.001	1.46	1.54
Cardiometabolic	1.27	<0.001	1.24	1.30
Discordant MLTCs	1.33	<0.001	1.31	1.36
**LTC count**				
2 LTCs	1.15	<0.001	1.13	1.17
3 LTCs	1.47	<0.001	1.43	1.51
4 LTCs	1.67	<0.001	1.59	1.75
>4 LTCs	2.04	<0.001	1.87	2.22
55-73 years	IRR	P-value	95% Confidence interval	
**MLTC Clusters**				
Pulmonary	1.29	<0.001	1.26	1.32
Pain+ (incl. Migraine & RA)	1.40	<0.001	1.38	1.42
Mental health	1.20	<0.001	1.18	1.22
Substance misuse & Discordant MLTCs	1.62	<0.001	1.57	1.67
Hypertension+	1.14	<0.001	1.13	1.16
Cardiometabolic	1.33	<0.001	1.29	1.36
Cancer+	1.50	<0.001	1.46	1.54
**LTC count**				
2 LTCs	1.07	<0.001	1.06	1.09
3 LTCs	1.21	<0.001	1.20	1.24
4 LTCs	1.35	<0.001	1.31	1.38
>4 LTCs	1.47	<0.001	1.41	1.53
74+ years	IRR	P-value	95% Confidence interval	
**MLTC Clusters**				
Pulmonary	1.24	<0.001	1.20	1.27
Pain+	1.30	<0.001	1.28	1.33
Mental Health & Neurological disorders	1.14	<0.001	1.11	1.16
Hypertension+	1.13	<0.001	1.11	1.15
Cardiometabolic	1.25	<0.001	1.22	1.28
Cancer+	1.29	<0.001	1.25	1.33
**LTC count**				
2 LTCs	1.06	<0.001	1.04	1.07
3 LTCs	1.18	<0.001	1.16	1.24
4 LTCs	1.25	<0.001	1.22	1.28
>4 LTCs	1.30	<0.001	1.26	1.34

Table S5.5 GP visits analysis females

Females, GP visits, SAIL				
18-36 years	IRR	P-value	95% Confidence interval	
**MLTC Clusters**				
Asthma+	2.64	<0.001	2.51	1.78
Pain+ (Incl. Migraine)	3.67	<0.001	3.53	3.83
Depression+	2.68	<0.001	2.58	2.78
Substance misuse & Mental Health	2.83	<0.001	2.75	2.91
Discordant MLTCs	3.38	<0.001	3.22	3.54
**LTC count**				
2 LTCs	1.18	<0.001	1.13	1.22
3 LTCs	1.48	<0.001	1.35	1.61
4 LTCs	1.72	<0.001	1.35	1.18
>4 LTCs	0.84	0.556	0.46	1.51
37-54 years	IRR	P-value	95% Confidence interval	
**MLTC Clusters**				
Pulmonary	2.05	<0.001	1.99	2.10
Pain+ (incl. Migraine & RA)	2.57	<0.001	2.58	2.62
Substance misuse & Mental health	2.45	<0.001	2.40	2.50
Cardiometabolic	2.15	<0.001	2.11	2.19
Discordant MLTCs	1.95	<0.001	1.91	1.99
**LTC count**				
2 LTCs	1.13	<0.001	1.11	1.14
3 LTCs	1.34	<0.001	1.31	1.38
4 LTCs	1.51	<0.001	1.42	1.60
>4 LTCs	1.75	<0.001	1.56	1.95
55-73 years	IRR	P-value	95% Confidence interval	
**MLTC Clusters**				
Pulmonary	1.48	<0.001	1.46	1.51
Pain+ (incl. Migraine & RA)	1.71	<0.001	1.69	1.73
Mental health	1.55	<0.001	1.53	1.57
Substance misuse & Discordant MLTCs	1.81	<0.001	1.76	1.86
Hypertension+	1.51	<0.001	1.50	1.52
Cardiometabolic	1.66	<0.001	1.64	1.68
Cancer+	1.37	<0.001	1.35	1.39
**LTC count**				
2 LTCs	1.09	<0.001	1.08	1.10
3 LTCs	1.20	<0.001	1.19	1.22
4 LTCs	1.27	<0.001	1.25	1.29
>4 LTCs	1.30	<0.001	1.26	1.34
74+ years	IRR	P-value	95% Confidence interval	
**MLTC Clusters**				
Pulmonary	1.28	<0.001	1.25	1.31
Pain+	1.40	<0.001	1.38	1.43
Mental Health & Neurological disorders	1.19	<0.001	1.16	1.21
Hypertension+	1.30	<0.001	1.28	1.31
Cardiometabolic	1.37	<0.001	1.34	1.40
Cancer+	1.31	<0.001	1.29	1.33
**LTC count**				
2 LTCs	1.05	<0.001	1.04	1.06
3 LTCs	1.11	<0.001	1.10	1.13
4 LTCs	1.13	<0.001	1.11	1.16
>4 LTCs	1.17	<0.001	1.13	1.20

Table S5.6 GP visits analysis males

**Males, GP visits, SAIL**				
18-36 years	IRR	P-value	95% Confidence interval	
**MLTC Clusters**				
Asthma+	1.86	<0.001	1.80	1.91
Pain+ (Incl. Migraine)	2.55	<0.001	2.50	2.60
Depression+	1.79	<0.001	1.76	1.82
Substance misuse & Mental Health	2.16	<0.001	2.10	2.22
Discordant MLTCs	2.01	<0.001	2.07	2.07
**LTC count**				
2 LTCs	1.16	<0.001	1.19	1.19
3 LTCs	1.51	<0.001	1.58	1.58
4 LTCs	1.73	<0.001	1.96	1.96
>4 LTCs	2.04	<0.001	2.76	2.76
37-54 years	IRR	P-value	95% Confidence interval	
**MLTC Clusters**				
Pulmonary	1.81	<0.001	1.78	1,84
Pain+ (incl. Migraine & RA)	2.24	<0.001	2.22	2.27
Substance misuse & Mental health	2.27	<0.001	2.23	2.31
Cardiometabolic	1.84	<0.001	1.81	1.87
Discordant MLTCs	1.70	<0.001	1.68	1.72
**LTC count**				
2 LTCs	1.12	<0.001	1.10	1.12
3 LTCs	1.34	<0.001	1.31	1.36
4 LTCs	1.52	<0.001	1.47	1.57
>4 LTCs	1.69	<0.001	1.59	1.80
55-73 years	IRR	P-value	95% Confidence interval	
**MLTC Clusters**				
Pulmonary	1.54	<0.001	1.52	1.56
Pain+ (incl. Migraine & RA)	1.76	<0.001	1.75	1.78
Mental health	1.58	<0.001	1.57	1.60
Substance misuse & Discordant MLTCs	1.93	<0.001	1.90	1.97
Hypertension+	1.51	<0.001	1.50	1.53
Cardiometabolic	1.70	<0.001	1.68	1.73
Cancer+	1.31	<0.001	1.30	1.33
**LTC count**				
2 LTCs	1.08	<0.001	1.08	1.09
3 LTCs	1.22	<0.001	1.21	1.23
4 LTCs	1.30	<0.001	1.28	1.23
>4 LTCs	1.32	<0.001	1.28	1.34
74+ years	IRR	P-value	95% Confidence interval	
**MLTC Clusters**				
Pulmonary	1.28	<0.001	1.26	1.30
Pain+	1.43	<0.001	1.41	1.45
Mental Health & Neurological disorders	1.25	<0.001	1.23	1.27
Hypertension+	1.30	<0.001	1.29	1.32
Cardiometabolic	1.36	<0.001	1.33	1.38
Cancer+	1.23	<0.001	1.21	1.26
**LTC count**				
2 LTCs	1.06	<0.001	1.05	1.07
3 LTCs	1.13	<0.001	1.11	1.14
4 LTCs	1.18	<0.001	1.16	1.20
>4 LTCs	1.20	<0.001	1.18	1.23
